# Age prediction from human blood plasma using proteomic and small RNA data: a comparative analysis

**DOI:** 10.18632/aging.204787

**Published:** 2023-06-20

**Authors:** Jérôme Salignon, Omid R. Faridani, Tasso Miliotis, Georges E. Janssens, Ping Chen, Bader Zarrouki, Rickard Sandberg, Pia Davidsson, Christian G. Riedel

**Affiliations:** 1 Department of Medicine, Integrated Cardio Metabolic Centre (ICMC), Karolinska Institutet, Huddinge 14157, Sweden; 2 Department of Biosciences and Nutrition, Karolinska Institutet, Huddinge 14157, Sweden; 3 Lowy Cancer Research Centre, School of Medical Sciences, University of New South Wales, Sydney, Australia; 4 Garvan Institute of Medical Research, Sydney, Australia; 5 Translational Science and Experimental Medicine, Research and Early Development, Cardiovascular, Renal and Metabolism, BioPharmaceuticals R&D, AstraZeneca, Gothenburg, Sweden; 6 Bioscience Metabolism, Research and Early Development, Cardiovascular, Renal and Metabolism, BioPharmaceuticals R&D, AstraZeneca, Gothenburg, Sweden; 7 Department of Cellular and Molecular Biology, Ludwig Institute for Cancer Research, Karolinska Institutet, Solna 17165, Sweden

**Keywords:** human blood plasma, small RNAs, proteomics, aging, age prediction

## Abstract

Aging clocks, built from comprehensive molecular data, have emerged as promising tools in medicine, forensics, and ecological research. However, few studies have compared the suitability of different molecular data types to predict age in the same cohort and whether combining them would improve predictions. Here, we explored this at the level of proteins and small RNAs in 103 human blood plasma samples. First, we used a two-step mass spectrometry approach measuring 612 proteins to select and quantify 21 proteins that changed in abundance with age. Notably, proteins increasing with age were enriched for components of the complement system. Next, we used small RNA sequencing to select and quantify a set of 315 small RNAs that changed in abundance with age. Most of these were microRNAs (miRNAs), downregulated with age, and predicted to target genes related to growth, cancer, and senescence. Finally, we used the collected data to build age-predictive models. Among the different types of molecules, proteins yielded the most accurate model (R² = 0.59 ± 0.02), followed by miRNAs as the best-performing class of small RNAs (R² = 0.54 ± 0.02). Interestingly, the use of protein and miRNA data together improved predictions (R^2^ = 0.70 ± 0.01). Future work using larger sample sizes and a validation dataset will be necessary to confirm these results. Nevertheless, our study suggests that combining proteomic and miRNA data yields superior age predictions, possibly by capturing a broader range of age-related physiological changes. It will be interesting to determine if combining different molecular data types works as a general strategy to improve future aging clocks.

## INTRODUCTION

Over the last 50 years, there has been a steady increase in life expectancy and a decline in birth rates around the world [1, 2]. Consequently, medical research has increasingly turned its attention to investigating the determinants of health and mortality in the elderly, most importantly aging and non-communicable age-related diseases. Aging is viewed as the functional decline of the human body over time, caused by damage accumulation and increasing loss of cellular and tissue homeostasis [3]. Interestingly, it is a plastic process whose rate can differ between individuals [4], with some experiencing a slower or faster functional decline than others. This translates into different expectations for their lifespan, health, and quality of life. This has led to growing interest in methods that allow for the prediction of human age from physiological markers, with the idea that such predictions provide a measure of “biological age”. Any difference between predicted and true chronological age, assuming the age model is accurate, would indicate whether a person has aged faster or slower than expected and thus has an altered risk of experiencing age-related complications. Such information could then be used in population studies to determine factors that influence human aging, or in personalized medicine to propose aging-preventive interventions (such as changes in nutrition or lifestyle) [5] or usage of aging-preventive pharmaceuticals (e.g., senolytics [6] or caloric restriction mimetics) to individuals with substantially higher than expected biological ages. Similarly, biological age prediction could be used to monitor therapeutic success in patients subjected to such aging-preventive interventions. And finally, accurate prediction of chronological age has applications beyond biological age estimation, such as in forensic science [7, 8], in the resolution of legal disputes [9], or in anthropological and ecological studies when the chronological age of individuals is unknown [10].

Over the last decade, several such age-predictive methods, commonly referred to as “aging clocks”, have been developed. These methods are widely based on machine-learning approaches and were derived from high-dimensional datasets of various types. The best-performing aging clocks reach coefficients of determination (R²) of around 0.9. These include clocks built from DNA methylation data [11–13], imaging data (facial [14, 15], structural MRI [16], cornea of the eye [17]), and protein data [18, 19]. Other less accurate aging clocks (R² between 0.4 and 0.7) have been built from blood tests [20], mRNA expression data [21–23], miRNA data [24], gut microbiome composition [25], as well as social and behavioral data [26]. Among these, the most appealing clocks are based on molecular omics data, where thousands of predictive features (i.e., molecules) are available, even across different tissues or species [27], lending them the greatest spectrum of possible applications.

Given the abundance of age-predictive studies that have used molecular omics data, it is remarkable that two critical points have rarely been addressed. First, previous work has suggested that many types of molecular omics data are suitable for age prediction, but some perform better than others. So far, this has only seldom been confirmed by studies within the same cohort [28–30]. This is a concern because differences in population size, population composition (e.g., gender, age, or ethnicity), and chosen data analysis strategy (i.e., modeling approach, validation scheme, etc.) can greatly influence predictions and estimations of models’ performances [31]. Existing studies comparing different measures of biological age in the same cohort have mostly focused on models derived from physical examination, self-reported questionnaires, or basic blood tests, since these measurements are often readily available in larger cohorts [32–34]. However, studies directly comparing aging clocks generated from high-throughput omics measurements are missing.

Second, it has rarely been addressed whether combining different types of high-throughput molecular omics measurements helps to build more accurate predictive models. To our knowledge, only a single study has investigated this matter [24]. The authors measured miRNA abundance in plasma samples from the FHS Offspring cohort, built an age predictor from this data, and compared it to previously published predictors built from either DNA methylation [35] or mRNA [21] data from the same cohort. The study showed that DNA methylation data (R² = 0.53) outperformed mRNA data (R² = 0.31) and miRNA data (R² = 0.25) for age predictions. When combining predictions from DNA methylation and mRNA data, the authors observed an improvement in prediction accuracy (R² = 0.57), and a combination of all three omics datasets improved the predictions even further (R² = 0.63). Although this study was the first to quantitatively compare different molecular omics-based aging clocks built from the same cohort and to show that combining different molecular omics data may improve age-prediction accuracy, it still had caveats, such as a limited age range of 50–80 years and relatively low R² values. Additionally, it did not try to address the potential reasons for the improved performance of the combined datasets.

**Figure 1:**
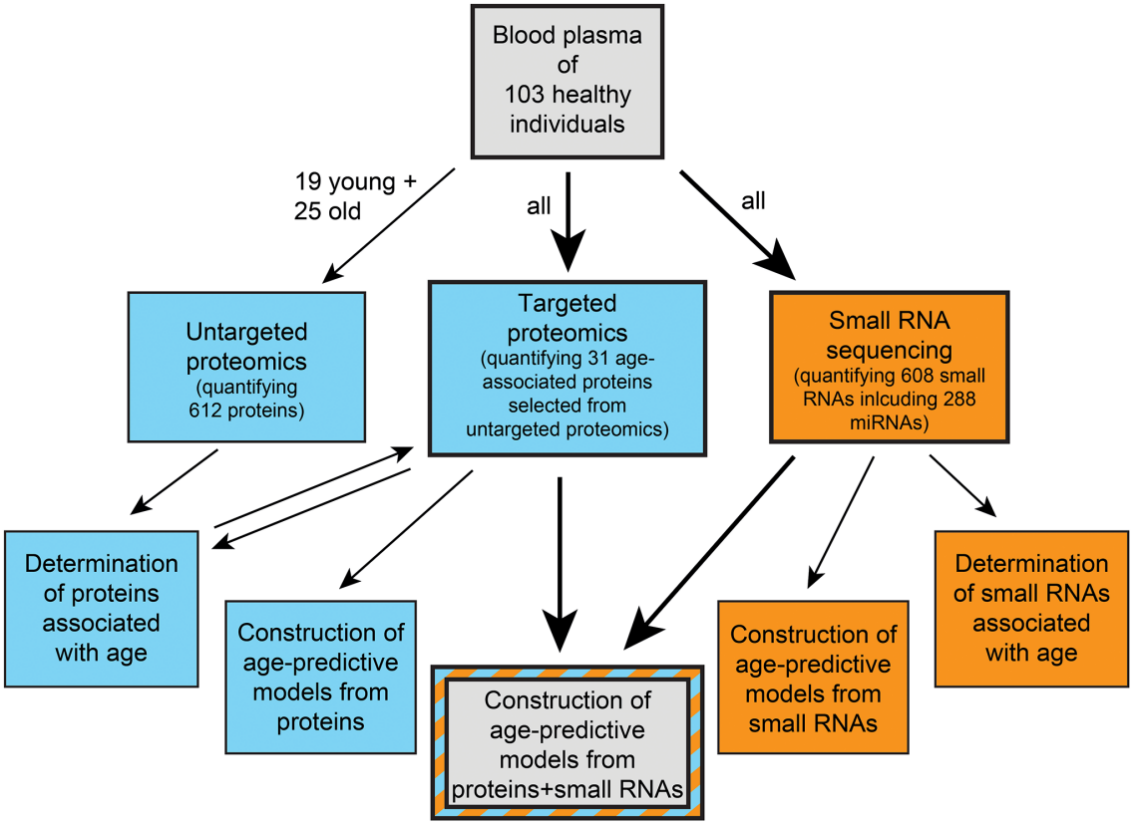
**Schematic overview of our study.** Blue indicates proteomics-based work and orange indicates small RNA-based work.

Here we expand the limited portfolio of comparisons between aging clocks built from different types of molecular data from the same cohort. We measure the abundances of proteins and a broad spectrum of small RNAs in the plasma of an age-stratified cohort of human individuals, determine age-associated molecules, build age-predictive models from various sets of omics measurements, and compare their performances. A schematic outline of our study can be found in Figure 1.

## RESULTS

### The study cohort

To investigate age-related molecular changes in humans, we used blood plasma from a cohort of 103 North American individuals aged between 20 and 83, with a mean age of 55 years (see Table 1). All individuals had no known diseases and were generally healthy when the samples were taken (the mean Mini-Mental State Examination (MMSE) score was 29, the mean Body Mass Index (BMI) was 27, and the mean blood pressure was 125/79; see Table 1). Blood samples were collected, and plasma was immediately prepared, frozen, and stored for later use.

**Table 1:** Overview of the cohort.

**Variable (unit)**	**Mean**	**S.D.**
Age (years)	55.30	17.50
Height (m)	1.76	0.10
Weight (kg)	83.40	15.50
BMI	26.80	4.85
Mini-Mental State Examination (MMSE)	29.40	0.93
Systolic blood pressure (mmHg)	125.00	13.50
Diastolic blood pressure (mmHg)	79.40	9.03
Pulse (bpm)	68.00	9.62
Body temperature (°C)	35.80	0.81

### Identification of age-associated changes in plasma proteins by untargeted proteomics

**Figure 2:**
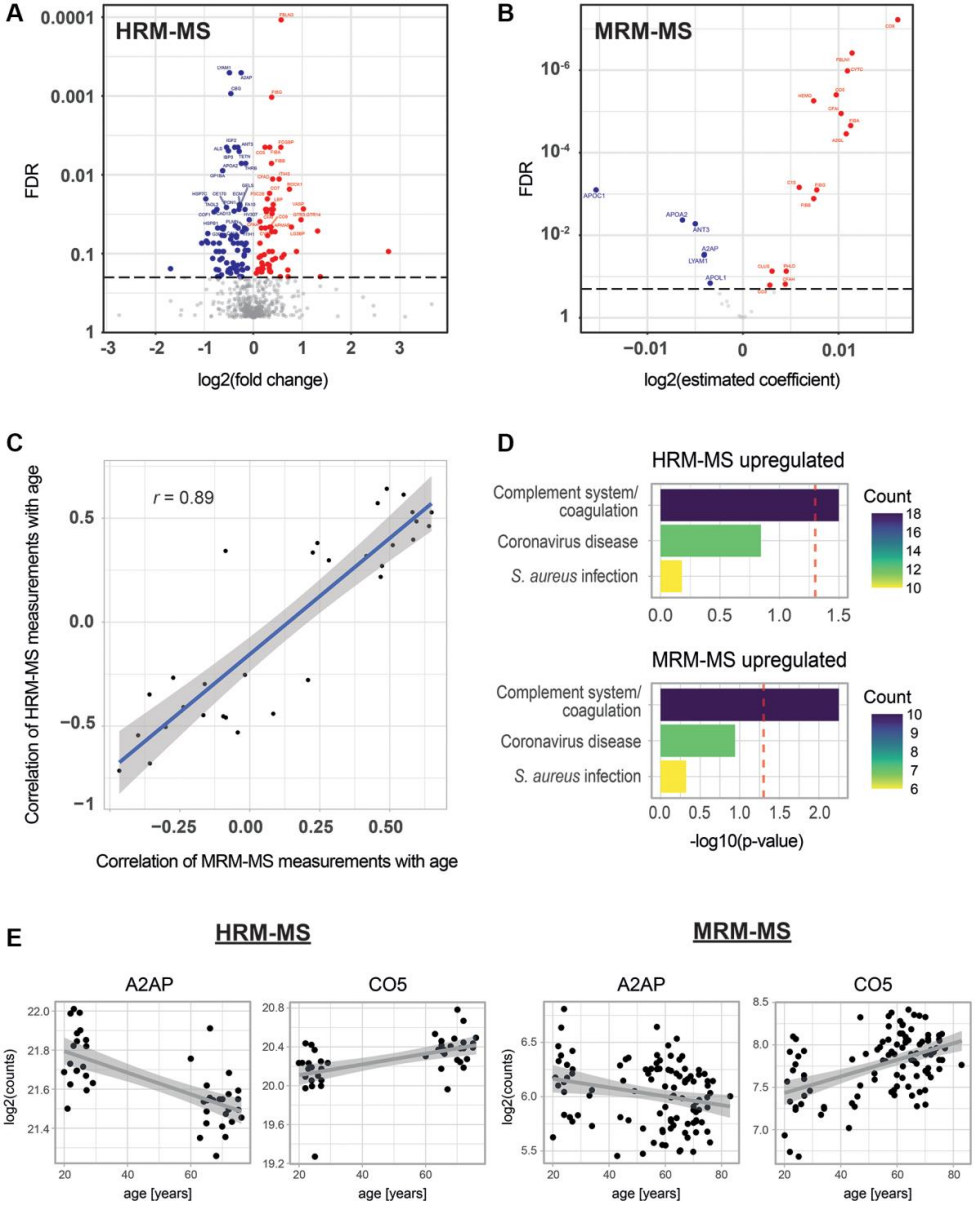
**Age-associated proteins in blood plasma.** (**A**) Age-dependent changes for 612 proteins as measured by HRM-MS (untargeted proteomics) in 19 young and 25 old individuals. The volcano plot shows the log2 fold change in protein abundance on the x-axis and the Benjamini-Hochberg (BH) FDR-corrected Mann-Whitney test on the y-axis. Red and blue colors highlight significantly up- and down-regulated proteins, respectively (FDR < 0.2). (**B**) Age-dependent changes for 31 proteins measured by MRM-MS (targeted proteomics) in 103 individuals. A linear model was fitted for each protein, with age as the dependent variable and the log of protein abundance as the independent variable. The volcano plot shows the estimated coefficients on the x-axis and the BH FDR on the y-axis. Red and blue colors highlight significantly up- and down-regulated proteins, respectively (FDR < 0.2). (**C**) Scatter plot of the correlation with age of MRM-MS measurements (x-axis) and HRM-MS (y-axis). Blue line and shadow: linear regression and 95% confidence interval, respectively. (**D**) KEGG pathway enrichment analysis for significantly up-regulated proteins from the MRM-MS and HRM-MS experiments. For both experiments, the set of all measured 612 proteins was used as a background to compute the significance of age-association. Colors indicate the number of age-associated proteins that are attributed to these pathways. (**E**) Examples of scatter plots for two proteins detected as age-associated in the two MS experiments.

First, we explored age-associated changes in the proteome of our blood plasma samples. Human blood plasma contains several thousand proteins [36], and subsets of them have already been used to predict age in humans [18, 19, 37]. However, the optimal choice of plasma proteins for age predictions remains a topic of debate. Therefore, we began by comprehensively defining the proteins that change in abundance with age in our cohort using Hyper Reaction Monitoring mass spectrometry (HRM-MS) [38]. HRM-MS is a state-of-the-art Data Independent Acquisition (DIA) untargeted proteomics method that overcomes the limitations of shotgun proteomics, such as missing annotations or low reproducibility and precision. We conducted these measurements in only a subset of our cohort, which comprised 19 young individuals (aged 20 to 30 years) and 25 old individuals (aged 65 to 76 years). Overall, we quantified 612 proteins, of which 145 showed differential abundance between the young and old age groups (60 and 85 were up- and down-regulated, respectively, with a False Discovery Rate (FDR) < 0.2; see Figure 2A, Supplementary Figure 1, and Supplementary Table 1). Next, we examined how many of our age-correlated proteins had been previously reported. When comparing them to the three most comprehensive studies of age-correlated plasma proteins available [18, 19, 39], we found that 31 of our age-associated proteins had been identified by at least one of these other studies (see Supplementary Table 1), leaving us with 114 potentially new age-associated proteins. These latter proteins include, for instance, complement components 3, 5 and 7 (C3, C5, C7), Fibrinogen Alpha and Beta chains (FGA, FGB), and Apolipoproteins A-II and C-I (APOA2, APOC1) (see Supplementary Table 1). Finally, we conducted a functional annotation enrichment analysis. No significant enrichments were observed among downregulated proteins. However, proteins that were upregulated with age were significantly enriched in components of the complement system (*p* < 0.05, Figure 2D, upper panel). Even though it did not pass our significance threshold, the Coronavirus disease 2019 (COVID-19)-related proteins term (Kyoto Encyclopedia of Genes and Genomes (KEGG) id: ko05171) was also enriched (with a corrected *p*-value of 0.143). This finding is intriguing given that COVID-19 leads to more severe symptoms in older individuals.

### Quantification of age-associated changes in plasma proteins by targeted proteomics across the entire cohort

Having identified 145 age-associated proteins, we then selected a representative subset of 31 proteins to be measured in our entire cohort of 103 individuals (Supplementary Table 2) using targeted Multiple Reaction Monitoring mass spectrometry (MRM-MS). As expected from our HRM-MS analysis, most of these proteins were also significantly associated with age (21 out of 31; FDR < 0.2) when measured using this different methodology and in the full cohort (Figure 2B, 2E, Supplementary Figure 3, Supplementary Table 2). The consistency between the untargeted and targeted mass spectrometry methods was further confirmed by observing a high correlation of all common measurements (*r* = 0.83, Supplementary Figure 2A) and of the proteins’ Pearson correlation with age (*r* = 0.89, Figure 2C). As external validation, we compared the beta coefficients from our study with those of another study that quantified age-dependent changes in plasma proteins [18] and found a Pearson correlation coefficient of 0.85 (see Supplementary Figure 2B). The 15 significantly upregulated proteins in this assay showed similar pathway enrichment as in HRM-MS, with the complement system being significantly enriched (Figure 2D, lower panel). Only six proteins were downregulated, and no functional enrichment term reached significance (data not shown). However, three of these six proteins were apolipoproteins (APOC1, APOA1 and APOL1), suggesting a down-regulation of this type of protein with age. Taken together, we established a set of 21 proteins that show age-dependent abundance changes by two independent MS methods, and we quantified these proteins by MRM-MS across the entire cohort.

### Quantification of age-associated changes in plasma small RNAs

**Figure 3:**
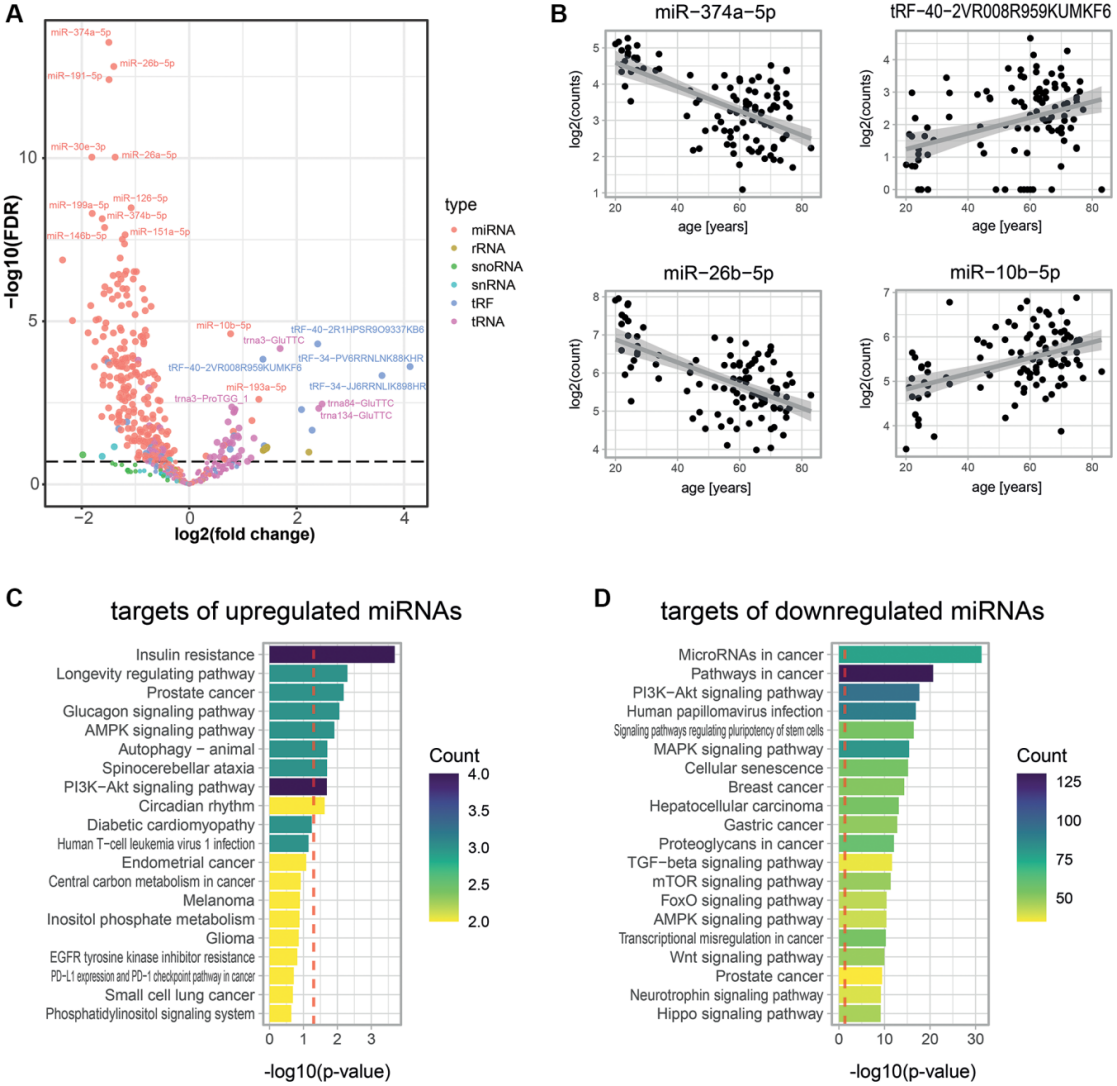
**Age-associated small RNAs in blood plasma.** (**A**) Age-dependent changes for 608 small RNAs as measured by Small-seq in 103 individuals. A negative binomial model was fitted for each RNA using DESeq2 [79]. The volcano plot shows log2 fold changes in expression between young and old individuals on the x-axis and log10 *p*-values of BH FDR-corrected Wald tests on the y-axis. The former was obtained by multiplying the log2 fold change in small RNA expression for 1 year (i.e., the estimate of the model) with the mean age difference between individuals from the young and old age groups of the untargeted proteomics experiments (i.e., 44.8 years). (**B**) Examples of scatter plots for four small RNAs detected as age-associated. (**C**, **D**) KEGG pathway enrichment analysis for predicted targets of significantly up- (**C**) and down- (**D**) regulated miRNAs. A robust analytic approach (see Methods) allowed us to select 22 and 2,159 miRNA targets that were up- and down-regulated with age, respectively. The set of all 26,194 human transcripts present in the multiMIR database was used as a background to compute the significance of age-association. The gprofiler2 R package was used to compute enrichment, and *p*-values were corrected using the gSCS correction method [85]. Colors show the number of targets of our age-associated miRNAs that are attributed to these pathways.

To complement the age-associated protein measurements, we decided to quantify total small RNAs in the same cohort. Although age predictions had been conducted using only miRNAs [24], no comprehensive small RNA transcriptomic dataset had been used to predict chronological age before. We acquired such data through the “Small-seq” method [40, 41], which enabled us to quantify 608 small RNAs in our plasma samples (Figure 3A). The most detected small RNAs were miRNAs (288 miRNAs) and transfer RNAs (229 tRNAs) (Figure 3A, see Methods). Out of all detected small RNAs, 70 and 245 significantly increased and decreased with age, respectively (FDR < 0.2; Figure 3A, 3B, Supplementary Figure 4, Supplementary Table 3). Strikingly, the 60 most significantly age-associated small RNAs (with an adjusted *p*-value below 4.5 × 10^−5^) were all age-decreasing miRNAs, and 74.2% of all measured miRNAs were significantly decreasing with age. Therefore, the decrease of miRNA with age appears to be a key feature of aging. Age-increasing small RNAs were more diverse, with the top 10 being comprised of miRNAs, tRNAs, and fragments of tRNAs (tRFs). To validate these results, we compared our set of age-associated miRNAs with those identified by a similar study [42]. We observed a significant overlap (*p* = 0.004, Supplementary Figure 5), illustrating the consistency of our measurements with previous work.

Circulating miRNAs can sometimes act like hormones by being secreted and eventually taken up by target cells, where they regulate gene expression [43]. Therefore, we hypothesize that circulating small RNAs that change in abundance with age could act as messengers of age-related physiological changes. To investigate this, we focused on the 110 most significantly downregulated miRNAs (FDR < 0.001) and the 7 most significantly upregulated miRNAs (FDR < 0.2), and first determined their predicted targets using the tool multiMiR [44]. We obtained 2,159 and 22 predicted targets for the down- and up-regulated miRNAs, respectively (see Methods). Then, we conducted functional annotation enrichment analyses on these targets. Remarkably, gene targets of age-elevated miRNAs were prominently enriched for the term insulin resistance, but also for nutrient-dependent signaling, longevity, and autophagy, all of which have a substantial impact on or relate to aging [45] (Figure 3C). Gene targets of age-depleted miRNAs were enriched for functions related to growth (Figure 3D), in particular cancer, an age-related morbidity, and senescence, a hallmark of aging [3].

### Construction of age-predictive models

**Figure 4:**
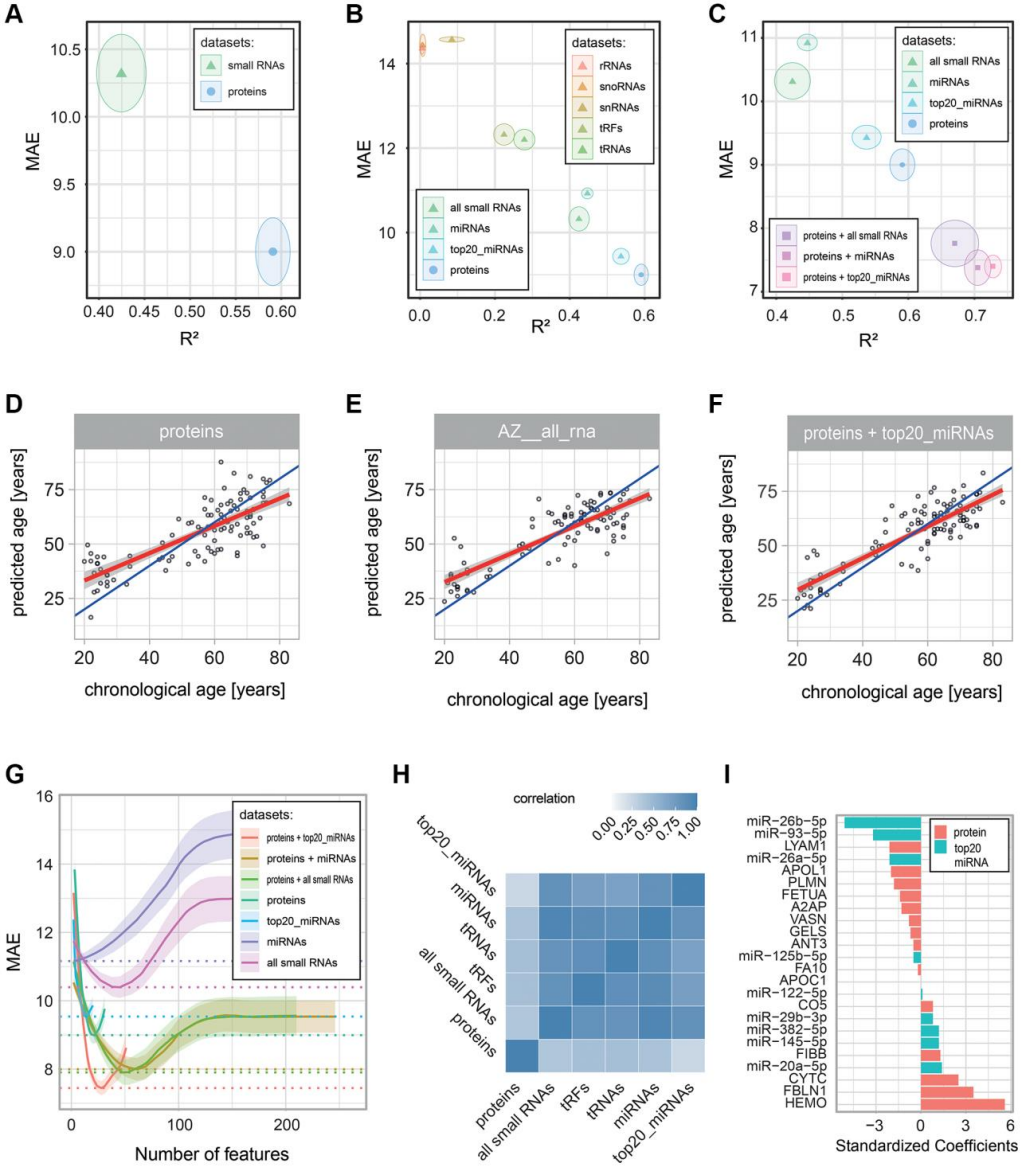
**Performance of age-predictive models built on various data types.** Age-predictive L1-norm penalized generalized linear models were built using protein and small RNA measurements, either separately or in combinations. Performance was estimated via 10-fold cross-validation with 100 repeats. Prediction errors were determined from predictions based on left-out data (data that was not used to build the model). (**A**–**C**) Performance of the built models: the mean (dot) and standard deviation (circle) of two error metrics are shown: the coefficient of determination (R^2^) on the x-axis and the Mean Absolute Error (MAE) on the y-axis. The panels compare (**A**) all small RNAs with all proteins, (**B**) the different classes of small RNAs, and (**C**) models combining proteins and small RNAs. (**D**–**F**) Scatter plots of chronological age vs. predicted age are shown for all individuals in the cohort for (**D**) the proteomics-based model, (**E**) the all small RNA-based model, and (**F**) the proteomics and top 20_miRNA-based model. Blue and red lines show, respectively, the identity and linear regression lines. (**G**) Plot of the number of predictive molecules kept in the model (with non-zero coefficients) on the x-axis vs. the mean (line) and standard deviation (shadow) MAE on the y-axis. MAE values were smoothed via a LOESS regression (R loess function with a span argument of 0.6). (**H**) Heatmap showing the correlation of the error in predictions (delta age) for the proteomics-based model and the small RNA-based models with R^2^ > 0.2. (**I**) Absolute standardized coefficients of the proteomics and top 20_miRNA-based models.

To study the impact of different types of molecules on age prediction, we determined the ability of proteins or small RNAs to predict chronological age. We built age-predictive L1-penalized generalized linear models with repeated cross-validation (see Methods). When we compared the resulting models, we found that both protein and small RNA data could be used to predict chronological age with reasonable accuracy, even though proteins performed better (R² = 0.59 ± 0.02 for proteins vs. R² = 0.42 ± 0.03 for small RNAs; Supplementary Table 4, Figure 4A, 4D, 4E). Given that our Small-seq covered many classes of small RNAs that may behave differently in the context of aging, we next evaluated these classes separately. Interestingly, different small RNA classes showed very distinct age-predictive capabilities. Ribosomal RNAs (rRNAs), small nuclear RNAs (snRNAs), and small nucleolar RNAs (snoRNAs) had little to no association with age (R² < 0.10, Supplementary Table 4, Figure 4B). It should be noted, though, that these are the three RNA classes with the fewest members that we tested, leaving the possibility that some predictive small RNAs in these classes exist but that we were unable to detect or annotate them. tRNAs and tRFs showed a weak association with age (R² = 0.22 ± 0.03 and 0.28 ± 0.03). Finally, only miRNAs showed a moderate association with age (R² = 0.45 ± 0.02). Thus, we conclude that miRNAs are the most age-predictive small RNA class, consistent with the prominence of miRNAs among age-associated small RNAs observed above (Figure 3A). Since the proteins we used for age predictions had been pre-selected for being age-associated (by HRM-MS), we then performed a similar feature selection approach for small RNAs. We focused on miRNAs, the best-performing small RNA class, and among these, we used a set of 20 miRNAs (hereafter named “top20_miRNAs”) that were found by a previous study to have a high association with age ([42], see Methods). Strikingly, using only these 20 miRNAs substantially improved predictions compared to using all small RNAs or all miRNAs (R² = 0.54 ± 0.02, R² = 0.42 ± 0.03 and R² = 0.45 ± 0.02, respectively; Supplementary Table 4, Figure 4B), even though these top20_miRNAs were still slightly less age-predictive than our protein markers.

We noticed that all of our age predictions showed their best accuracy when using only a small, limited set of features. For example, using 21 out of the 31 measured proteins, 38 out of the 608 measured small RNAs, or 6 out of all the 288 measured miRNAs (Supplementary Table 4). The addition of more features from the same dataset (the same type of molecules) would not further improve predictions but rather worsen them, presumably by adding noise (Figure 4G). Therefore, we tested whether adding data from different molecular types could help improve the predictions. To test this hypothesis, we combined our proteomics data with the most predictive small RNA sets (R^2^ > 0.4). We observed improvements for all combinations, with the best performance achieved by the inclusion of all miRNAs or the top20_miRNAs (R² = 0.70 ± 0.02 or R² = 0.73 ± 0.01, respectively, compared to R² = 0.59 ± 0.02 for proteins alone; Figure 4C, 4F, 4G). We then wondered how these improvements occurred. Interestingly, we found that individuals’ delta ages (prediction errors) correlated highly (between 0.7 and 0.95, Figure 4H) among all the different small RNA-based models with age predictive capacity (R^2^ > 0.2), while they correlated only moderately (between 0.25 and 0.44) with individuals’ delta ages of the proteomics-based model (Supplementary Table 5). In other words, for each individual in the cohort, the small RNA-based models all have similar age predictions, while the predictions made by the protein-based model can be quite distinct.

Next, given that our models were trained on only 21 age-associated proteins, we wanted to exclude the possibility that the improved performance of models combining proteomics and small RNA data was simply due to this limited number of variables. We tested this by using the same cross-validation strategy as above, but this time using the untargeted (HRM-MS) instead of the targeted (MRM-MS) proteomics results to build our models. The results showed a higher mean performance of models combining proteomic and miRNA data compared to either data type alone (Supplementary Figure 6), suggesting that proteomic and miRNA data are complementary, even when the number of age-associated proteins is not limiting. Taken together, our observations suggest that models built from proteins or from small RNAs capture different aspects of aging, and therefore, age predictions benefit from their combination.

Finally, we checked the standardized coefficients of our best-performing age-predictive model derived from proteins and top20_miRNAs (Figure 4I, Supplementary Table 6). Interestingly, the three miRNAs with the lowest coefficients were miR-26b-5p, miR-93-5p and miR-26a-5p, all of which have been reported as tumor suppressors [46–48]. This result is consistent with the enrichment in cancer-promoting genes among targets of miRNAs that decrease with age (Figure 3D). The three proteins with the highest coefficients were hemopexin, fibulin-1, and cystatin C. The heme-binding glycoprotein hemopexin plays a key role in protecting LDL [49] and neurons [50] from oxidative stress and is enriched in amyloid deposits in the brains of Alzheimer’s Disease (AD) patients [51]. The calcium-binding glycoprotein fibulin-1 has been previously shown to increase with age and to be associated with diabetes, impaired kidney function, and hemodynamic cardiovascular risk markers [52]. The cysteine protease inhibitor cystatin C has been described previously as increasing with age and being involved in various neurodegenerative diseases, including AD [53, 54]. Finally, two complement system proteins (FIBB and CO5) contributed positive coefficients to the model, consistent with the enrichment of this pathway among proteins up-regulated with age in our HRM-MS and MRM-MS data (Figure 2D). In summary, combining protein and small RNA data allowed us to capture a broader and complementary spectrum of molecules involved in age-related physiological processes, most notably cancer-protective miRNAs and proteins involved in AD and the complement system.

## DISCUSSION

Accurate age predictions from comprehensive molecular data hold great promise for various applications in medicine, forensics, anthropology, or ecology. Our study measured two types of molecular data (proteins and small RNAs) in an easily accessible tissue (blood) to first identify age-associated molecules and second shed light on their suitability, alone or in combination, for age predictions. In the following, we will discuss the individual stages of our study and the insights that were gained.

In the first stage, our untargeted mass spectrometry measurements identified 145 candidate proteins that changed in abundance with age (Figure 2, Supplementary Figure 1, Supplementary Table 1), many of which had not been reported before. Among the upregulated proteins, we observed a significant enrichment in the innate immunity-related complement pathway proteins. One of these newly discovered age-associated proteins is C5 (Complement component 5). Interestingly, C5 deficiencies have been found to be associated with rheumatoid arthritis [55, 56], which is a common age-associated disease. 31 of the 145 candidates were eventually measured by targeted proteomics on the entire cohort. Hereby, ten complement proteins were confirmed as age-increasing, further supporting the association of this pathway with age. This result is in agreement with previous work where the authors found the term “complement coagulation cascades” (KEGG id: hsa04610) to be the second most enriched pathway among the age-associated proteins they detected in a large-scale proteomics assay [18]. Notably, the increase in complement components correlates well with the age-dependent increase in systemic inflammation that is considered a hallmark of aging in humans [3]. Furthermore, the complement system is associated with respiratory failure in COVID-19 patients [57], suggesting that an increased abundance of its components may contribute to the increased impact of this disease on the elderly. Another observation from the targeted proteomics was that three of the six proteins downregulated with age were apolipoproteins. This was remarkable given that only 2.45% of all the 612 tested proteins were apolipoproteins. All these 3 proteins contribute to High-Density Lipoproteins (HDLs) [58], whose abundance is known to decrease with age [59], which are maintained at higher levels in long-lived individuals [59, 60], and whose abundance anti-correlates with coronary heart disease risk [61].

Additionally, we measured small RNAs using the Small-seq methodology and identified 315 small RNAs that changed in abundance with age in human blood plasma (Figure 3, Supplementary Table 3). miRNAs were clearly the most prominent age-associated class of small RNAs, showing a strong down-regulation with age. The age-related decrease in miRNAs is remarkable since this small RNA class has recognized roles in intercellular communication [62, 63]. The loss of these circulating miRNAs may, therefore, contribute to the deregulation of gene expression in various tissues with age [64]. Studies in multiple species have reported a downregulation of Dicer expression with age [65, 66], which may contribute to this phenomenon. Furthermore, the identified age-associated miRNAs themselves are interesting: First, the miRNAs with the highest age-association were actually pairs of closely related miRNAs (i.e., mir-26a-5p and mir-26b-5p as well as mir-374a-5p and mir-374b-5p). While the miRNAs of each pair likely have similar physiological roles, they are transcribed from different chromosomal loci and hence experience synchronous regulation, further strengthening the notion that their decrease with age is no coincidence but the outcome of a concerted regulatory event. Second, the predicted targets of the miRNAs declining with age were enriched for functions promoting growth, cancer, and senescence. Thus, the decrease in these circulating miRNAs could contribute to the increased occurrence of cancers in the elderly by upregulating oncogenes. Third, the very few miRNAs that were upregulated with age had predicted targets involved in insulin resistance and longevity regulation and thus may be important players regulating age-related morbidities or even aging directly [c67].

In the second stage of our study, we used our protein and small RNA measurements to build age-predictive models and compare their accuracy. We were able to build a decent age-predictive model from proteins (R² = 0.59 ± 0.02). Using the small RNAs, we found that miRNAs were by far the most predictive class of small RNAs, while tRFs and tRNAs had little predictive ability, and rRNAs, snoRNAs, and snRNAs had no predictive ability (Figure 4B). The relatively high predictive ability of miRNAs (up to R² = 0.54 ± 0.02 for top20_miRNAs) is consistent with previous reports showing that many miRNAs change with age and are associated with age-dependent diseases [68–70]. However, to the best of our knowledge, no study before has compared the suitability of different classes of small RNAs for age prediction. Our observations should, therefore, help future studies that want to use small RNA biomarkers of aging in blood plasma by instructing them to focus on miRNAs.

We tried to further improve age predictions by combining protein and small RNA data. This worked well, with the best results being obtained from a combination of our age-associated proteins and the top20_miRNAs. Ultimately, our best model used only 15 of the 31 proteins and 9 of the top20_miRNAs and had a performance (R² = 0.73 ± 0.01) that was better than models built from individual molecular data types alone (R² = 0.59 ± 0.02 for proteins, R² = 0.54 ± 0.02 for top20_miRNAs). We wondered what could drive this increase in predictive performance. An indication came from the correlations of delta ages (prediction errors) for all the individuals in our cohort, where all small RNA-based models were highly correlated but showed only little correlation with our protein-based model. This argued that proteins and small RNAs may capture different aspects of age-related human physiology. These results are in line with the study of Huan et al. [24], in which the authors found little correlation between their miRNA-based clock and their mRNA- and DNAm-based clocks and showed that models built from miRNAs in combination with either mRNA or DNAm data had higher performances.

Next, we examined the standardized coefficients of our best-performing age-predictive model to identify the best predictors of age in our dataset. We found that the two lowest coefficients (miR-26b-5p and miR-93-5p) were both miRNAs, while the three highest coefficients were all proteins (CYTC, FBLN1, and HEMO). Consistently, miR-26b-5p and miR-93-5p were significantly more down-regulated with age (FDR <= 1.2 × 10^−7^) than the most significantly down-regulated protein (APOC1, 8 × 10^−4^), while CYTC, FBLN1, and HEMO were significantly more up-regulated (FDR <= 5.5 × 10^−6^) than the most significantly up-regulated small RNA (miR-10b-5p, E = 2.4 × 10^−5^). From a technical standpoint, this suggests that by integrating different molecular data-types, we were able to expand the set of robust features, which improved the capture of subtle aging trends. At a biological level, we also observed distinct predicted functions for the top two negative and the top three positive coefficients. The former, both being miRNAs, are recognized tumor suppressors, while the latter, all three being proteins, are associated with age-related diseases such as Alzheimer’s disease (HEMO and CYTC), kidney disease, and cardiovascular disease (FBLN).

One limitation of our study is that we had to exclude all non-annotated small RNAs from the small RNA data to avoid studying degradation products of larger RNAs. This filtering step removed the majority of the measured small RNAs (88% of the 5,180 measured). It is conceivable that some of the removed RNAs were valid small RNAs with good age-predictive potential, but incomplete annotations in the existing databases prevented their inclusion. Future improvements in genome annotation may address this issue. Additionally, the cohort utilized in our study had a limited size (103 individuals) and limited representation of the global population (all individuals were healthy Northern Americans, and males were overrepresented). Finally, we lacked a replication cohort.

A strength of our study is the two-stage approach for biomarker selection and quantification, where age-associated proteins were pre-selected by HRM-MS and then measured by MRM-MS, while small RNAs measured by Small-seq were further filtered for the set of significantly age-associated miRNAs from the Freedman et al. study [42]. This robust approach gives us more confidence in the validity and effects of these biomarkers. Furthermore, our study is one of the few that measured biomarkers of different molecular types in the same cohort and built age-predictive models from these measurements. And to the best of our knowledge, a combination of proteomics and small RNA measurements for building age-predictive models has never been used before. Finally, only a few studies have measured total small RNAs in plasma with age, and none have compared the capacities of different small RNA classes for age prediction.

Taken together, our study revealed that both proteins and small RNAs in the blood contain highly significant age-associated molecules. Interestingly, we found that most miRNAs showed a strong decrease with age and targeted tumor-suppressor molecules, while most proteins showed a strong increase with age and were enriched in molecules of the adaptive immune system. While proteins and small RNA sets can be used individually to predict human age, we found that their combination improves age predictions. These results suggest that miRNAs provide a good complement to proteomic data for age predictions and that even small sets of highly age-associated miRNAs and proteins could yield highly predictive models. This could be particularly valuable for the development of cost-effective age-predictive strategies that aim to measure only a minimal number of biomarkers. Further studies using larger sample sizes and a replication cohort are still needed to confirm the complementary nature of miRNA and proteomics measurements for age predictions and to define the best miRNAs and proteins to use. However, we see our work as an indication that combining different molecular data types could be a general strategy to improve future aging clocks.

## MATERIALS AND METHODS

### Study population

103 plasma samples from disease-free and generally healthy individuals were acquired from Precision Med Inc. The cohort includes 12 females and 91 males. All participants provided written informed consent.

### Untargeted proteomics profiling

Plasma samples from 19 young male individuals (<31 years old) and 25 old male individuals (>49 years old) were selected for Hyper-reaction monitoring-MS (HRM-MS) proteomics profiling, which was performed by Biognosys AG (Switzerland). All used solvents were HPLC-grade from Sigma-Aldrich unless otherwise stated.

#### Sample preparation and library characterization

10 μl of each plasma sample was reduced using Biognosys’ Reduction Solution for 1 h at 37°C and alkylated using Biognosys’ Alkylation Solution for 30 min at room temperature in the dark. Subsequently, digestion of approximately 100 μg of protein per sample was carried out using trypsin (Promega) overnight at 37°C at a protein:protease ratio of 50:1. Peptides were desalted using a C18 MicroSpin plate (The Nest Group) according to the manufacturer’s instructions and dried down using a SpeedVac system. Peptides were resuspended in 40 μl LC solvent A (1% acetonitrile, 0.1% formic acid (FA)) spiked with Biognosys’ HRM kit calibration. For HPRP fractionation, equal sample volumes were pooled according to sample group (young and elderly). The two pools were each diluted 4-fold in 0.2 M ammonium formate (pH 10) and applied to a C18 MicroSpin column (The Nest Group). The peptides were then eluted with buffers containing 0.05 M ammonium formate and increasing acetonitrile concentrations (5, 10, 12, 14, 16, 18, 20, 22, 24, 26, 30 and 70% for “young” samples and 14, 16, 18, 20, 22, 24, 26, 30 and 70% for “elderly” samples). Note that Biognosys’ standard procedure collects six fractions. Here, more fractions were generated to obtain a deeper library. The eluates were dried down, resolved in 17 μl solvent A, and spiked with Biognosys’ HRM kit calibration peptides prior to mass spectrometric analyses. The final peptide concentrations in all samples and fractions were determined using a UV/VIS Spectrometer (SpectroSTAR nano, BMG Biotech).

#### LC-MS/MS shotgun measurements

2 μg of peptides (with the exception of only 1.5 μg for fraction 5% of the “young” pool) were injected into an in-house packed C18 column (Dr. Maisch ReproSil Pur, 1.9 μm particle size, 120 Å pore size; 75 μm inner diameter, 50 cm length, New Objective) on a Thermo Scientific Easy nLC 1200 nano-liquid chromatography system connected to a Thermo Scientific Q Exactive HF mass spectrometer equipped with a standard nano-electrospray source. LC solvents were A: 1% acetonitrile in water with 0.1% formic acid; B: 15% water in acetonitrile with 0.1% formic acid. The nonlinear LC gradient was 1–52% solvent B in 60 minutes, followed by 52–90% B in 0.1 minutes, and 90% B for 10 minutes. A modified TOP15 method was used [71]. The mass spectrometric data were analyzed using MaxQuant 1.5.6.5 software [72], with the false discovery rate on peptide and protein levels set to 1%. A human UniProt. fasta database (Homo sapiens, 2015-08-28) was used, allowing for 2 missed cleavages and variable modifications (N-term acetylation, methionine oxidation, lysine/arginine carbamylation, asparagine/gluta-mine deamidation).

#### LC-MS/MS HRM measurements

2 μg of peptides per sample were injected into an in-house packed C18 column (Dr. Maisch ReproSil Pur, 1.9 μm particle size, 120 Å pore size; 75 μm inner diameter, 50 cm length, New Objective) on a Thermo Scientific Easy nLC 1200 nano-liquid chromatography system connected to a Thermo Scientific Q Exactive HF mass spectrometer. LC solvents were A: 1% acetonitrile in water with 0.1% formic acid; B: 15% water in acetonitrile with 0.1% formic acid. The nonlinear LC gradient was 1–52% solvent B in 60 minutes, followed by 52–90% B in 10 seconds, and 90% B for 10 minutes. A DIA method with one full-range survey scan and 14 DIA windows was used. HRM mass spectrometric data were analyzed using Spectronaut 10 software (Biognosys). The false discovery rate on peptide levels was set to 1%, data were filtered using row-based extraction. The assay library (protein inventory) generated in this project was used for analysis. The HRM measurements analyzed with Spectronaut were normalized using local regression normalization [73].

#### Preprocessing and statistics

Protein intensities measurements were log2 transformed. The significance of differential expression between the two age groups was assessed with the Mann-Whitney test. In order to account for multiple testing, False Discovery Rate adjusted *p*-values (*q*-values) were computed using the *q* value package (version 2.26.0) [74]. 145 out of 612 proteins had *q*-values below 0.2 and were considered significant.

### Targeted proteomics profiling

#### LC-MS/MS MRM measurements

Targeted proteomics measuring 125 human plasma proteins was performed using an Agilent 6490 triple quadrupole mass spectrometer (Agilent Technologies) and a commercially available PeptiQuant™ Plus Proteomics Kit (MRM Proteomics Inc.), according to the manufacturer’s instructions. This kit was chosen because it measures 44 out of the 145 significant proteins from our first proteomics screen. Briefly, plasma proteins were denatured, reduced, alkylated, and digested with trypsin. One proteotypic peptide per protein was used as a surrogate marker for determining plasma protein concentrations. Each batch was comprised of 50 experimental samples, 9 quality control samples, and 8 calibration samples. A constant concentration of a stable isotope-labeled standard (SIS) peptide was added to each sample, which was used for normalization. The calibration curve spanned a 1000-fold concentration range with 8 different calibration points. The quality controls and standard curve peptides were spiked into a digested bovine serum albumin surrogate matrix to avoid the problems associated with the presence of the endogenous analytes in normal human plasma. The quantification of protein concentration from raw MRM data was done using the software Skyline (version 3.7) [75].

#### Preprocessing and statistics

Proteins with too low measurements were removed from all analyses. Proteins were kept if they had more than 50% of samples with values higher than the lowest calibration threshold measurement in all 3 batches. This resulted in 77 proteins being kept, out of which 31 were significantly associated with age in our untargeted proteomics screen. Measurement values below zero were set to zero, and one count was added to each value before log2 transformation. All analyses were made on the 31 proteins selected from untargeted proteomics. Batch correction between experimental runs was done using parametric empirical Bayes (via the ComBat function from the sva package (version 3.42.0) [76]) and chronological age as the outcome of interest. Significant proteins were determined by linear modeling, using protein levels as the dependent variable and chronological age as the explanatory variable. Correction for multiple testing was done using the Benjamini-Hochberg False Discovery Rate (BH FDR) adjustment method with the R function p.adjust [77]. A total of 21 out of 31 proteins were significant at an FDR of 0.2.

### Small RNA expression profiling

#### Small RNA measurements

Total RNA was extracted from plasma samples using the Qiagen miRNeasy Serum/Plasma Kit. Briefly, frozen plasma samples were thawed, vortexed, and centrifuged for 10 min at 16,000 g. 50 μl of the supernatant were used for RNA extraction following the manufacturer’s instructions. Purified RNA was eluted in 50 μl water and stored at −80°C. 4 μl of purified RNA was used in a Small-seq protocol to construct sequencing libraries [1]. Small RNA libraries were pooled and sequenced using an Illumina HiSeq 3000 platform for 100-bp single-read. Reads were mapped to 202,272 Ensembl transcript IDs using the Small-seq data analysis pipeline [1] and to 4,121 tRFs using the MINTmap pipeline [2] on the human genome version hg38. Our study uses the annotations “tRF” and “tRNA”. Both refer to fragments of tRNAs detected by the Small-seq pipeline. The difference is that “tRNA” refers to any RNA fragment that, according to Ensembl, maps to a tRNA, while “tRF” refers to only a specific subset of tRNA fragments that are not a result of random fragmentation but are enzymatically generated and have dedicated physiological roles [78].

#### Preprocessing and statistics

Only small RNAs with read counts higher than 1 in more than 20% of the samples were kept, resulting in a list of 5,180 small RNAs. Counts were rounded, normalized, and differential expression analysis was performed using the DESeq2 package (version 1.34.0) [79]. Briefly, DESeq2 analysis consists of counts normalization by size factors, estimation of RNA mean and dispersion, fitting a negative binomial Generalized Linear Model, assessing significance with the Wald test, and correcting for multiple testing by BH FDR adjustment. Small RNA levels were used as the dependent variable and chronological age as the explanatory variable. A local fit was used for estimating dispersions. Size factor-normalized small RNA counts were obtained using the ‘counts’ function with the argument “normalized = TRUE”. After DESeq2 analysis, transcript biotypes were defined for all identified small RNAs using the biomaRt package (version 2.50.0) [80]. Small RNAs with unclear transcript biotypes were removed from the analyses since they could be degradation products (i.e., of protein-coding mRNAs, lncRNAs, etc.,). A total of 608 small RNAs were kept after this filtering step, of which 315 were significantly associated with age at an FDR threshold of 0.2.

### Data analysis

All data analyses were carried out in R (version 4.1.2) [77], with the packages tidyverse (version 1.3.1) [81], Biobase (version 2.54.0) [82], data.table (version 1.14.2) [83] and knitr (version 1.36) [84].

### Predictions of miRNA targets

miRNAs can have many predicted targets. To avoid dealing with an excessive number of targets, we decided to pre-filter the list of age-associated miRNAs by keeping only the 7 up-regulated miRNAs with an FDR below 0.2 and the 110 down-regulated miRNAs with an FDR below 0.001. miRNA targets were predicted using the multiMiR package (version 1.16.0, database version 2.3.0) [44]. multiMiR allows users to query 3 experimental and 7 miRNA target prediction databases. To keep only the most confidently predicted targets, we used the following filters: Targets were selected only if they were found in 2 out of the 3 experimental databases. Furthermore, they had to be found in 4 out of the 7 prediction databases, and to rank in the top 10% of predicted targets for a given miRNA. After this filtering, the up-regulated miRNAs yielded 22 predicted targets, and the down-regulated miRNAs yielded 2159 predicted targets.

### Pathway enrichment analysis

KEGG pathway enrichment analysis was performed using gprofiler2 (version 0.2.1) and *p*-values were corrected using the gSCS correction method [85]. A custom background for the computation of significance was used using the argument domain_scope = “custom”. For unbiased proteomics, the background used was the set of all 612 measured proteins. For miRNA targets, the background used was the set of all 26,193 human mRNA targets present in the multiMiR database, which was fetched using the ‘list_multimir’ command.

### Creation of age predictive models

To conduct feature selection of small RNAs in an unbiased way, we chose those found to be differentially expressed with age by another study [42]. After matching the transcript IDs in common with our study, we selected the top 20 most significantly age-associated RNAs, all of which were miRNAs, and named this group “top20_miRNAs”.

For modeling, we used LASSO regression via the glmnet package (version 4.1-3) [86, 87]. Local regression normalized data were used for the untargeted proteomics, batch corrected data were used for the targeted proteomics, and DESeq2-normalized counts were used for the small RNAs (see the corresponding Preprocessing and statistics sections for details). All datasets were in raw scale (not log-transformed). The loss function used was Mean Absolute Error. For estimation of model performance and tuning of the lambda hyperparameter (which is the strength of regularization), we performed a 10-fold cross-validation scheme with 100 repeats. Using many repeats allowed us to obtain more stable estimates despite a modest sample size. A final model was fitted by using the lambda with the lowest mean cross-validation error across all 100 repeats. Lastly, we obtained standardized model coefficients by multiplying the raw coefficients provided by the model with their corresponding variable standard deviation, as described previously [88].

### Data availability statement

The mass spectrometry-based proteomic data are openly available through the Proteomics Identification Database [89] under project PXD028281 for the HRM-MS data and PXD028295 for the MRM-MS data. The Small-seq data are openly available at NCBI’s Gene Expression Omnibus [90] under accession number GSE182598.

## Supplementary Materials

Supplementary Figures

Supplementary Tables 1, 3 and 6

Supplementary Tables 2 and 4-5
