# Supplementary material for: Age prediction from human blood plasma using proteomic and small RNA data: a comparative analysis
Source: Aging (Albany NY). 2023 Jun 20;15(12):5240–65. doi: 10.18632/aging.204787 (PMC10333066; doi:10.18632/aging.204787)
Supplement: Supplementary Figures [file aging-15-204787-s001.pdf]

SUPPLEMENTARY FIGURES

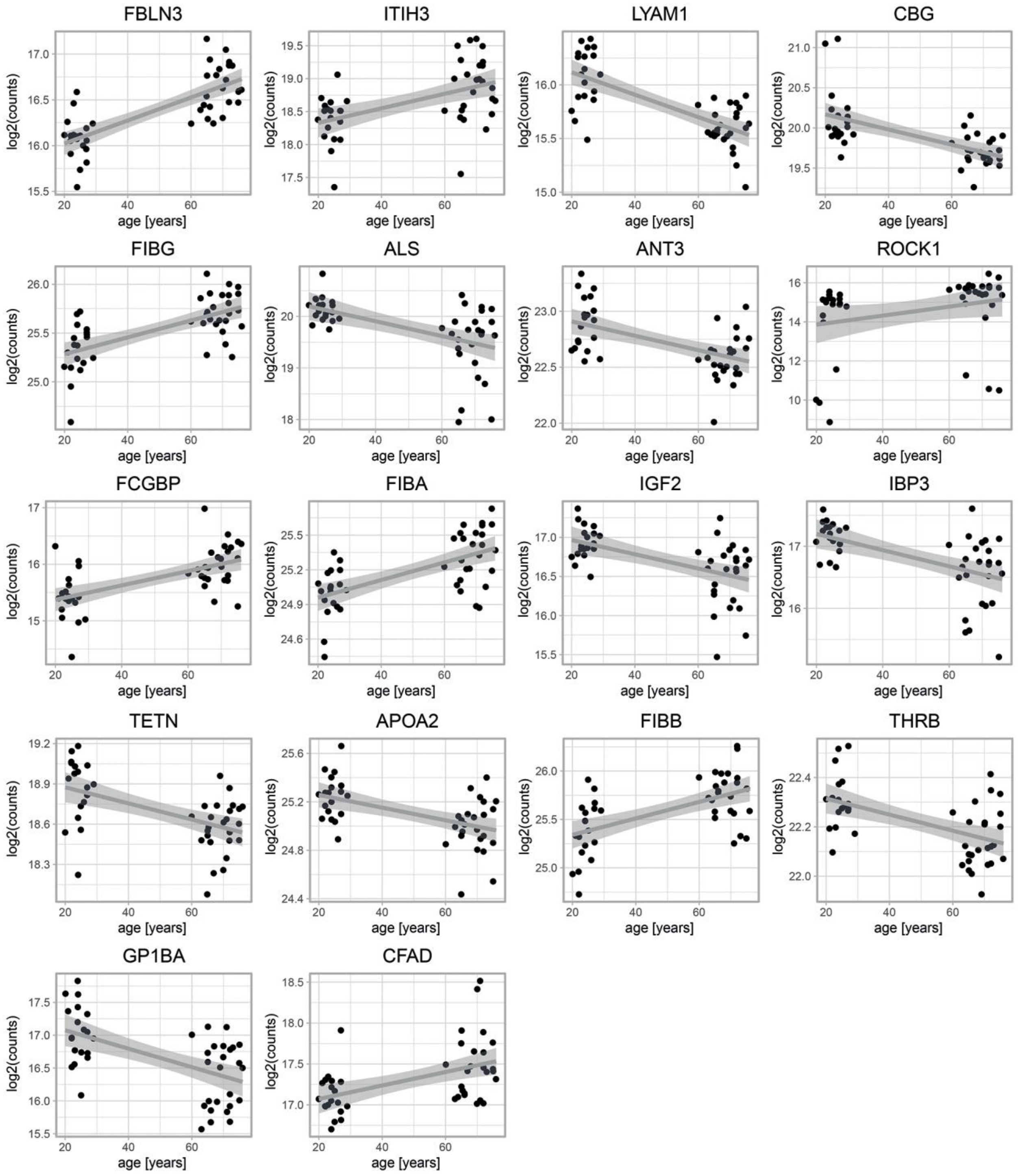

Supplementary Figure 1. Scatter plots of the most age-associated proteins from untargeted proteomics. Scatter plots of age vs. protein abundance for 22 out of the 24 most significantly age-associated proteins from the HRM-MS. Scatter plots for the two proteins not shown here can be found in Figure 2E. Black line and shadow: linear regression and 95% confidence interval, respectively.

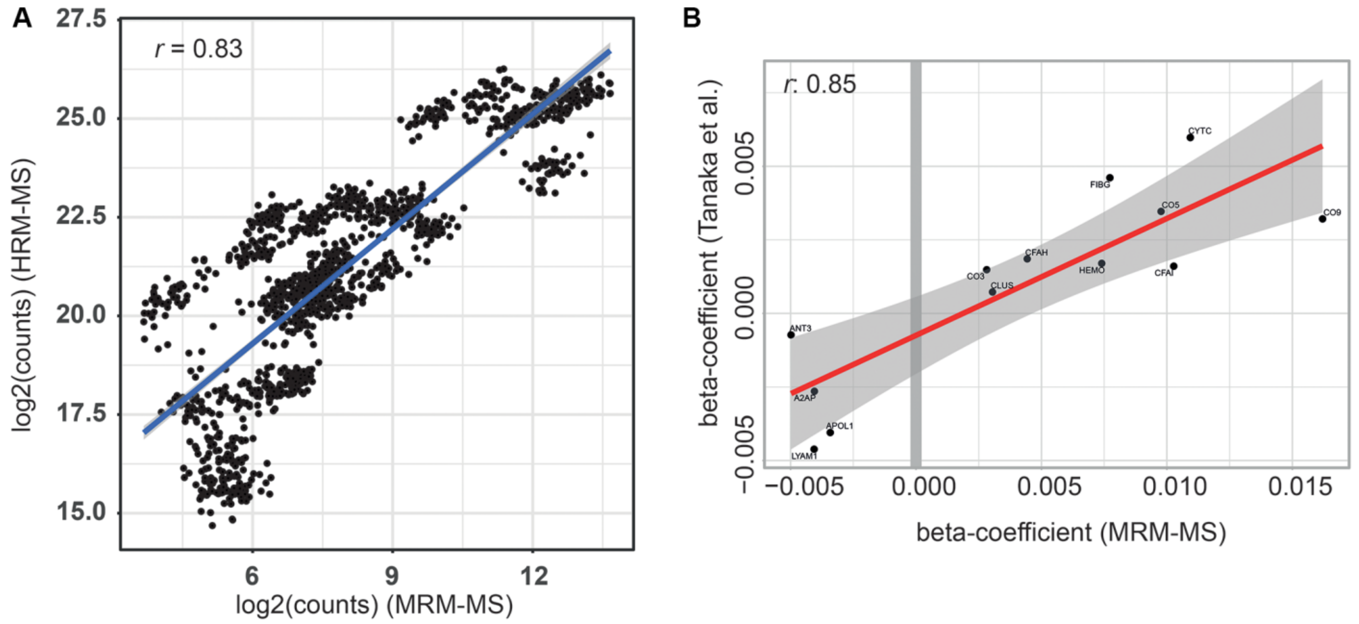

Supplementary Figure 2. Comparison of the proteomics from this study with previous work. (**A**) Correlation between log2 counts of the two MS experiments across proteins and samples. Blue line and shadow: linear regression and 95% confidence interval, respectively. (**B**) Comparisons of age-dependent protein abundance changes in our study and in [18]. The correlations of the beta coefficients reported in [18] are plotted on the y-axis and the beta coefficients from our MRM-MS are plotted on the x-axis. Red line and shadow: linear regression and 95% confidence interval, respectively.

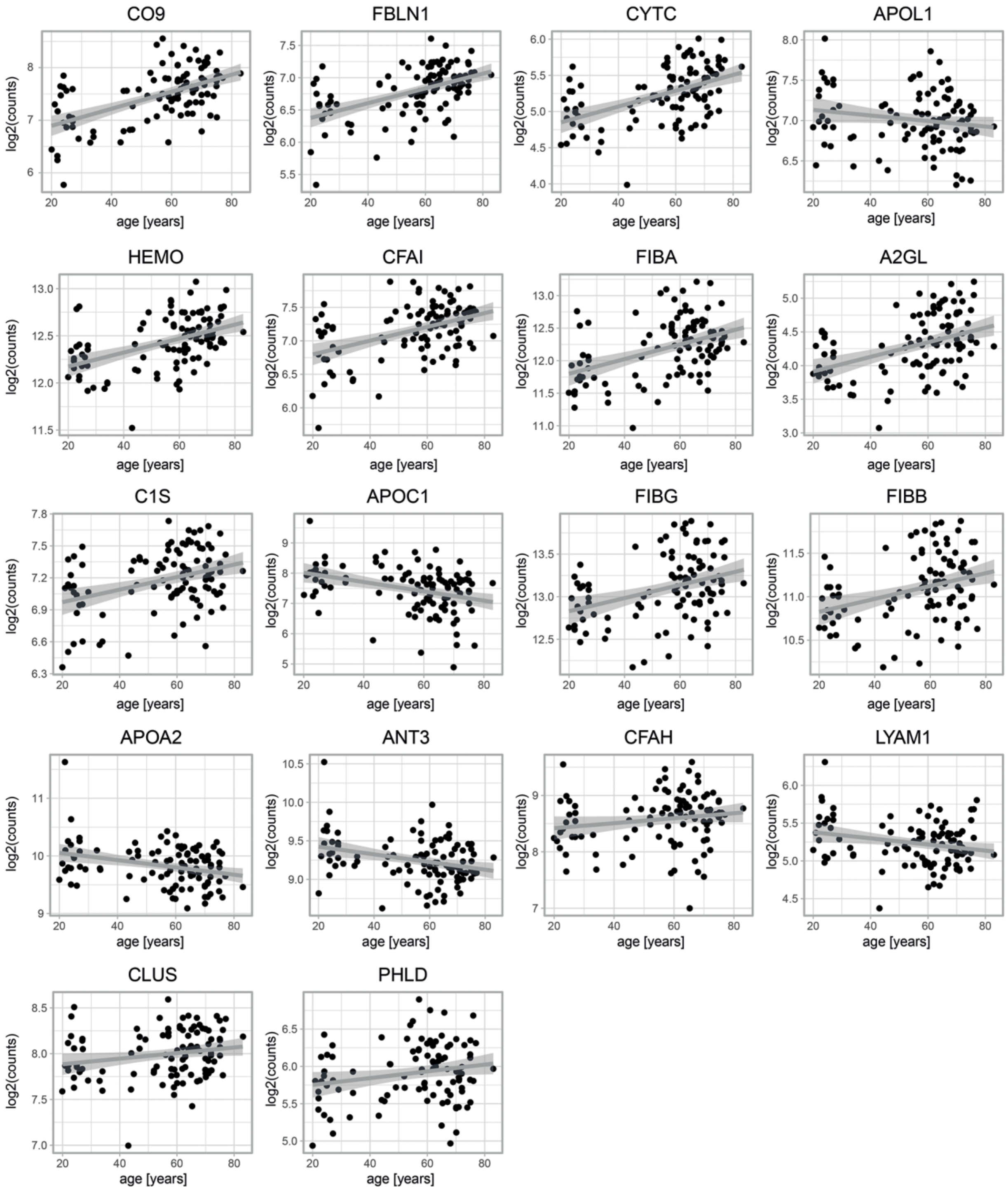

Supplementary Figure 3. Scatter plots of the most age-associated proteins from targeted proteomics. Scatter plots of age vs. protein abundance for 22 out of the 24 most significantly age-associated proteins from the MRM-MS. Scatter plots for the two proteins not shown here can be found in Figure 2E. Black line and shadow: Linear regression and 95% confidence interval, respectively.

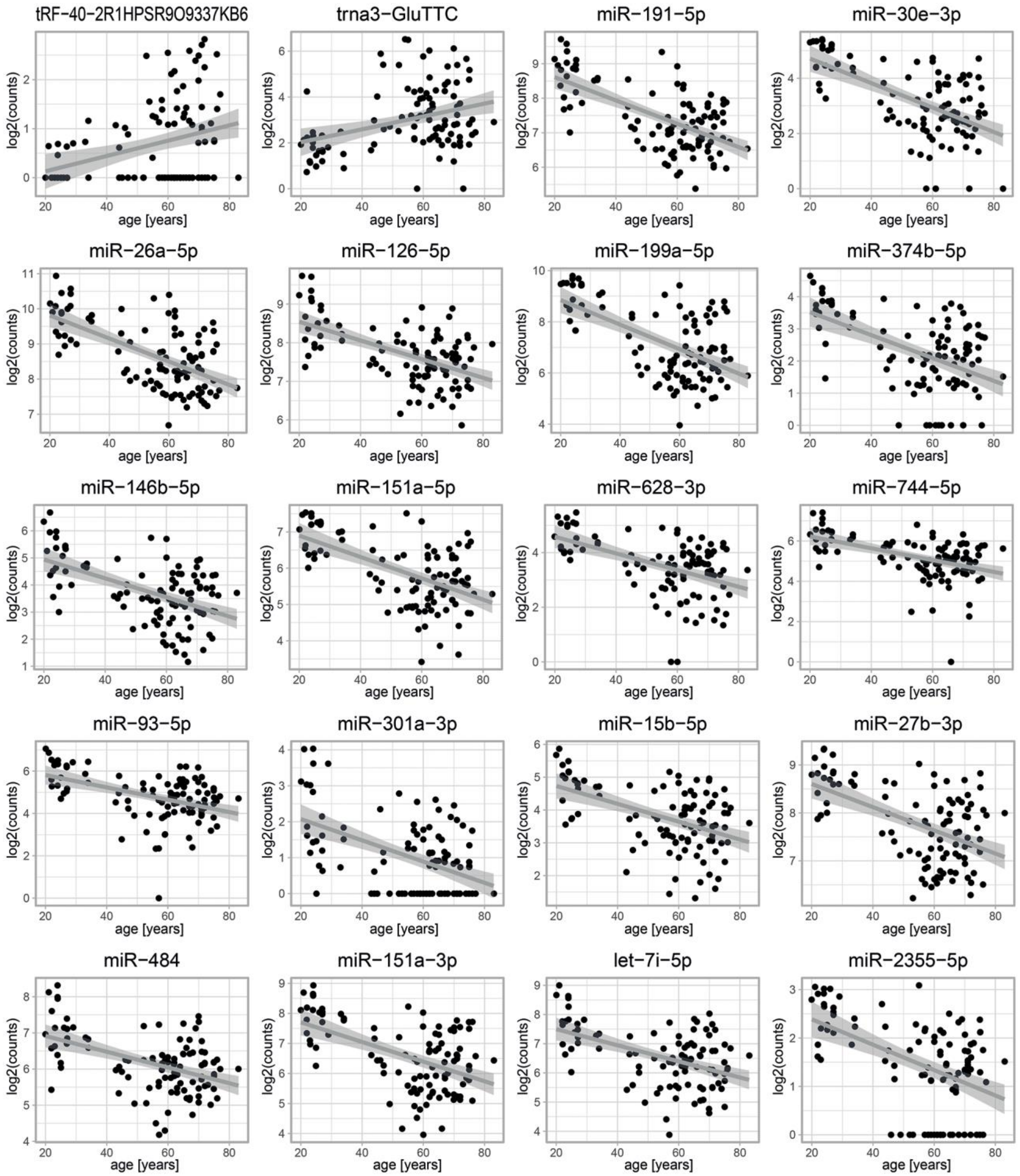

Supplementary Figure 4. Scatter plots of the most age-associated small RNAs. Scatter plots of age vs. protein abundance for 20 out of the 24 most significantly age-associated small RNAs. Scatter plots for the four small RNAs not shown here can be found in Figure 3B. Black line and shadow: Linear regression and 95% confidence interval, respectively.

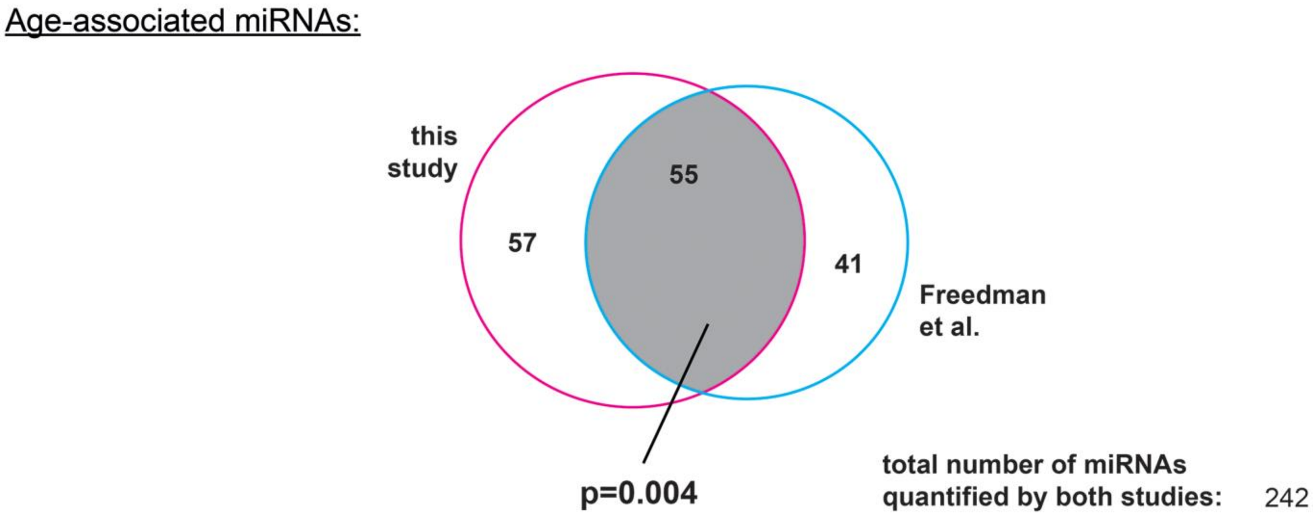

Supplementary Figure 5. Comparison of the age-associated miRNAs from this study with previous work. Overlap between miRNAs found to be the most significantly age-associated in our study (FDR < 0.001, 96 miRNAs) and the study of Freedman et al. (FDR < 0.05, 112 miRNAs), out of 242 miRNAs measured in both studies.

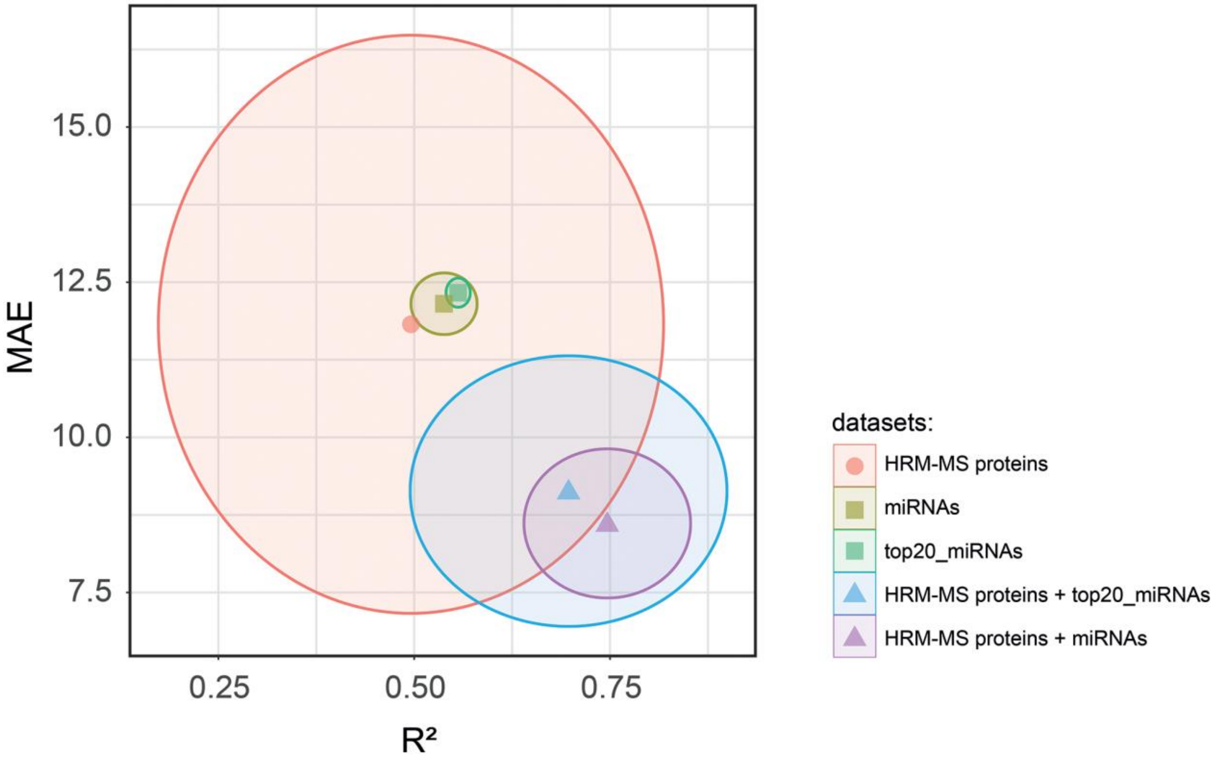

Supplementary Figure 6. Performance of age-predictive models built from untargeted proteomic and miRNA data. Performance of the models built from untargeted proteomic (HRM-MS) and miRNA data. The mean (dot) and standard deviation (circle) of two error metrics are shown: the coefficient of determination (R^2) on the x-axis and the Mean Absolute Error (MAE) on the y-axis.
