# Supplementary material for: Age prediction from human blood plasma using proteomic and small RNA data: a comparative analysis
Source: Aging (Albany NY). 2023 Jun 20;15(12):5240–65. doi: 10.18632/aging.204787 (PMC10333066; doi:10.18632/aging.204787)
Supplement: Supplementary Tables 1, 3 and 6 [file aging-15-204787-s002.docx]

# **Supplementary Table 1. Results of the HRM-MS.**

| **uniprot_id** | **protein_name** | **gene_name** | **protein_description** | **Young** | **Old** | **L2FC** | **FDR** | **minusLog10_FDR** | **signif** | **is_new** |
| --- | --- | --- | --- | --- | --- | --- | --- | --- | --- | --- |
| Q12805 | FBLN3 | EFEMP1 | EGF-containing fibulin-like extracellular matrix protein 1 | 16.094 | 16.6641 | 0.57 | 0.0001 | 3.9658 | TRUE | new |
| P08697 | A2AP | SERPINF2 | Alpha-2-antiplasmin | 21.7784 | 21.5298 | -0.2485 | 0.0005 | 3.2948 | TRUE | L.T |
| P14151 | LYAM1 | SELL | L-selectin | 16.1012 | 15.6129 | -0.4883 | 0.0005 | 3.2948 | TRUE | L.T |
| P08185 | CBG | SERPINA6 | Corticosteroid-binding globulin | 20.1784 | 19.7191 | -0.4593 | 0.0009 | 3.0304 | TRUE | L |
| P02679 | FIBG | FGG | Fibrinogen gamma chain | 25.3452 | 25.7207 | 0.3755 | 0.001 | 2.9852 | TRUE | new |
| P35858 | ALS | IGFALS | Insulin-like growth factor-binding protein complex acid labile subunit | 20.1594 | 19.6138 | -0.5456 | 0.0045 | 2.3496 | TRUE | A |
| P01008 | ANT3 | SERPINC1 | Antithrombin-III | 22.9136 | 22.5982 | -0.3154 | 0.0045 | 2.3496 | TRUE | L |
| P01031 | CO5 | C5 | Complement C5 | 20.1491 | 20.3895 | 0.2404 | 0.0045 | 2.3496 | TRUE | new |
| Q9Y6R7 | FCGBP | FCGBP | IgGFc-binding protein | 15.483 | 16.047 | 0.564 | 0.0045 | 2.3496 | TRUE | new |
| P02671 | FIBA | FGA | Fibrinogen alpha chain | 25.0125 | 25.3481 | 0.3355 | 0.0045 | 2.3496 | TRUE | new |
| P01344 | IGF2 | IGF2 | Insulin-like growth factor II | 16.9475 | 16.5672 | -0.3804 | 0.0045 | 2.3496 | TRUE | new |
| P17936 | IBP3 | IGFBP3 | Insulin-like growth factor-binding protein 3 | 17.1663 | 16.6579 | -0.5084 | 0.005 | 2.3041 | TRUE | L |
| P05452 | TETN | CLEC3B | Tetranectin | 18.875 | 18.5869 | -0.2881 | 0.005 | 2.2975 | TRUE | new |
| P02652 | APOA2 | APOA2 | Apolipoprotein A-II | 25.2533 | 25.0095 | -0.2439 | 0.0072 | 2.1446 | TRUE | new |
| P02675 | FIBB | FGB | Fibrinogen beta chain | 25.4075 | 25.7805 | 0.3731 | 0.0072 | 2.1446 | TRUE | new |
| P00734 | THRB | F2 | Prothrombin | 22.3113 | 22.155 | -0.1564 | 0.0072 | 2.1446 | TRUE | L |
| P07359 | GP1BA | GP1BA | Platelet glycoprotein Ib alpha chain | 17.0873 | 16.4607 | -0.6266 | 0.0089 | 2.0525 | TRUE | L |
| P00746 | CFAD | CFD | Complement factor D | 17.1364 | 17.5329 | 0.3965 | 0.0113 | 1.9462 | TRUE | new |
| Q06033 | ITIH3 | ITIH3 | Inter-alpha-trypsin inhibitor heavy chain H3 | 18.4175 | 18.9446 | 0.5271 | 0.0113 | 1.9462 | TRUE | new |
| Q13464 | ROCK1 | ROCK1 | Rho-associated protein kinase 1 | 14.7135 | 15.453 | 0.7395 | 0.0152 | 1.8174 | TRUE | new |
| P10643 | CO7 | C7 | Complement component C7 | 19.3297 | 19.6625 | 0.3328 | 0.0172 | 1.7645 | TRUE | new |
| P11142 | HSP7C | HSPA8 | Heat shock cognate 71 kDa protein | 15.337 | 14.3652 | -0.9718 | 0.0202 | 1.6945 | TRUE | new |
| O00750 | P3C2B | PIK3C2B | Phosphatidylinositol 4-phosphate 3-kinase C2 domain-containing subunit beta | 19.852 | 20.1395 | 0.2875 | 0.0202 | 1.6945 | TRUE | new |
| Q16610 | ECM1 | ECM1 | Extracellular matrix protein 1 | 18.6967 | 18.4207 | -0.276 | 0.0238 | 1.6233 | TRUE | new |
| P18428 | LBP | LBP | Lipopolysaccharide-binding protein | 17.6698 | 18.0768 | 0.407 | 0.0238 | 1.6233 | TRUE | new |
| P06396 | GELS | GSN | Gelsolin | 21.2844 | 21.0085 | -0.2759 | 0.0248 | 1.605 | TRUE | new |
| Q5SW79 | CE170 | CEP170 | Centrosomal protein of 170 kDa | 21.3318 | 20.7833 | -0.5485 | 0.0259 | 1.5863 | TRUE | new |
| P50552 | VASP | VASP | Vasodilator-stimulated phosphoprotein | 16.4761 | 17.5016 | 1.0255 | 0.0271 | 1.5672 | TRUE | new |
| P0C0L4 | CO4A | C4A | Complement C4-A | 23.4797 | 23.7316 | 0.2519 | 0.0275 | 1.5608 | TRUE | new |
| P00742 | FA10 | F10 | Coagulation factor X | 18.5077 | 18.3387 | -0.169 | 0.0275 | 1.5608 | TRUE | L |
| P51884 | LUM | LUM | Lumican | 18.383 | 18.6558 | 0.2728 | 0.0275 | 1.5608 | TRUE | new |
| Q86VF7 | NRAP | NRAP | Nebulin-related-anchoring protein | 16.2709 | 16.6786 | 0.4077 | 0.0275 | 1.5608 | TRUE | new |
| P27169 | PON1 | PON1 | Serum paraoxonase/arylesterase 1 | 21.2762 | 20.9824 | -0.2938 | 0.0275 | 1.5608 | TRUE | new |
| Q92954 | PRG4 | PRG4 | Proteoglycan 4 | 17.294 | 17.6612 | 0.3672 | 0.0275 | 1.5608 | TRUE | new |
| P37802 | TAGL2 | TAGLN2 | Transgelin-2 | 17.8695 | 17.1253 | -0.7442 | 0.0275 | 1.5608 | TRUE | new |
| P55290 | CAD13 | CDH13 | Cadherin-13 | 11.9379 | 11.5446 | -0.3933 | 0.0289 | 1.5391 | TRUE | new |
| P23528 | COF1 | CFL1 | Cofilin-1 | 16.6839 | 15.8802 | -0.8036 | 0.0296 | 1.5288 | TRUE | new |
| P23142 | FBLN1 | FBLN1 | Fibulin-1 | 18.0347 | 18.3052 | 0.2705 | 0.0296 | 1.5288 | TRUE | new |
| P34096 | RNAS4 | RNASE4 | Ribonuclease 4 | 10.8994 | 11.2833 | 0.3839 | 0.0312 | 1.5065 | TRUE | L |
| P11169 | GTR3;GTR14 | SLC2A3 | Solute carrier family 2, facilitated glucose transporter member 3;Solute carrier family 2, facilitated glucose transporter member 14 | 16.8091 | 17.7854 | 0.9764 | 0.0372 | 1.429 | TRUE | new |
| P01768 | HV307 |  | Ig heavy chain V-III region CAM | 17.1322 | 17.0499 | -0.0823 | 0.0372 | 1.429 | TRUE | new |
| P05156 | CFAI | CFI | Complement factor I | 20.3808 | 20.5164 | 0.1357 | 0.0392 | 1.4068 | TRUE | L |
| P05165 | PCCA | PCCA | Propionyl-CoA carboxylase alpha chain, mitochondrial | 16.4019 | 16.0321 | -0.3698 | 0.0444 | 1.3525 | TRUE | new |
| P63261 | ACTG | ACTG1 | Actin, cytoplasmic 2 | 16.2149 | 15.6003 | -0.6146 | 0.0461 | 1.3366 | TRUE | new |
| Q9HDC9 | APMAP | APMAP | Adipocyte plasma membrane-associated protein | 15.4171 | 15.7802 | 0.3632 | 0.0461 | 1.3366 | TRUE | L |
| P02748 | CO9 | C9 | Complement component C9 | 20.7352 | 21.0696 | 0.3345 | 0.0461 | 1.3366 | TRUE | L |
| Q08380 | LG3BP | LGALS3BP | Galectin-3-binding protein | 16.7581 | 17.5385 | 0.7805 | 0.0461 | 1.3366 | TRUE | new |
| O95810 | SDPR | SDPR | Serum deprivation-response protein | 15.8888 | 15.2844 | -0.6044 | 0.0461 | 1.3366 | TRUE | new |
| Q03135 | CAV1 | CAV1 | Caveolin-1 | 20.8263 | 20.5152 | -0.3111 | 0.0473 | 1.325 | TRUE | new |
| P08603 | CFAH | CFH | Complement factor H | 22.6516 | 22.8156 | 0.164 | 0.0473 | 1.325 | TRUE | new |
| P01034 | CYTC | CST3 | Cystatin-C | 16.5799 | 16.8243 | 0.2444 | 0.0473 | 1.325 | TRUE | new |
| P04406 | G3P | GAPDH | Glyceraldehyde-3-phosphate dehydrogenase | 17.7974 | 17.1417 | -0.6557 | 0.0473 | 1.325 | TRUE | new |
| P04792 | HSPB1 | HSPB1 | Heat shock protein beta-1 | 14.5216 | 13.7922 | -0.7293 | 0.0473 | 1.325 | TRUE | new |
| P00747 | PLMN | PLG | Plasminogen | 22.6138 | 22.4368 | -0.177 | 0.0473 | 1.325 | TRUE | L |
| P19827 | ITIH1 | ITIH1 | Inter-alpha-trypsin inhibitor heavy chain H1 | 22.2988 | 22.1527 | -0.1461 | 0.049 | 1.3097 | TRUE | L |
| P07737 | PROF1 | PFN1 | Profilin-1 | 18.0465 | 17.4299 | -0.6166 | 0.049 | 1.3097 | TRUE | new |
| A6NGH7 | CC160 | CCDC160 | Coiled-coil domain-containing protein 160 | 20.7719 | 22.088 | 1.3161 | 0.0517 | 1.2866 | TRUE | new |
| P02765 | FETUA | AHSG | Alpha-2-HS-glycoprotein | 23.8322 | 23.6099 | -0.2224 | 0.0527 | 1.2782 | TRUE | L.T |
| P01859 | IGHG2 | IGHG2 | Ig gamma-2 chain C region | 26.5383 | 26.9825 | 0.4442 | 0.0527 | 1.2782 | TRUE | new |
| Q86UD1 | OAF | OAF | Out at first protein homolog | 17.068 | 17.445 | 0.377 | 0.0527 | 1.2782 | TRUE | L |
| Q13418 | ILK | ILK | Integrin-linked protein kinase | 15.5419 | 14.6104 | -0.9314 | 0.0556 | 1.255 | TRUE | new |
| P62328 | TYB4 | TMSB4X | Thymosin beta-4 | 18.1843 | 17.545 | -0.6392 | 0.0586 | 1.2319 | TRUE | new |
| P61769 | B2MG | B2M | Beta-2-microglobulin | 17.725 | 18.018 | 0.293 | 0.059 | 1.2292 | TRUE | new |
| Q15485 | FCN2 | FCN2 | Ficolin-2 | 17.5148 | 17.0735 | -0.4413 | 0.059 | 1.2292 | TRUE | L |
| P26927 | HGFL | MST1 | Hepatocyte growth factor-like protein | 16.8866 | 16.5459 | -0.3407 | 0.059 | 1.2292 | TRUE | new |
| P22105 | TENX | TNXB | Tenascin-X | 15.2464 | 14.6515 | -0.5949 | 0.059 | 1.2292 | TRUE | L |
| P43251 | BTD | BTD | Biotinidase | 18.2985 | 17.9541 | -0.3444 | 0.0622 | 1.206 | TRUE | new |
| P01019 | ANGT | AGT | Angiotensinogen | 22.4877 | 22.0256 | -0.4621 | 0.0656 | 1.1829 | TRUE | new |
| P12814 | ACTN1 | ACTN1 | Alpha-actinin-1 | 17.2906 | 16.3296 | -0.9611 | 0.0692 | 1.16 | TRUE | new |
| Q15113 | PCOC1 | PCOLCE | Procollagen C-endopeptidase enhancer 1 | 14.9428 | 14.0103 | -0.9325 | 0.0729 | 1.1372 | TRUE | L |
| P00751 | CFAB | CFB | Complement factor B | 22.2822 | 22.4185 | 0.1363 | 0.0737 | 1.1325 | TRUE | new |
| Q6P387 | CP046 | C16orf46 | Uncharacterized protein C16orf46 | 19.8742 | 19.4685 | -0.4057 | 0.0737 | 1.1325 | TRUE | new |
| Q9ULD2 | MTUS1 | MTUS1 | Microtubule-associated tumor suppressor 1 | 20.8837 | 19.8292 | -1.0545 | 0.0737 | 1.1325 | TRUE | new |
| Q6EMK4 | VASN | VASN | Vasorin | 15.8267 | 15.6802 | -0.1465 | 0.0737 | 1.1325 | TRUE | new |
| Q05D32 | CTSL2 | CTDSPL2 | CTD small phosphatase-like protein 2 | 22.2614 | 22.0202 | -0.2411 | 0.0737 | 1.1323 | TRUE | new |
| P01719 | LV501 |  | Ig lambda chain V-V region DEL | 20.8706 | 20.0568 | -0.8138 | 0.0737 | 1.1323 | TRUE | new |
| P26038 | MOES | MSN | Moesin | 16.5923 | 16.2779 | -0.3144 | 0.0737 | 1.1323 | TRUE | new |
| P67936 | TPM4 | TPM4 | Tropomyosin alpha-4 chain | 17.3422 | 16.6467 | -0.6954 | 0.0737 | 1.1323 | TRUE | new |
| P02774 | VTDB | GC | Vitamin D-binding protein | 24.2801 | 24.1316 | -0.1485 | 0.0737 | 1.1323 | TRUE | new |
| Q8IZ83 | A16A1 | ALDH16A1 | Aldehyde dehydrogenase family 16 member A1 | 19.5898 | 19.4124 | -0.1774 | 0.0777 | 1.1094 | TRUE | new |
| P02654 | APOC1 | APOC1 | Apolipoprotein C-I | 21.6026 | 21.28 | -0.3225 | 0.0909 | 1.0416 | TRUE | new |
| Q14624 | ITIH4 | ITIH4 | Inter-alpha-trypsin inhibitor heavy chain H4 | 22.9465 | 22.8388 | -0.1077 | 0.0909 | 1.0416 | TRUE | new |
| P48740 | MASP1 | MASP1 | Mannan-binding lectin serine protease 1 | 16.8709 | 16.7051 | -0.1659 | 0.0909 | 1.0416 | TRUE | L |
| P61981 | 1433G | YWHAG | 14-3-3 protein gamma | 15.8718 | 16.7552 | 0.8834 | 0.094 | 1.0268 | TRUE | new |
| P27348 | 1433T | YWHAQ | 14-3-3 protein theta | 17.7329 | 16.9749 | -0.7581 | 0.094 | 1.0268 | TRUE | L |
| P62736 | ACTA;ACTH | ACTA2 | Actin, aortic smooth muscle;Actin, gamma-enteric smooth muscle | 18.266 | 17.568 | -0.698 | 0.094 | 1.0268 | TRUE | new |
| P01815 | HV202 |  | Ig heavy chain V-II region COR | 16.0452 | 16.3671 | 0.322 | 0.094 | 1.0268 | TRUE | new |
| P20340 | RAB6A;RB39A;RAB6B | RAB6A | Ras-related protein Rab-6A;Ras-related protein Rab-39A;Ras-related protein Rab-6B | 15.1799 | 17.946 | 2.7661 | 0.094 | 1.0268 | TRUE | new |
| Q96C92 | SDCG3 | SDCCAG3 | Serologically defined colon cancer antigen 3 | 13.9447 | 13.3296 | -0.6151 | 0.094 | 1.0268 | TRUE | new |
| O15061 | SYNEM | SYNM | Synemin | 18.2957 | 18.6831 | 0.3874 | 0.094 | 1.0268 | TRUE | new |
| P68363 | TBA1B | TUBA1B | Tubulin alpha-1B chain | 18.3181 | 17.7087 | -0.6094 | 0.094 | 1.0268 | TRUE | new |
| O14791 | APOL1 | APOL1 | Apolipoprotein L1 | 19.9944 | 19.81 | -0.1844 | 0.103 | 0.987 | TRUE | L.T |
| P24844 | MYL9 | MYL9 | Myosin regulatory light polypeptide 9 | 15.4171 | 16.0456 | 0.6286 | 0.103 | 0.987 | TRUE | new |
| P80108 | PHLD | GPLD1 | Phosphatidylinositol-glycan-specific phospholipase D | 17.9395 | 18.164 | 0.2245 | 0.103 | 0.987 | TRUE | new |
| P20851 | C4BPB | C4BPB | C4b-binding protein beta chain | 18.7567 | 18.9129 | 0.1562 | 0.1073 | 0.9695 | TRUE | new |
| P03952 | KLKB1 | KLKB1 | Plasma kallikrein | 19.2362 | 19.0481 | -0.1882 | 0.1073 | 0.9695 | TRUE | new |
| Q9UIF8 | BAZ2B | BAZ2B | Bromodomain adjacent to zinc finger domain protein 2B | 17.9019 | 18.4866 | 0.5847 | 0.1117 | 0.952 | TRUE | new |
| P02790 | HEMO | HPX | Hemopexin | 25.5076 | 25.6271 | 0.1195 | 0.1117 | 0.952 | TRUE | new |
| P60709 | ACTB | ACTB | Actin, cytoplasmic 1 | 21.1421 | 20.3114 | -0.8307 | 0.1151 | 0.939 | TRUE | new |
| P03951 | FA11 | F11 | Coagulation factor XI | 20.2228 | 20.6752 | 0.4523 | 0.1151 | 0.939 | TRUE | new |
| P01743 | HV102 |  | Ig heavy chain V-I region HG3 | 21.9403 | 21.3001 | -0.6401 | 0.1151 | 0.939 | TRUE | new |
| P63104 | 1433Z | YWHAZ | 14-3-3 protein zeta/delta | 17.2792 | 16.5981 | -0.6811 | 0.1198 | 0.9215 | TRUE | L |
| Q14247 | SRC8 | CTTN | Src substrate cortactin | 15.0927 | 15.4885 | 0.3959 | 0.1198 | 0.9215 | TRUE | new |
| P43652 | AFAM | AFM | Afamin | 20.4133 | 20.589 | 0.1757 | 0.131 | 0.8827 | TRUE | new |
| Q9UBW5 | BIN2 | BIN2 | Bridging integrator 2 | 10.9475 | 10.4033 | -0.5441 | 0.131 | 0.8827 | TRUE | new |
| P04220 | MUCB |  | Ig mu heavy chain disease protein | 23.3989 | 22.9861 | -0.4128 | 0.131 | 0.8827 | TRUE | new |
| P02750 | A2GL | LRG1 | Leucine-rich alpha-2-glycoprotein | 20.4156 | 20.7124 | 0.2968 | 0.1363 | 0.8655 | TRUE | new |
| O75144 | ICOSL | ICOSLG | ICOS ligand | 12.3395 | 12.1119 | -0.2275 | 0.1363 | 0.8655 | TRUE | new |
| P12259 | FA5 | F5 | Coagulation factor V | 16.1872 | 16.3826 | 0.1954 | 0.1405 | 0.8523 | TRUE | new |
| P11226 | MBL2 | MBL2 | Mannose-binding protein C | 16.6077 | 16.2875 | -0.3202 | 0.1405 | 0.8523 | TRUE | L |
| P30101 | PDIA3 | PDIA3 | Protein disulfide-isomerase A3 | 16.0112 | 16.338 | 0.3268 | 0.1405 | 0.8523 | TRUE | new |
| Q9Y490 | TLN1 | TLN1 | Talin-1 | 18.0482 | 17.6443 | -0.4039 | 0.1474 | 0.8314 | TRUE | new |
| P10909 | CLUS | CLU | Clusterin | 23.0342 | 22.9142 | -0.1199 | 0.1533 | 0.8144 | TRUE | new |
| Q96PD5 | PGRP2 | PGLYRP2 | N-acetylmuramoyl-L-alanine amidase | 20.5517 | 20.7129 | 0.1611 | 0.1533 | 0.8144 | TRUE | new |
| P68104 | EF1A1;EF1A2;EF1A3 | EEF1A1 | Elongation factor 1-alpha 1;Elongation factor 1-alpha 2;Putative elongation factor 1-alpha-like 3 | 13.4942 | 13.0209 | -0.4734 | 0.1554 | 0.8086 | TRUE | new |
| P01779 | HV318 |  | Ig heavy chain V-III region TUR | 18.3187 | 18.6453 | 0.3266 | 0.1554 | 0.8086 | TRUE | new |
| O15055 | PER2 | PER2 | Period circadian protein homolog 2 | 21.9854 | 20.292 | -1.6934 | 0.1554 | 0.8086 | TRUE | new |
| O00391 | QSOX1 | QSOX1 | Sulfhydryl oxidase 1 | 16.2899 | 15.828 | -0.462 | 0.1554 | 0.8086 | TRUE | L |
| P02743 | SAMP | APCS | Serum amyloid P-component | 20.199 | 20.396 | 0.197 | 0.1554 | 0.8086 | TRUE | new |
| P01024 | CO3 | C3 | Complement C3 | 24.9414 | 25.036 | 0.0946 | 0.159 | 0.7987 | TRUE | new |
| P02775 | CXCL7 | PPBP | Platelet basic protein | 20.0975 | 19.3398 | -0.7578 | 0.159 | 0.7987 | TRUE | L |
| P00387 | NB5R3 | CYB5R3 | NADH-cytochrome b5 reductase 3 | 14.297 | 14.0586 | -0.2384 | 0.159 | 0.7987 | TRUE | L |
| P19320 | VCAM1 | VCAM1 | Vascular cell adhesion protein 1 | 15.7528 | 15.3951 | -0.3577 | 0.159 | 0.7987 | TRUE | new |
| A4D256 | CC14C | CDC14C | Dual specificity protein phosphatase CDC14C | 13.1029 | 13.8086 | 0.7057 | 0.1614 | 0.792 | TRUE | new |
| P01716 | LV402 |  | Ig lambda chain V-IV region X | 17.0057 | 16.4963 | -0.5093 | 0.1614 | 0.792 | TRUE | new |
| O00187 | MASP2 | MASP2 | Mannan-binding lectin serine protease 2 | 16.2801 | 16.0651 | -0.215 | 0.1614 | 0.792 | TRUE | new |
| P02776 | PLF4 | PF4 | Platelet factor 4 | 19.1679 | 18.4566 | -0.7113 | 0.1614 | 0.792 | TRUE | new |
| P06702 | S10A9 | S100A9 | Protein S100-A9 | 20.0055 | 19.661 | -0.3444 | 0.1614 | 0.792 | TRUE | L |
| P02745 | C1QA | C1QA | Complement C1q subcomponent subunit A | 20.6227 | 20.7183 | 0.0955 | 0.1679 | 0.775 | TRUE | new |
| O95171 | SCEL | SCEL | Sciellin | 16.7817 | 17.1179 | 0.3362 | 0.1679 | 0.775 | TRUE | new |
| P04003 | C4BPA | C4BPA | C4b-binding protein alpha chain | 22.6079 | 22.7662 | 0.1583 | 0.1707 | 0.7678 | TRUE | new |
| Q9UHG0 | DCDC2 | DCDC2 | Doublecortin domain-containing protein 2 | 25.2254 | 24.8868 | -0.3386 | 0.1707 | 0.7678 | TRUE | new |
| P00558 | PGK1 | PGK1 | Phosphoglycerate kinase 1 | 18.1619 | 18.5182 | 0.3563 | 0.1707 | 0.7678 | TRUE | L |
| Q6Q759 | SPG17 | SPAG17 | Sperm-associated antigen 17 | 22.9633 | 22.4445 | -0.5187 | 0.1707 | 0.7678 | TRUE | new |
| A4D1E1 | Z804B | ZNF804B | Zinc finger protein 804B | 16.067 | 16.2419 | 0.1749 | 0.1707 | 0.7678 | TRUE | new |
| P09871 | C1S | C1S | Complement C1s subcomponent | 20.6289 | 20.6891 | 0.0601 | 0.1775 | 0.7508 | TRUE | new |
| Q04637 | IF4G1 | EIF4G1 | Eukaryotic translation initiation factor 4 gamma 1 | 13.8681 | 13.4046 | -0.4635 | 0.1775 | 0.7508 | TRUE | new |
| P60660 | MYL6 | MYL6 | Myosin light polypeptide 6 | 16.608 | 15.9573 | -0.6507 | 0.1859 | 0.7308 | TRUE | new |
| Q9UGM5 | FETUB | FETUB | Fetuin-B | 17.802 | 17.5271 | -0.2748 | 0.1945 | 0.711 | TRUE | A |
| P62258 | 1433E | YWHAE | 14-3-3 protein epsilon | 16.8122 | 17.5233 | 0.7111 | 0.1965 | 0.7066 | TRUE | new |
| P27797 | CALR | CALR | Calreticulin | 17.7429 | 19.1133 | 1.3704 | 0.1965 | 0.7066 | TRUE | new |
| P00488 | F13A | F13A1 | Coagulation factor XIII A chain | 17.8271 | 17.5908 | -0.2363 | 0.1965 | 0.7066 | TRUE | new |
| P62820 | RAB1A;RAB1C;RAB1B | RAB1A | Ras-related protein Rab-1A;Putative Ras-related protein Rab-1C;Ras-related protein Rab-1B | 12.4733 | 13.0252 | 0.5518 | 0.1965 | 0.7066 | TRUE | new |
| P12270 | TPR | TPR | Nucleoprotein TPR | 21.3156 | 20.5849 | -0.7307 | 0.1965 | 0.7066 | TRUE | new |
| Q86UX7 | URP2 | FERMT3 | Fermitin family homolog 3 | 17.2941 | 16.6008 | -0.6933 | 0.1965 | 0.7066 | TRUE | new |
| Q9NXG0 | CNTLN | CNTLN | Centlein | 15.6867 | 16.0921 | 0.4053 | 0.2028 | 0.693 | FALSE | new |
| P09172 | DOPO | DBH | Dopamine beta-hydroxylase | 17.6005 | 17.3244 | -0.2761 | 0.2028 | 0.693 | FALSE | new |
| Q9Y4L1 | HYOU1 | HYOU1 | Hypoxia up-regulated protein 1 | 15.9875 | 14.5805 | -1.407 | 0.2028 | 0.693 | FALSE | new |
| P01781 | HV320 |  | Ig heavy chain V-III region GAL | 21.9022 | 21.6304 | -0.2718 | 0.2106 | 0.6765 | FALSE | new |
| Q9NZ43 | USE1 | USE1 | Vesicle transport protein USE1 | 17.2394 | 16.3488 | -0.8906 | 0.2106 | 0.6765 | FALSE | new |
| Q04917 | 1433F | YWHAH | 14-3-3 protein eta | 14.2997 | 14.1236 | -0.1762 | 0.2131 | 0.6715 | FALSE | new |
| P02768 | ALBU | ALB | Serum albumin | 30.6742 | 30.5594 | -0.1148 | 0.2131 | 0.6715 | FALSE | new |
| P00738 | HPT | HP | Haptoglobin | 26.258 | 26.5447 | 0.2867 | 0.2131 | 0.6715 | FALSE | L.T |
| P01604 | KV112 |  | Ig kappa chain V-I region Kue | 16.8617 | 17.3459 | 0.4842 | 0.2131 | 0.6715 | FALSE | new |
| P0DJI8 | SAA1 | SAA1 | Serum amyloid A-1 protein | 18.0944 | 18.3944 | 0.3001 | 0.2131 | 0.6715 | FALSE | new |
| O75962 | TRIO | TRIO | Triple functional domain protein | 15.9684 | 15.2556 | -0.7128 | 0.2131 | 0.6715 | FALSE | new |
| O15143 | ARC1B | ARPC1B | Actin-related protein 2/3 complex subunit 1B | 17.2204 | 17.9387 | 0.7183 | 0.2158 | 0.666 | FALSE | new |
| Q7Z569 | BRAP | BRAP | BRCA1-associated protein | 15.57 | 14.8501 | -0.7199 | 0.2158 | 0.666 | FALSE | new |
| O43866 | CD5L | CD5L | CD5 antigen-like | 21.2956 | 21.1445 | -0.1511 | 0.2158 | 0.666 | FALSE | new |
| P08709 | FA7 | F7 | Coagulation factor VII | 15.3284 | 15.3619 | 0.0335 | 0.2158 | 0.666 | FALSE | new |
| P01042 | KNG1 | KNG1 | Kininogen-1 | 23.2038 | 23.1214 | -0.0824 | 0.2158 | 0.666 | FALSE | new |
| Q71U36 | TBA1A | TUBA1A | Tubulin alpha-1A chain | 15.3081 | 15.8687 | 0.5605 | 0.2158 | 0.666 | FALSE | new |
| P01824 | HV206 |  | Ig heavy chain V-II region WAH | 19.8338 | 20.6932 | 0.8594 | 0.224 | 0.6497 | FALSE | new |
| P07225 | PROS | PROS1 | Vitamin K-dependent protein S | 20.0543 | 20.1845 | 0.1302 | 0.224 | 0.6497 | FALSE | A |
| Q7Z478 | DHX29 | DHX29 | ATP-dependent RNA helicase DHX29 | 17.9831 | 18.2367 | 0.2536 | 0.2298 | 0.6387 | FALSE | new |
| P01834 | IGKC | IGKC | Ig kappa chain C region | 28.211 | 28.4441 | 0.2331 | 0.2298 | 0.6387 | FALSE | new |
| P01596 | KV104 |  | Ig kappa chain V-I region CAR | 19.5365 | 19.1629 | -0.3736 | 0.2298 | 0.6387 | FALSE | new |
| P04211 | LV001 |  | Ig lambda chain V region 4A | 20.4573 | 20.8148 | 0.3576 | 0.2298 | 0.6387 | FALSE | new |
| Q92820 | GGH | GGH | Gamma-glutamyl hydrolase | 17.6632 | 17.3208 | -0.3424 | 0.2357 | 0.6277 | FALSE | new |
| Q5HYC2 | K2026 | KIAA2026 | Uncharacterized protein KIAA2026 | 16.1394 | 16.387 | 0.2476 | 0.2357 | 0.6277 | FALSE | new |
| P68366 | TBA4A | TUBA4A | Tubulin alpha-4A chain | 16.6659 | 17.3659 | 0.7 | 0.2357 | 0.6277 | FALSE | new |
| P07996 | TSP1 | THBS1 | Thrombospondin-1 | 18.3007 | 17.6778 | -0.6229 | 0.2357 | 0.6277 | FALSE | new |
| P07357 | CO8A | C8A | Complement component C8 alpha chain | 19.4836 | 19.4001 | -0.0835 | 0.2404 | 0.6191 | FALSE | new |
| Q9BS26 | ERP44 | ERP44 | Endoplasmic reticulum resident protein 44 | 14.3035 | 14.8012 | 0.4977 | 0.2404 | 0.6191 | FALSE | L |
| P00740 | FA9 | F9 | Coagulation factor IX | 17.6454 | 17.7626 | 0.1171 | 0.2404 | 0.6191 | FALSE | new |
| P01602 | KV110 | IGKV1-5 | Ig heavy chain V-I region 5 (Fragment) | 19.024 | 18.9063 | -0.1177 | 0.2404 | 0.6191 | FALSE | new |
| Q8WZ42 | TITIN | TTN | Titin | 21.0369 | 21.1303 | 0.0934 | 0.2404 | 0.6191 | FALSE | L |
| Q9C0J8 | WDR33 | WDR33 | pre-mRNA 3' end processing protein WDR33 | 14.8833 | 15.2194 | 0.3361 | 0.2508 | 0.6007 | FALSE | new |
| P04439 | 1A03;1A24;1A11;1A30;1A01;1A23;1A36 | HLA-A | HLA class I histocompatibility antigen, A-[3,24,11,30,23,36] alpha chain | 15.8565 | 16.2811 | 0.4246 | 0.2572 | 0.5897 | FALSE | new |
| O43707 | ACTN4 | ACTN4 | Alpha-actinin-4 | 15.4848 | 16.0053 | 0.5205 | 0.2572 | 0.5897 | FALSE | new |
| P01871 | IGHM | IGHM | Ig mu chain C region | 24.996 | 24.9235 | -0.0724 | 0.2572 | 0.5897 | FALSE | new |
| P19823 | ITIH2 | ITIH2 | Inter-alpha-trypsin inhibitor heavy chain H2 | 23.0032 | 22.9082 | -0.095 | 0.2572 | 0.5897 | FALSE | new |
| O95445 | APOM | APOM | Apolipoprotein M | 20.9155 | 20.7621 | -0.1534 | 0.2596 | 0.5857 | FALSE | new |
| P07360 | CO8G | C8G | Complement component C8 gamma chain | 19.0295 | 18.9316 | -0.0979 | 0.2596 | 0.5857 | FALSE | new |
| P22352 | GPX3 | GPX3 | Glutathione peroxidase 3 | 18.2108 | 18.1803 | -0.0305 | 0.2596 | 0.5857 | FALSE | new |
| P01620 | KV302 |  | Ig kappa chain V-III region SIE | 18.823 | 18.494 | -0.3289 | 0.2596 | 0.5857 | FALSE | new |
| P27918 | PROP | CFP | Properdin | 17.9157 | 17.7791 | -0.1365 | 0.2596 | 0.5857 | FALSE | L |
| P06753 | TPM3 | TPM3 | Tropomyosin alpha-3 chain | 14.7775 | 14.5647 | -0.2128 | 0.2596 | 0.5857 | FALSE | new |
| Q7Z2T5 | TRM1L | TRMT1L | TRMT1-like protein | 14.5341 | 15.9042 | 1.3701 | 0.2596 | 0.5857 | FALSE | new |
| P01011 | AACT | SERPINA3 | Alpha-1-antichymotrypsin | 24.1489 | 24.2381 | 0.0892 | 0.2636 | 0.5791 | FALSE | new |
| P02647 | APOA1 | APOA1 | Apolipoprotein A-I | 27.2502 | 27.1227 | -0.1274 | 0.2636 | 0.5791 | FALSE | new |
| P21333 | FLNA | FLNA | Filamin-A | 17.5711 | 17.2472 | -0.3239 | 0.2636 | 0.5791 | FALSE | L |
| P52566 | GDIR2 | ARHGDIB | Rho GDP-dissociation inhibitor 2 | 17.5005 | 17.7625 | 0.262 | 0.2636 | 0.5791 | FALSE | L |
| P00739 | HPTR | HPR | Haptoglobin-related protein | 20.2806 | 20.0706 | -0.21 | 0.2636 | 0.5791 | FALSE | new |
| P35030 | TRY3 | PRSS3 | Trypsin-3 | 14.2591 | 14.4273 | 0.1682 | 0.2636 | 0.5791 | FALSE | new |
| P01714 | LV301 |  | Ig lambda chain V-III region SH | 21.4103 | 21.1716 | -0.2388 | 0.2732 | 0.5635 | FALSE | new |
| P06317 | LV603 |  | Ig lambda chain V-VI region SUT | 18.8828 | 18.7108 | -0.172 | 0.2732 | 0.5635 | FALSE | new |
| P0C0L5 | CO4B | C4B | Complement C4-B | 20.8322 | 21.0352 | 0.203 | 0.2816 | 0.5503 | FALSE | new |
| P39060 | COIA1 | COL18A1 | Collagen alpha-1(XVIII) chain | 17.9446 | 17.4644 | -0.4802 | 0.2816 | 0.5503 | FALSE | L.T |
| P80748 | LV302 |  | Ig lambda chain V-III region LOI | 22.4292 | 22.2374 | -0.1919 | 0.2816 | 0.5503 | FALSE | new |
| Q12830 | BPTF | BPTF | Nucleosome-remodeling factor subunit BPTF | 13.1741 | 13.6027 | 0.4286 | 0.2847 | 0.5457 | FALSE | new |
| P18065 | IBP2 | IGFBP2 | Insulin-like growth factor-binding protein 2 | 16.5452 | 15.5863 | -0.9589 | 0.2847 | 0.5457 | FALSE | new |
| P01610 | KV118 |  | Ig kappa chain V-I region WEA | 17.8229 | 18.0208 | 0.1979 | 0.2847 | 0.5457 | FALSE | new |
| Q13201 | MMRN1 | MMRN1 | Multimerin-1 | 15.7152 | 15.4561 | -0.2591 | 0.2847 | 0.5457 | FALSE | new |
| P04070 | PROC | PROC | Vitamin K-dependent protein C | 17.0563 | 16.6698 | -0.3865 | 0.2847 | 0.5457 | FALSE | L |
| P37840 | SYUA | SNCA | Alpha-synuclein | 15.568 | 17.265 | 1.6971 | 0.2847 | 0.5457 | FALSE | L |
| P07477 | TRY1 | PRSS1 | Trypsin-1 | 18.0416 | 18.2332 | 0.1916 | 0.2847 | 0.5457 | FALSE | L |
| P02751 | FINC | FN1 | Fibronectin | 22.8843 | 22.992 | 0.1078 | 0.2934 | 0.5326 | FALSE | T |
| Q16787 | LAMA3 | LAMA3 | Laminin subunit alpha-3 | 15.7504 | 16.2464 | 0.4959 | 0.2934 | 0.5326 | FALSE | new |
| P35542 | SAA4 | SAA4 | Serum amyloid A-4 protein | 20.6199 | 20.49 | -0.1298 | 0.2934 | 0.5326 | FALSE | new |
| P04114 | APOB | APOB | Apolipoprotein B-100 | 22.7898 | 22.9044 | 0.1146 | 0.3009 | 0.5216 | FALSE | L |
| P78417 | GSTO1 | GSTO1 | Glutathione S-transferase omega-1 | 14.7408 | 14.0854 | -0.6554 | 0.3009 | 0.5216 | FALSE | L |
| Q14667 | K0100 | KIAA0100 | Protein KIAA0100 | 16.0189 | 16.4678 | 0.4489 | 0.3009 | 0.5216 | FALSE | new |
| P13591 | NCAM1 | NCAM1 | Neural cell adhesion molecule 1 | 14.5331 | 15.4488 | 0.9157 | 0.3009 | 0.5216 | FALSE | L |
| P33908 | MA1A1 | MAN1A1 | Mannosyl-oligosaccharide 1,2-alpha-mannosidase IA | 15.4237 | 15.1554 | -0.2683 | 0.3114 | 0.5067 | FALSE | new |
| Q13061 | TRDN | TRDN | Triadin | 16.63 | 16.8792 | 0.2491 | 0.3114 | 0.5067 | FALSE | new |
| Q14019 | COTL1 | COTL1 | Coactosin-like protein | 14.3702 | 14.2411 | -0.1291 | 0.3192 | 0.4959 | FALSE | T |
| P02042 | HBD | HBD | Hemoglobin subunit delta | 16.8922 | 16.6222 | -0.2701 | 0.3192 | 0.4959 | FALSE | new |
| O00194 | RB27B | RAB27B | Ras-related protein Rab-27B | 14.7681 | 14.4363 | -0.3318 | 0.3192 | 0.4959 | FALSE | new |
| Q6UWP8 | SBSN | SBSN | Suprabasin | 11.2991 | 10.8384 | -0.4606 | 0.3192 | 0.4959 | FALSE | new |
| P11021 | GRP78 | HSPA5 | 78 kDa glucose-regulated protein | 14.856 | 15.3502 | 0.4942 | 0.3229 | 0.4909 | FALSE | new |
| P04196 | HRG | HRG | Histidine-rich glycoprotein | 22.4159 | 22.6184 | 0.2025 | 0.3229 | 0.4909 | FALSE | new |
| Q86VH2 | KIF27 | KIF27 | Kinesin-like protein KIF27 | 15.7076 | 15.078 | -0.6296 | 0.3229 | 0.4909 | FALSE | new |
| P07195 | LDHB | LDHB | L-lactate dehydrogenase B chain | 16.3975 | 16.1141 | -0.2833 | 0.3229 | 0.4909 | FALSE | L |
| P58546 | MTPN | MTPN | Myotrophin | 16.1607 | 17.184 | 1.0233 | 0.3229 | 0.4909 | FALSE | new |
| P61026 | RAB10 | RAB10 | Ras-related protein Rab-10 | 15.5477 | 14.8203 | -0.7274 | 0.3229 | 0.4909 | FALSE | new |
| Q658N2 | WSCD1 | WSCD1 | WSC domain-containing protein 1 | 15.2843 | 15.614 | 0.3297 | 0.3229 | 0.4909 | FALSE | new |
| P08571 | CD14 | CD14 | Monocyte differentiation antigen CD14 | 16.371 | 16.5713 | 0.2003 | 0.3451 | 0.462 | FALSE | new |
| P00450 | CERU | CP | Ceruloplasmin | 23.883 | 23.9013 | 0.0183 | 0.3451 | 0.462 | FALSE | new |
| Q9BWP8 | COL11 | COLEC11 | Collectin-11 | 17.7291 | 18.6522 | 0.9231 | 0.3451 | 0.462 | FALSE | new |
| P09493 | TPM1 | TPM1 | Tropomyosin alpha-1 chain | 12.8312 | 13.6707 | 0.8396 | 0.3451 | 0.462 | FALSE | new |
| Q93084 | AT2A3 | ATP2A3 | Sarcoplasmic/endoplasmic reticulum calcium ATPase 3 | 14.2938 | 13.9839 | -0.3099 | 0.3535 | 0.4516 | FALSE | new |
| Q6PL18 | ATAD2 | ATAD2 | ATPase family AAA domain-containing protein 2 | 19.481 | 20.2801 | 0.7991 | 0.3535 | 0.4516 | FALSE | new |
| P06681 | CO2 | C2 | Complement C2 | 19.3571 | 19.4124 | 0.0554 | 0.3535 | 0.4516 | FALSE | new |
| Q8NFN8 | GP156 | GPR156 | Probable G-protein coupled receptor 156 | 16.379 | 16.3017 | -0.0773 | 0.3535 | 0.4516 | FALSE | new |
| Q8IZP0 | ABI1 | ABI1 | Abl interactor 1 | 16.5351 | 17.0504 | 0.5153 | 0.3576 | 0.4466 | FALSE | new |
| P62158 | CALM | CALM1 | Calmodulin | 18.8803 | 19.4516 | 0.5714 | 0.3576 | 0.4466 | FALSE | new |
| P80419 | HV322 |  | Ig heavy chain V-III region GAR | 19.5986 | 19.4012 | -0.1974 | 0.3576 | 0.4466 | FALSE | new |
| P14618 | KPYM | PKM | Pyruvate kinase PKM | 15.6484 | 15.871 | 0.2226 | 0.3576 | 0.4466 | FALSE | new |
| P55058 | PLTP | PLTP | Phospholipid transfer protein | 17.452 | 17.408 | -0.044 | 0.3576 | 0.4466 | FALSE | new |
| P08294 | SODE | SOD3 | Extracellular superoxide dismutase [Cu-Zn] | 14.9536 | 17.1999 | 2.2463 | 0.3576 | 0.4466 | FALSE | new |
| P02787 | TRFE | TF | Serotransferrin | 26.4512 | 26.3459 | -0.1053 | 0.3576 | 0.4466 | FALSE | L |
| P05154 | IPSP | SERPINA5 | Plasma serine protease inhibitor | 17.719 | 17.623 | -0.096 | 0.3708 | 0.4308 | FALSE | new |
| P01023 | A2MG | A2M | Alpha-2-macroglobulin | 26.0983 | 25.9874 | -0.1109 | 0.3751 | 0.4258 | FALSE | new |
| P02746 | C1QB | C1QB | Complement C1q subcomponent subunit B | 21.6569 | 21.7181 | 0.0612 | 0.3751 | 0.4258 | FALSE | new |
| P35555 | FBN1 | FBN1 | Fibrillin-1 | 15.6127 | 15.3585 | -0.2542 | 0.3751 | 0.4258 | FALSE | new |
| Q75N90 | FBN3 | FBN3 | Fibrillin-3 | 18.6258 | 18.2449 | -0.3809 | 0.3751 | 0.4258 | FALSE | new |
| P01603 | KV111 |  | Ig kappa chain V-I region Ka | 19.1434 | 18.8421 | -0.3014 | 0.3751 | 0.4258 | FALSE | new |
| P01616 | KV203 |  | Ig kappa chain V-II region MIL | 22.7471 | 22.8868 | 0.1397 | 0.3751 | 0.4258 | FALSE | new |
| Q9UHG3 | PCYOX | PCYOX1 | Prenylcysteine oxidase 1 | 15.8452 | 15.6897 | -0.1555 | 0.3751 | 0.4258 | FALSE | new |
| Q9H2X0 | CHRD | CHRD | Chordin | 21.8374 | 21.7877 | -0.0497 | 0.3826 | 0.4173 | FALSE | L |
| P16260 | GDC | SLC25A16 | Graves disease carrier protein | 16.1042 | 15.728 | -0.3762 | 0.3826 | 0.4173 | FALSE | new |
| O14498 | ISLR | ISLR | Immunoglobulin superfamily containing leucine-rich repeat protein | 13.1128 | 12.9178 | -0.1951 | 0.3826 | 0.4173 | FALSE | new |
| Q8IZH2 | XRN1 | XRN1 | 5'-3' exoribonuclease 1 | 14.0462 | 14.5154 | 0.4691 | 0.3826 | 0.4173 | FALSE | new |
| P25311 | ZA2G | AZGP1 | Zinc-alpha-2-glycoprotein | 21.9636 | 22.0102 | 0.0466 | 0.3826 | 0.4173 | FALSE | L |
| P01877 | IGHA2 | IGHA2 | Ig alpha-2 chain C region | 25.8999 | 26.1698 | 0.2699 | 0.3916 | 0.4071 | FALSE | new |
| P61626 | LYSC | LYZ | Lysozyme C | 16.0353 | 16.2861 | 0.2507 | 0.3916 | 0.4071 | FALSE | new |
| Q9H299 | SH3L3 | SH3BGRL3 | SH3 domain-binding glutamic acid-rich-like protein 3 | 15.3914 | 14.9024 | -0.489 | 0.3916 | 0.4071 | FALSE | new |
| P07437 | TBB5 | TUBB | Tubulin beta chain | 18.7161 | 18.5625 | -0.1536 | 0.3916 | 0.4071 | FALSE | new |
| P01889 | 1B07;1B81 | HLA-B | HLA class I histocompatibility antigen, B-7 alpha chain;HLA class I histocompatibility antigen, B-81 alpha chain | 17.1812 | 16.8938 | -0.2873 | 0.3993 | 0.3987 | FALSE | new |
| P02763 | A1AG1 | ORM1 | Alpha-1-acid glycoprotein 1 | 25.7845 | 25.6853 | -0.0992 | 0.3993 | 0.3987 | FALSE | new |
| Q5D0E6 | DALD3 | DALRD3 | DALR anticodon-binding domain-containing protein 3 | 14.9041 | 13.213 | -1.691 | 0.3993 | 0.3987 | FALSE | new |
| P08514 | ITA2B | ITGA2B | Integrin alpha-IIb | 18.1272 | 18.5186 | 0.3913 | 0.3993 | 0.3987 | FALSE | new |
| P01705 | LV202 |  | Ig lambda chain V-II region NEI | 19.4665 | 19.1624 | -0.3041 | 0.3993 | 0.3987 | FALSE | new |
| O43684 | BUB3 | BUB3 | Mitotic checkpoint protein BUB3 | 14.0874 | 14.0265 | -0.0609 | 0.4071 | 0.3903 | FALSE | new |
| Q01459 | DIAC | CTBS | Di-N-acetylchitobiase | 15.3419 | 14.5318 | -0.8101 | 0.4071 | 0.3903 | FALSE | L |
| Q14565 | DMC1 | DMC1 | Meiotic recombination protein DMC1/LIM15 homolog | 15.3395 | 15.8159 | 0.4764 | 0.4071 | 0.3903 | FALSE | new |
| P40197 | GPV | GP5 | Platelet glycoprotein V | 15.3013 | 15.8111 | 0.5098 | 0.4071 | 0.3903 | FALSE | new |
| Q6ZQQ6 | WDR87 | WDR87 | WD repeat-containing protein 87 | 17.165 | 17.3143 | 0.1494 | 0.4071 | 0.3903 | FALSE | new |
| P02760 | AMBP | AMBP | Protein AMBP | 23.1325 | 23.1826 | 0.05 | 0.412 | 0.3851 | FALSE | A |
| P33151 | CADH5 | CDH5 | Cadherin-5 | 16.3246 | 16.5187 | 0.1941 | 0.412 | 0.3851 | FALSE | L |
| P06310 | KV206 |  | Ig kappa chain V-II region RPMI 6410 | 21.2619 | 21.4732 | 0.2113 | 0.412 | 0.3851 | FALSE | new |
| O15049 | N4BP3 | N4BP3 | NEDD4-binding protein 3 | 18.5564 | 18.7721 | 0.2157 | 0.412 | 0.3851 | FALSE | new |
| Q86SQ7 | SDCG8 | SDCCAG8 | Serologically defined colon cancer antigen 8 | 13.8677 | 13.9312 | 0.0635 | 0.412 | 0.3851 | FALSE | new |
| P01137 | TGFB1 | TGFB1 | Transforming growth factor beta-1 | 16.2084 | 16.0028 | -0.2056 | 0.412 | 0.3851 | FALSE | L |
| P02766 | TTHY | TTR | Transthyretin | 20.3717 | 20.2025 | -0.1692 | 0.412 | 0.3851 | FALSE | new |
| P01009 | A1AT | SERPINA1 | Alpha-1-antitrypsin | 26.4975 | 26.4205 | -0.077 | 0.4156 | 0.3814 | FALSE | new |
| Q15582 | BGH3 | TGFBI | Transforming growth factor-beta-induced protein ig-h3 | 16.0733 | 16.1791 | 0.1058 | 0.4156 | 0.3814 | FALSE | L.T |
| P16070 | CD44 | CD44 | CD44 antigen | 15.982 | 16.0516 | 0.0696 | 0.4156 | 0.3814 | FALSE | new |
| P22692 | IBP4 | IGFBP4 | Insulin-like growth factor-binding protein 4 | 16.5005 | 15.7902 | -0.7102 | 0.4156 | 0.3814 | FALSE | new |
| O15037 | KHNYN | KHNYN | Protein KHNYN | 16.3093 | 16.452 | 0.1427 | 0.4156 | 0.3814 | FALSE | new |
| P48059 | LIMS1 | LIMS1 | LIM and senescent cell antigen-like-containing domain protein 1 | 15.7804 | 15.5559 | -0.2246 | 0.4156 | 0.3814 | FALSE | new |
| P01701 | LV103 |  | Ig lambda chain V-I region NEW | 18.3484 | 17.8438 | -0.5046 | 0.4156 | 0.3814 | FALSE | new |
| Q9UNW1 | MINP1 | MINPP1 | Multiple inositol polyphosphate phosphatase 1 | 15.357 | 15.7402 | 0.3831 | 0.4156 | 0.3814 | FALSE | L |
| P00918 | CAH2 | CA2 | Carbonic anhydrase 2 | 16.7747 | 15.6994 | -1.0753 | 0.4251 | 0.3715 | FALSE | new |
| Q05682 | CALD1 | CALD1 | Caldesmon | 13.7879 | 14.2616 | 0.4737 | 0.4251 | 0.3715 | FALSE | new |
| Q9P278 | FNIP2 | FNIP2 | Folliculin-interacting protein 2 | 15.6778 | 16.0471 | 0.3693 | 0.4251 | 0.3715 | FALSE | new |
| P40926 | MDHM | MDH2 | Malate dehydrogenase, mitochondrial | 14.534 | 13.897 | -0.637 | 0.4251 | 0.3715 | FALSE | new |
| Q9NQ79 | CRAC1 | CRTAC1 | Cartilage acidic protein 1 | 18.9597 | 18.811 | -0.1487 | 0.4363 | 0.3602 | FALSE | new |
| P01608 | KV116 |  | Ig kappa chain V-I region Roy | 18.3457 | 18.2641 | -0.0816 | 0.4363 | 0.3602 | FALSE | new |
| Q96RL7 | VP13A | VPS13A | Vacuolar protein sorting-associated protein 13A | 16.9094 | 17.1351 | 0.2256 | 0.4363 | 0.3602 | FALSE | new |
| P09972 | ALDOC | ALDOC | Fructose-bisphosphate aldolase C | 15.6062 | 19.2564 | 3.6502 | 0.4386 | 0.3579 | FALSE | L |
| Q76LX8 | ATS13 | ADAMTS13 | A disintegrin and metalloproteinase with thrombospondin motifs 13 | 19.0506 | 19.4472 | 0.3966 | 0.4386 | 0.3579 | FALSE | L.T |
| Q8TE68 | ES8L1 | EPS8L1 | Epidermal growth factor receptor kinase substrate 8-like protein 1 | 16.0944 | 15.8283 | -0.2661 | 0.4386 | 0.3579 | FALSE | new |
| Q13642 | FHL1 | FHL1 | Four and a half LIM domains protein 1 | 12.8657 | 13.4437 | 0.578 | 0.4386 | 0.3579 | FALSE | new |
| P14314 | GLU2B | PRKCSH | Glucosidase 2 subunit beta | 21.0654 | 20.2435 | -0.8219 | 0.4386 | 0.3579 | FALSE | new |
| P01857 | IGHG1 | IGHG1 | Ig gamma-1 chain C region | 28.1115 | 28.019 | -0.0925 | 0.4386 | 0.3579 | FALSE | new |
| P01600 | KV108 |  | Ig kappa chain V-I region Hau | 23.1229 | 22.9541 | -0.1687 | 0.4386 | 0.3579 | FALSE | new |
| Q2TV78 | MST1L | MST1L | Putative macrophage stimulating 1-like protein | 16.4232 | 16.1119 | -0.3113 | 0.4386 | 0.3579 | FALSE | new |
| Q9Y2K3 | MYH15 | MYH15 | Myosin-15 | 16.6717 | 16.514 | -0.1577 | 0.4386 | 0.3579 | FALSE | new |
| Q6UB35 | C1TM | MTHFD1L | Monofunctional C1-tetrahydrofolate synthase, mitochondrial | 21.4208 | 21.1557 | -0.2651 | 0.4441 | 0.3526 | FALSE | new |
| P07384 | CAN1 | CAPN1 | Calpain-1 catalytic subunit | 14.2514 | 14.0075 | -0.2439 | 0.4441 | 0.3526 | FALSE | new |
| P60953 | CDC42 | CDC42 | Cell division control protein 42 homolog | 11.7188 | 11.8489 | 0.1301 | 0.4441 | 0.3526 | FALSE | L |
| Q9H819 | DJC18 | DNAJC18 | DnaJ homolog subfamily C member 18 | 17.2444 | 16.8408 | -0.4036 | 0.4441 | 0.3526 | FALSE | new |
| Q04756 | HGFA | HGFAC | Hepatocyte growth factor activator | 16.0105 | 16.1277 | 0.1172 | 0.4441 | 0.3526 | FALSE | new |
| P01771 | HV310 |  | Ig heavy chain V-III region HIL | 22.3217 | 22.1646 | -0.1571 | 0.4441 | 0.3526 | FALSE | new |
| P01773 | HV312 |  | Ig heavy chain V-III region BUR | 20.5524 | 20.9401 | 0.3877 | 0.4441 | 0.3526 | FALSE | new |
| P35609 | ACTN2 | ACTN2 | Alpha-actinin-2 | 14.9775 | 13.8341 | -1.1434 | 0.4496 | 0.3472 | FALSE | new |
| Q16658 | FSCN1 | FSCN1 | Fascin | 18.5644 | 19.1892 | 0.6248 | 0.4496 | 0.3472 | FALSE | new |
| P01699 | LV101 |  | Ig lambda chain V-I region VOR | 18.3112 | 18.4545 | 0.1432 | 0.4496 | 0.3472 | FALSE | new |
| P06889 | LV405 |  | Ig lambda chain V-IV region MOL | 17.7479 | 17.6174 | -0.1305 | 0.4496 | 0.3472 | FALSE | new |
| Q86W28 | NALP8 | NLRP8 | NACHT, LRR and PYD domains-containing protein 8 | 14.138 | 14.4726 | 0.3346 | 0.4496 | 0.3472 | FALSE | new |
| O00151 | PDLI1 | PDLIM1 | PDZ and LIM domain protein 1 | 16.0958 | 15.832 | -0.2638 | 0.4496 | 0.3472 | FALSE | new |
| Q6UXB8 | PI16 | PI16 | Peptidase inhibitor 16 | 15.4009 | 15.3073 | -0.0937 | 0.4496 | 0.3472 | FALSE | new |
| P15814 | IGLL1 | IGLL1 | Immunoglobulin lambda-like polypeptide 1 | 16.8434 | 16.9289 | 0.0855 | 0.4595 | 0.3377 | FALSE | L |
| P55103 | INHBC | INHBC | Inhibin beta C chain | 17.2524 | 17.145 | -0.1074 | 0.4595 | 0.3377 | FALSE | L |
| O00159 | MYO1C | MYO1C | Unconventional myosin-Ic | 13.3094 | 13.6185 | 0.3091 | 0.4595 | 0.3377 | FALSE | new |
| P01833 | PIGR | PIGR | Polymeric immunoglobulin receptor | 16.4392 | 18.8976 | 2.4584 | 0.4595 | 0.3377 | FALSE | L |
| Q96KN2 | CNDP1 | CNDP1 | Beta-Ala-His dipeptidase | 16.8559 | 16.6866 | -0.1692 | 0.4681 | 0.3297 | FALSE | new |
| P06331 | HV209 |  | Ig heavy chain V-II region ARH-77 | 20.9432 | 20.8019 | -0.1413 | 0.4681 | 0.3297 | FALSE | new |
| P05155 | IC1 | SERPING1 | Plasma protease C1 inhibitor | 23.6091 | 23.5463 | -0.0629 | 0.4681 | 0.3297 | FALSE | L.T |
| P13645 | K1C10 | KRT10 | Keratin, type I cytoskeletal 10 | 15.7232 | 15.2538 | -0.4694 | 0.4681 | 0.3297 | FALSE | new |
| P01609 | KV117 |  | Ig kappa chain V-I region Scw | 19.6849 | 19.8605 | 0.1756 | 0.4681 | 0.3297 | FALSE | new |
| P16615 | AT2A2 | ATP2A2 | Sarcoplasmic/endoplasmic reticulum calcium ATPase 2 | 17.3593 | 16.9967 | -0.3626 | 0.4752 | 0.3231 | FALSE | new |
| P27487 | DPP4 | DPP4 | Dipeptidyl peptidase 4 | 15.7485 | 15.7084 | -0.0402 | 0.4752 | 0.3231 | FALSE | new |
| P01880 | IGHD | IGHD | Ig delta chain C region | 20.0864 | 19.9851 | -0.1013 | 0.4752 | 0.3231 | FALSE | new |
| Q9UHV7 | MED13 | MED13 | Mediator of RNA polymerase II transcription subunit 13 | 16.2644 | 16.444 | 0.1796 | 0.4752 | 0.3231 | FALSE | new |
| P61224 | RAP1B | RAP1B | Ras-related protein Rap-1b | 17.068 | 17.1731 | 0.105 | 0.4752 | 0.3231 | FALSE | new |
| P28290 | SSFA2 | SSFA2 | Sperm-specific antigen 2 | 21.6137 | 21.3251 | -0.2886 | 0.4752 | 0.3231 | FALSE | new |
| P07339 | CATD | CTSD | Cathepsin D | 14.0632 | 13.8446 | -0.2186 | 0.4796 | 0.3191 | FALSE | new |
| Q00610 | CLH1 | CLTC | Clathrin heavy chain 1 | 15.4999 | 14.9811 | -0.5188 | 0.4796 | 0.3191 | FALSE | new |
| Q8IYI6 | EXOC8 | EXOC8 | Exocyst complex component 8 | 18.7762 | 18.6146 | -0.1615 | 0.4796 | 0.3191 | FALSE | new |
| P01612 | KV120 |  | Ig kappa chain V-I region Mev | 20.518 | 21.1027 | 0.5847 | 0.4796 | 0.3191 | FALSE | new |
| P01617 | KV204 |  | Ig kappa chain V-II region TEW | 21.3541 | 21.5234 | 0.1693 | 0.4796 | 0.3191 | FALSE | new |
| P06318 | LV604;LV605 |  | Ig lambda chain V-VI region WLT;Ig lambda chain V-VI region EB4 | 18.1542 | 18.27 | 0.1158 | 0.4796 | 0.3191 | FALSE | new |
| P98160 | PGBM | HSPG2 | Basement membrane-specific heparan sulfate proteoglycan core protein | 15.7271 | 15.2362 | -0.4909 | 0.4796 | 0.3191 | FALSE | new |
| P10599 | THIO | TXN | Thioredoxin | 13.8386 | 14.6863 | 0.8478 | 0.4796 | 0.3191 | FALSE | new |
| P06576 | ATPB | ATP5B | ATP synthase subunit beta, mitochondrial | 13.4206 | 14.0193 | 0.5986 | 0.484 | 0.3151 | FALSE | L |
| Q8TDI0 | CHD5 | CHD5 | Chromodomain-helicase-DNA-binding protein 5 | 20.8115 | 20.7758 | -0.0357 | 0.484 | 0.3151 | FALSE | new |
| P13671 | CO6 | C6 | Complement component C6 | 19.4877 | 19.5358 | 0.0481 | 0.484 | 0.3151 | FALSE | L.T |
| P07900 | HS90A | HSP90AA1 | Heat shock protein HSP 90-alpha | 16.5357 | 16.5626 | 0.0269 | 0.484 | 0.3151 | FALSE | new |
| P01876 | IGHA1 | IGHA1 | Ig alpha-1 chain C region | 24.7992 | 24.9874 | 0.1881 | 0.484 | 0.3151 | FALSE | new |
| P01861 | IGHG4 | IGHG4 | Ig gamma-4 chain C region | 22.9392 | 22.7618 | -0.1774 | 0.484 | 0.3151 | FALSE | L |
| Q9Y5Y7 | LYVE1 | LYVE1 | Lymphatic vessel endothelial hyaluronic acid receptor 1 | 14.9803 | 15.0583 | 0.078 | 0.484 | 0.3151 | FALSE | new |
| Q6IBS0 | TWF2 | TWF2 | Twinfilin-2 | 16.3247 | 16.7923 | 0.4676 | 0.484 | 0.3151 | FALSE | new |
| P04217 | A1BG | A1BG | Alpha-1B-glycoprotein | 23.2321 | 23.2967 | 0.0646 | 0.4928 | 0.3073 | FALSE | new |
| P08758 | ANXA5 | ANXA5 | Annexin A5 | 14.2918 | 15.3966 | 1.1048 | 0.4928 | 0.3073 | FALSE | new |
| P01772 | HV311 |  | Ig heavy chain V-III region KOL | 18.9741 | 19.2304 | 0.2563 | 0.4928 | 0.3073 | FALSE | new |
| P22891 | PROZ | PROZ | Vitamin K-dependent protein Z | 16.4237 | 16.7443 | 0.3206 | 0.4928 | 0.3073 | FALSE | new |
| Q6RFH5 | WDR74 | WDR74 | WD repeat-containing protein 74 | 16.1721 | 16.0697 | -0.1024 | 0.4928 | 0.3073 | FALSE | new |
| Q9BQI0 | AIF1L | AIF1L | Allograft inflammatory factor 1-like | 17.1579 | 17.2714 | 0.1135 | 0.496 | 0.3045 | FALSE | new |
| P06276 | CHLE | BCHE | Cholinesterase | 17.2027 | 17.2769 | 0.0743 | 0.496 | 0.3045 | FALSE | new |
| P08637 | FCG3A | FCGR3A | Low affinity immunoglobulin gamma Fc region receptor III-A | 15.0273 | 15.0705 | 0.0432 | 0.496 | 0.3045 | FALSE | new |
| P69905 | HBA | HBA1 | Hemoglobin subunit alpha | 20.9976 | 20.81 | -0.1875 | 0.496 | 0.3045 | FALSE | new |
| P35527 | K1C9 | KRT9 | Keratin, type I cytoskeletal 9 | 15.6415 | 15.8039 | 0.1624 | 0.496 | 0.3045 | FALSE | new |
| Q9UPN3 | MACF1 | MACF1 | Microtubule-actin cross-linking factor 1, isoforms 1/2/3/5 | 18.0353 | 18.0048 | -0.0305 | 0.496 | 0.3045 | FALSE | new |
| Q8TDX9 | PK1L1 | PKD1L1 | Polycystic kidney disease protein 1-like 1 | 15.3522 | 15.312 | -0.0403 | 0.496 | 0.3045 | FALSE | new |
| Q15063 | POSTN | POSTN | Periostin | 17.5867 | 17.4726 | -0.1141 | 0.496 | 0.3045 | FALSE | L.T |
| P09486 | SPRC | SPARC | SPARC | 17.0578 | 16.924 | -0.1338 | 0.496 | 0.3045 | FALSE | L |
| Q9C0F0 | ASXL3 | ASXL3 | Putative Polycomb group protein ASXL3 | 17.908 | 17.1965 | -0.7116 | 0.5006 | 0.3005 | FALSE | new |
| Q01518 | CAP1 | CAP1 | Adenylyl cyclase-associated protein 1 | 16.1218 | 16.7222 | 0.6004 | 0.5006 | 0.3005 | FALSE | new |
| P10645 | CMGA | CHGA | Chromogranin-A | 15.1165 | 14.9315 | -0.185 | 0.5006 | 0.3005 | FALSE | L |
| P12111 | CO6A3 | COL6A3 | Collagen alpha-3(VI) chain | 17.1946 | 17.0286 | -0.166 | 0.5006 | 0.3005 | FALSE | new |
| P36980 | FHR2 | CFHR2 | Complement factor H-related protein 2 | 17.9728 | 18.0705 | 0.0977 | 0.5006 | 0.3005 | FALSE | new |
| Q9BY32 | ITPA | ITPA | Inosine triphosphate pyrophosphatase | 14.3247 | 14.0604 | -0.2643 | 0.5006 | 0.3005 | FALSE | new |
| Q9NP80 | PLPL8 | PNPLA8 | Calcium-independent phospholipase A2-gamma | 18.4738 | 18.5401 | 0.0664 | 0.5006 | 0.3005 | FALSE | new |
| P02788 | TRFL | LTF | Lactotransferrin | 17.8136 | 17.7229 | -0.0907 | 0.5006 | 0.3005 | FALSE | L |
| O15144 | ARPC2 | ARPC2 | Actin-related protein 2/3 complex subunit 2 | 14.0672 | 13.3569 | -0.7103 | 0.5067 | 0.2952 | FALSE | new |
| P15169 | CBPN | CPN1 | Carboxypeptidase N catalytic chain | 18.188 | 18.0869 | -0.1011 | 0.5067 | 0.2952 | FALSE | new |
| Q6YHK3 | CD109 | CD109 | CD109 antigen | 14.0906 | 14.4115 | 0.3209 | 0.5067 | 0.2952 | FALSE | new |
| O15230 | LAMA5 | LAMA5 | Laminin subunit alpha-5 | 13.928 | 14.3982 | 0.4701 | 0.5067 | 0.2952 | FALSE | new |
| Q13093 | PAFA | PLA2G7 | Platelet-activating factor acetylhydrolase | 15.6592 | 15.7846 | 0.1254 | 0.5067 | 0.2952 | FALSE | new |
| Q15404 | RSU1 | RSU1 | Ras suppressor protein 1 | 16.3863 | 16.158 | -0.2284 | 0.5067 | 0.2952 | FALSE | new |
| P0CG47 | UBB;UBC;RS27A;RL40 | UBB | Polyubiquitin-B;Polyubiquitin-C;Ubiquitin-40S ribosomal protein S27a;Ubiquitin-60S ribosomal protein L40 | 14.1256 | 14.1586 | 0.0331 | 0.5067 | 0.2952 | FALSE | L |
| Q9NZP8 | C1RL | C1RL | Complement C1r subcomponent-like protein | 18.7478 | 18.7032 | -0.0446 | 0.5156 | 0.2877 | FALSE | L |
| P23083 | HV103 |  | Ig heavy chain V-I region V35 | 19.0802 | 18.6956 | -0.3846 | 0.5156 | 0.2877 | FALSE | new |
| P29622 | KAIN | SERPINA4 | Kallistatin | 19.191 | 18.8143 | -0.3767 | 0.5156 | 0.2877 | FALSE | L |
| P01614 | KV201 |  | Ig kappa chain V-II region Cum | 22.8189 | 22.9402 | 0.1213 | 0.5156 | 0.2877 | FALSE | new |
| Q8NBP7 | PCSK9 | PCSK9 | Proprotein convertase subtilisin/kexin type 9 | 16.1426 | 15.4688 | -0.6738 | 0.5156 | 0.2877 | FALSE | new |
| P61158 | ARP3 | ACTR3 | Actin-related protein 3 | 16.0145 | 15.8591 | -0.1554 | 0.519 | 0.2848 | FALSE | new |
| P49747 | COMP | COMP | Cartilage oligomeric matrix protein | 18.1114 | 18.2725 | 0.1611 | 0.519 | 0.2848 | FALSE | new |
| P35908 | K22E | KRT2 | Keratin, type II cytoskeletal 2 epidermal | 15.8132 | 14.3426 | -1.4706 | 0.519 | 0.2848 | FALSE | new |
| P04430 | KV122 |  | Ig kappa chain V-I region BAN | 19.7012 | 19.5013 | -0.2 | 0.519 | 0.2848 | FALSE | new |
| P04431 | KV123 |  | Ig kappa chain V-I region Walker | 18.0464 | 17.9246 | -0.1218 | 0.519 | 0.2848 | FALSE | new |
| P01625 | KV402 |  | Ig kappa chain V-IV region Len | 23.7989 | 23.7252 | -0.0737 | 0.519 | 0.2848 | FALSE | new |
| P10646 | TFPI1 | TFPI | Tissue factor pathway inhibitor | 13.7734 | 13.2594 | -0.514 | 0.519 | 0.2848 | FALSE | L.T |
| P52888 | THOP1 | THOP1 | Thimet oligopeptidase | 14.1555 | 14.4078 | 0.2523 | 0.519 | 0.2848 | FALSE | new |
| P01033 | TIMP1 | TIMP1 | Metalloproteinase inhibitor 1 | 14.7474 | 14.5178 | -0.2295 | 0.519 | 0.2848 | FALSE | new |
| P47756 | CAPZB | CAPZB | F-actin-capping protein subunit beta | 15.4444 | 15.6039 | 0.1595 | 0.5239 | 0.2808 | FALSE | new |
| P21926 | CD9 | CD9 | CD9 antigen | 17.6383 | 17.2826 | -0.3556 | 0.5239 | 0.2808 | FALSE | new |
| P06744 | G6PI | GPI | Glucose-6-phosphate isomerase | 19.3861 | 19.1272 | -0.2589 | 0.5239 | 0.2808 | FALSE | L |
| P14770 | GPIX | GP9 | Platelet glycoprotein IX | 15.1459 | 14.9087 | -0.2372 | 0.5239 | 0.2808 | FALSE | new |
| P68871 | HBB | HBB | Hemoglobin subunit beta | 22.4958 | 22.3566 | -0.1391 | 0.5239 | 0.2808 | FALSE | new |
| P05013 | IFNA6 | IFNA6 | Interferon alpha-6 | 15.1632 | 16.1158 | 0.9526 | 0.5239 | 0.2808 | FALSE | new |
| Q9NPH3 | IL1AP | IL1RAP | Interleukin-1 receptor accessory protein | 14.9089 | 14.9653 | 0.0564 | 0.5239 | 0.2808 | FALSE | new |
| P11279 | LAMP1 | LAMP1 | Lysosome-associated membrane glycoprotein 1 | 16.3232 | 16.4208 | 0.0976 | 0.5239 | 0.2808 | FALSE | new |
| Q9BXR6 | FHR5 | CFHR5 | Complement factor H-related protein 5 | 15.9166 | 16.0217 | 0.1051 | 0.5327 | 0.2735 | FALSE | new |
| P05546 | HEP2 | SERPIND1 | Heparin cofactor 2 | 20.7249 | 20.6288 | -0.0961 | 0.5327 | 0.2735 | FALSE | new |
| P04207 | KV308 |  | Ig kappa chain V-III region CLL | 16.8473 | 16.9084 | 0.0611 | 0.5327 | 0.2735 | FALSE | new |
| P43121 | MUC18 | MCAM | Cell surface glycoprotein MUC18 | 13.9165 | 13.3045 | -0.612 | 0.5327 | 0.2735 | FALSE | new |
| O60292 | SI1L3 | SIPA1L3 | Signal-induced proliferation-associated 1-like protein 3 | 13.6434 | 14.2306 | 0.5872 | 0.5327 | 0.2735 | FALSE | new |
| P00736 | C1R | C1R | Complement C1r subcomponent | 19.2856 | 19.3743 | 0.0887 | 0.5402 | 0.2674 | FALSE | new |
| Q16595 | FRDA | FXN | Frataxin, mitochondrial | 17.0129 | 17.179 | 0.1662 | 0.5402 | 0.2674 | FALSE | new |
| P05106 | ITB3 | ITGB3 | Integrin beta-3 | 16.8248 | 16.7088 | -0.116 | 0.5402 | 0.2674 | FALSE | new |
| Q68DE3 | K2018 | KIAA2018 | Basic helix-loop-helix domain-containing protein KIAA2018 | 15.2472 | 15.4641 | 0.217 | 0.5402 | 0.2674 | FALSE | new |
| P32119 | PRDX2 | PRDX2 | Peroxiredoxin-2 | 16.6109 | 17.0435 | 0.4327 | 0.5402 | 0.2674 | FALSE | new |
| P04004 | VTNC | VTN | Vitronectin | 23.7259 | 23.6497 | -0.0761 | 0.5402 | 0.2674 | FALSE | L |
| P0C6C1 | AN34C | ANKRD34C | Ankyrin repeat domain-containing protein 34C | 15.0528 | 14.998 | -0.0548 | 0.5425 | 0.2656 | FALSE | new |
| P02655 | APOC2 | APOC2 | Apolipoprotein C-II | 21.6176 | 21.7882 | 0.1706 | 0.5425 | 0.2656 | FALSE | new |
| P31146 | COR1A | CORO1A | Coronin-1A | 17.3045 | 17.2126 | -0.0919 | 0.5425 | 0.2656 | FALSE | new |
| Q9UKJ3 | GPTC8 | GPATCH8 | G patch domain-containing protein 8 | 18.2197 | 18.2411 | 0.0214 | 0.5425 | 0.2656 | FALSE | new |
| P01778 | HV317 |  | Ig heavy chain V-III region ZAP | 19.3014 | 19.2112 | -0.0902 | 0.5425 | 0.2656 | FALSE | new |
| P10721 | KIT | KIT | Mast/stem cell growth factor receptor Kit | 13.0862 | 13.3722 | 0.286 | 0.5425 | 0.2656 | FALSE | L |
| P01605 | KV113 |  | Ig kappa chain V-I region Lay | 19.1668 | 19.088 | -0.0787 | 0.5425 | 0.2656 | FALSE | new |
| P01613 | KV121 |  | Ig kappa chain V-I region Ni | 21.8301 | 21.8743 | 0.0441 | 0.5425 | 0.2656 | FALSE | new |
| P00338 | LDHA | LDHA | L-lactate dehydrogenase A chain | 16.6865 | 16.5257 | -0.1607 | 0.5425 | 0.2656 | FALSE | new |
| P06887 | LV108 |  | Ig lambda chain V-I region MEM | 18.8435 | 18.6734 | -0.1701 | 0.5425 | 0.2656 | FALSE | new |
| P04075 | ALDOA | ALDOA | Fructose-bisphosphate aldolase A | 16.1924 | 16.1757 | -0.0166 | 0.5448 | 0.2638 | FALSE | new |
| P02649 | APOE | APOE | Apolipoprotein E | 21.23 | 21.2809 | 0.0509 | 0.5448 | 0.2638 | FALSE | new |
| P07358 | CO8B | C8B | Complement component C8 beta chain | 18.2613 | 18.2391 | -0.0222 | 0.5448 | 0.2638 | FALSE | new |
| P14625 | ENPL | HSP90B1 | Endoplasmin | 14.593 | 14.4007 | -0.1923 | 0.5448 | 0.2638 | FALSE | new |
| Q9UNN8 | EPCR | PROCR | Endothelial protein C receptor | 15.8976 | 15.9279 | 0.0303 | 0.5448 | 0.2638 | FALSE | new |
| P13224 | GP1BB | GP1BB | Platelet glycoprotein Ib beta chain | 17.9165 | 18.3363 | 0.4197 | 0.5448 | 0.2638 | FALSE | new |
| P10412 | H14;H13;H12;H1T;H11 | HIST1H1E | Histone H1.4;Histone H1.3;Histone H1.2;Histone H1t;Histone H1.1 | 14.3355 | 14.4971 | 0.1616 | 0.5448 | 0.2638 | FALSE | new |
| P01742 | HV101 |  | Ig heavy chain V-I region EU | 22.7853 | 22.8264 | 0.0412 | 0.5448 | 0.2638 | FALSE | new |
| P01764 | HV303 | IGHV3-23 | Ig heavy chain V-III region 23 | 22.3969 | 22.3322 | -0.0647 | 0.5448 | 0.2638 | FALSE | new |
| P01776 | HV315 |  | Ig heavy chain V-III region WAS | 21.3283 | 21.4216 | 0.0933 | 0.5448 | 0.2638 | FALSE | new |
| P01593 | KV101 |  | Ig kappa chain V-I region AG | 12.6282 | 12.9402 | 0.312 | 0.5448 | 0.2638 | FALSE | new |
| P01594 | KV102 |  | Ig kappa chain V-I region AU | 18.2371 | 18.1152 | -0.1219 | 0.5448 | 0.2638 | FALSE | new |
| P06309 | KV205 |  | Ig kappa chain V-II region GM607 (Fragment) | 16.8027 | 17.3717 | 0.569 | 0.5448 | 0.2638 | FALSE | new |
| P01703 | LV105 |  | Ig lambda chain V-I region NEWM | 19.8931 | 19.7657 | -0.1275 | 0.5448 | 0.2638 | FALSE | new |
| P01710 | LV207 |  | Ig lambda chain V-II region BO | 14.8664 | 15.752 | 0.8857 | 0.5448 | 0.2638 | FALSE | new |
| P15151 | PVR | PVR | Poliovirus receptor | 14.2121 | 14.2948 | 0.0828 | 0.5448 | 0.2638 | FALSE | new |
| P0C7M3 | SFTA3 | SFTA3 | Surfactant-associated protein 3 | 15.5531 | 15.4382 | -0.1149 | 0.5448 | 0.2638 | FALSE | new |
| Q13103 | SPP24 | SPP2 | Secreted phosphoprotein 24 | 16.8596 | 17.2387 | 0.3792 | 0.5448 | 0.2638 | FALSE | new |
| P27105 | STOM | STOM | Erythrocyte band 7 integral membrane protein | 15.7981 | 16.0624 | 0.2642 | 0.5448 | 0.2638 | FALSE | new |
| P68371 | TBB4B | TUBB4B | Tubulin beta-4B chain | 15.7879 | 15.4104 | -0.3776 | 0.5448 | 0.2638 | FALSE | new |
| P05543 | THBG | SERPINA7 | Thyroxine-binding globulin | 17.644 | 17.6833 | 0.0393 | 0.5448 | 0.2638 | FALSE | new |
| O94892 | ZN432 | ZNF432 | Zinc finger protein 432 | 19.0995 | 19.1536 | 0.0542 | 0.5448 | 0.2638 | FALSE | new |
| P05062 | ALDOB | ALDOB | Fructose-bisphosphate aldolase B | 15.8648 | 15.7387 | -0.126 | 0.5523 | 0.2579 | FALSE | new |
| P12830 | CADH1 | CDH1 | Cadherin-1 | 17.1146 | 17.1491 | 0.0345 | 0.5523 | 0.2579 | FALSE | new |
| P01763 | HV302 |  | Ig heavy chain V-III region WEA | 20.1522 | 20.3387 | 0.1865 | 0.5523 | 0.2579 | FALSE | new |
| P06311 | KV311 |  | Ig kappa chain V-III region IARC/BL41 | 17.4027 | 17.4262 | 0.0235 | 0.5523 | 0.2579 | FALSE | new |
| P08567 | PLEK | PLEK | Pleckstrin | 16.4866 | 16.2989 | -0.1877 | 0.5523 | 0.2579 | FALSE | new |
| P05109 | S10A8 | S100A8 | Protein S100-A8 | 16.6833 | 16.5491 | -0.1341 | 0.5523 | 0.2579 | FALSE | new |
| P02747 | C1QC | C1QC | Complement C1q subcomponent subunit C | 21.6993 | 21.7536 | 0.0542 | 0.5585 | 0.253 | FALSE | new |
| P62873 | GBB1;GBB2;GBB4 | GNB1 | Guanine nucleotide-binding protein G(I)/G(S)/G(T) subunit beta-1;Guanine nucleotide-binding protein G(I)/G(S)/G(T) subunit beta-2;Guanine nucleotide-binding protein subunit beta-4 | 14.3623 | 14.4733 | 0.1111 | 0.5585 | 0.253 | FALSE | new |
| P08253 | MMP2 | MMP2 | 72 kDa type IV collagenase | 17.2331 | 16.7952 | -0.4379 | 0.5585 | 0.253 | FALSE | new |
| P00491 | PNPH | PNP | Purine nucleoside phosphorylase | 17.6558 | 17.6279 | -0.028 | 0.5585 | 0.253 | FALSE | new |
| P23284 | PPIB | PPIB | Peptidyl-prolyl cis-trans isomerase B | 16.4873 | 16.5404 | 0.0531 | 0.5585 | 0.253 | FALSE | new |
| Q12913 | PTPRJ | PTPRJ | Receptor-type tyrosine-protein phosphatase eta | 17.4686 | 17.8657 | 0.3971 | 0.5585 | 0.253 | FALSE | new |
| P08670 | VIME | VIM | Vimentin | 17.6248 | 17.6003 | -0.0244 | 0.5585 | 0.253 | FALSE | new |
| Q96P64 | AGAP4 | AGAP4 | Arf-GAP with GTPase, ANK repeat and PH domain-containing protein 4 | 20.683 | 20.5394 | -0.1436 | 0.5646 | 0.2482 | FALSE | new |
| P06727 | APOA4 | APOA4 | Apolipoprotein A-IV | 22.6338 | 22.6395 | 0.0057 | 0.5646 | 0.2482 | FALSE | A |
| P06733 | ENOA | ENO1 | Alpha-enolase | 15.8507 | 15.9317 | 0.0811 | 0.5646 | 0.2482 | FALSE | new |
| P63241 | IF5A1 | EIF5A | Eukaryotic translation initiation factor 5A-1 | 16.3325 | 16.405 | 0.0725 | 0.5646 | 0.2482 | FALSE | L |
| P04264 | K2C1 | KRT1 | Keratin, type II cytoskeletal 1 | 17.0256 | 17.3665 | 0.3408 | 0.5646 | 0.2482 | FALSE | new |
| P06888 | LV109 |  | Ig lambda chain V-I region EPS | 19.0793 | 19.2087 | 0.1294 | 0.5646 | 0.2482 | FALSE | new |
| Q9BUN1 | MENT | MENT | Protein MENT | 16.3003 | 16.1223 | -0.1779 | 0.5646 | 0.2482 | FALSE | L |
| P21291 | CSRP1 | CSRP1 | Cysteine and glycine-rich protein 1 | 16.5825 | 16.0558 | -0.5267 | 0.5831 | 0.2343 | FALSE | new |
| P99999 | CYC | CYCS | Cytochrome c | 14.4346 | 14.5519 | 0.1172 | 0.5831 | 0.2343 | FALSE | L |
| P01775 | HV314 |  | Ig heavy chain V-III region LAY | 17.9387 | 17.2547 | -0.684 | 0.5831 | 0.2343 | FALSE | new |
| P48735 | IDHP | IDH2 | Isocitrate dehydrogenase [NADP], mitochondrial | 17.7107 | 18.1532 | 0.4425 | 0.5831 | 0.2343 | FALSE | new |
| Q27J81 | INF2 | INF2 | Inverted formin-2 | 16.0874 | 16.0383 | -0.0491 | 0.5831 | 0.2343 | FALSE | new |
| P01713 | LV210 |  | Ig lambda chain V-II region NIG-58 | 15.9113 | 16.1471 | 0.2358 | 0.5831 | 0.2343 | FALSE | new |
| O14786 | NRP1 | NRP1 | Neuropilin-1 | 14.0836 | 14.2619 | 0.1783 | 0.5831 | 0.2343 | FALSE | new |
| P18206 | VINC | VCL | Vinculin | 16.3196 | 16.5532 | 0.2335 | 0.5831 | 0.2343 | FALSE | new |
| P04275 | VWF | VWF | von Willebrand factor | 16.2025 | 16.2592 | 0.0567 | 0.5831 | 0.2343 | FALSE | new |
| Q15848 | ADIPO | ADIPOQ | Adiponectin | 16.9847 | 16.9444 | -0.0403 | 0.5904 | 0.2289 | FALSE | new |
| Q96IY4 | CBPB2 | CPB2 | Carboxypeptidase B2 | 18.2199 | 18.1802 | -0.0397 | 0.5904 | 0.2289 | FALSE | new |
| O95866 | G6B | G6B | Protein G6b | 15.6747 | 15.485 | -0.1898 | 0.5904 | 0.2289 | FALSE | L |
| P80422 | LV212 |  | Ig gamma lambda chain V-II region DOT | 19.4488 | 19.5465 | 0.0977 | 0.5904 | 0.2289 | FALSE | new |
| P12955 | PEPD | PEPD | Xaa-Pro dipeptidase | 16.4756 | 16.5404 | 0.0648 | 0.5904 | 0.2289 | FALSE | new |
| Q9BYB0 | SHAN3 | SHANK3 | SH3 and multiple ankyrin repeat domains protein 3 | 15.7854 | 15.4405 | -0.3449 | 0.5904 | 0.2289 | FALSE | new |
| P01607 | KV115 |  | Ig kappa chain V-I region Rei | 18.3942 | 18.5946 | 0.2005 | 0.5951 | 0.2254 | FALSE | new |
| P01622 | KV304 |  | Ig kappa chain V-III region Ti | 19.0663 | 19.1241 | 0.0578 | 0.5951 | 0.2254 | FALSE | new |
| P18135 | KV312 |  | Ig kappa chain V-III region HAH | 24.0338 | 24.0621 | 0.0283 | 0.5951 | 0.2254 | FALSE | new |
| P18136 | KV313 |  | Ig kappa chain V-III region HIC | 17.1561 | 17.23 | 0.0739 | 0.5951 | 0.2254 | FALSE | new |
| A0M8Q6 | LAC7 | IGLC7 | Ig lambda-7 chain C region | 21.3627 | 21.2691 | -0.0936 | 0.5951 | 0.2254 | FALSE | new |
| P01702 | LV104;LV107 |  | Ig lambda chain V-I region NIG-64;Ig lambda chain V-I region BL2 | 19.6433 | 19.7696 | 0.1263 | 0.5951 | 0.2254 | FALSE | new |
| O14950 | ML12B;ML12A | MYL12B | Myosin regulatory light chain 12B;Myosin regulatory light chain 12A | 16.6492 | 16.6345 | -0.0147 | 0.5951 | 0.2254 | FALSE | new |
| P02753 | RET4 | RBP4 | Retinol-binding protein 4 | 20.2776 | 20.2554 | -0.0222 | 0.5951 | 0.2254 | FALSE | new |
| P05090 | APOD | APOD | Apolipoprotein D | 22.0919 | 22.0752 | -0.0166 | 0.5974 | 0.2237 | FALSE | new |
| P61160 | ARP2 | ACTR2 | Actin-related protein 2 | 15.7931 | 15.6426 | -0.1505 | 0.5974 | 0.2237 | FALSE | new |
| P30043 | BLVRB | BLVRB | Flavin reductase (NADPH) | 15.1428 | 15.6522 | 0.5094 | 0.5974 | 0.2237 | FALSE | new |
| Q6ZR08 | DYH12 | DNAH12 | Dynein heavy chain 12, axonemal | 17.4527 | 17.3869 | -0.0658 | 0.5974 | 0.2237 | FALSE | new |
| P01766 | HV305 |  | Ig heavy chain V-III region BRO | 24.3475 | 24.4132 | 0.0657 | 0.5974 | 0.2237 | FALSE | new |
| B9A064 | IGLL5 | IGLL5 | Immunoglobulin lambda-like polypeptide 5 | 25.3883 | 25.3997 | 0.0113 | 0.5974 | 0.2237 | FALSE | new |
| P04206 | KV307 |  | Ig kappa chain V-III region GOL | 24.0536 | 24.0693 | 0.0157 | 0.5974 | 0.2237 | FALSE | new |
| P01721 | LV601 |  | Ig lambda chain V-VI region AR | 17.2274 | 17.1648 | -0.0626 | 0.5974 | 0.2237 | FALSE | new |
| Q9H6F2 | TM38A | TMEM38A | Trimeric intracellular cation channel type A | 17.0992 | 17.2241 | 0.1249 | 0.5974 | 0.2237 | FALSE | new |
| Q9Y5T5 | UBP16 | USP16 | Ubiquitin carboxyl-terminal hydrolase 16 | 17.3367 | 17.3778 | 0.0411 | 0.5974 | 0.2237 | FALSE | new |
| P61204 | ARF3;ARF1 | ARF3 | ADP-ribosylation factor 3;ADP-ribosylation factor 1 | 17.2278 | 17.2319 | 0.0041 | 0.6021 | 0.2203 | FALSE | new |
| P09622 | DLDH | DLD | Dihydrolipoyl dehydrogenase, mitochondrial | 20.0015 | 20.1334 | 0.132 | 0.6021 | 0.2203 | FALSE | new |
| O75636 | FCN3 | FCN3 | Ficolin-3 | 19.348 | 19.3441 | -0.0039 | 0.6021 | 0.2203 | FALSE | new |
| P01760 | HV105 |  | Ig heavy chain V-I region WOL | 15.7609 | 15.9921 | 0.2312 | 0.6021 | 0.2203 | FALSE | new |
| P04433 | KV309 |  | Ig kappa chain V-III region VG (Fragment) | 22.2817 | 22.2701 | -0.0116 | 0.6021 | 0.2203 | FALSE | new |
| O00533 | NCHL1 | CHL1 | Neural cell adhesion molecule L1-like protein | 17.5048 | 18.1303 | 0.6255 | 0.6021 | 0.2203 | FALSE | new |
| P23470 | PTPRG | PTPRG | Receptor-type tyrosine-protein phosphatase gamma | 18.8098 | 18.8584 | 0.0486 | 0.6021 | 0.2203 | FALSE | new |
| Q9UJT2 | TSKS | TSKS | Testis-specific serine kinase substrate | 20.1811 | 20.2464 | 0.0654 | 0.6021 | 0.2203 | FALSE | new |
| Q9P1F3 | ABRAL | ABRACL | Costars family protein ABRACL | 14.7122 | 13.5169 | -1.1953 | 0.6032 | 0.2195 | FALSE | new |
| P08519 | APOA | LPA | Apolipoprotein(a) | 18.9996 | 19.7723 | 0.7727 | 0.6032 | 0.2195 | FALSE | new |
| Q9H1Y0 | ATG5 | ATG5 | Autophagy protein 5 | 17.1333 | 17.1166 | -0.0167 | 0.6032 | 0.2195 | FALSE | new |
| Q03591 | FHR1 | CFHR1 | Complement factor H-related protein 1 | 19.0241 | 19.0354 | 0.0114 | 0.6032 | 0.2195 | FALSE | new |
| P01619 | KV301 |  | Ig kappa chain V-III region B6 | 19.7274 | 19.9112 | 0.1838 | 0.6032 | 0.2195 | FALSE | new |
| P01621 | KV303 |  | Ig kappa chain V-III region NG9 (Fragment) | 22.8962 | 22.9511 | 0.0548 | 0.6032 | 0.2195 | FALSE | new |
| Q9NP71 | MLXPL | MLXIPL | Carbohydrate-responsive element-binding protein | 19.2121 | 19.1777 | -0.0344 | 0.6032 | 0.2195 | FALSE | new |
| P30041 | PRDX6 | PRDX6 | Peroxiredoxin-6 | 15.4029 | 16.4031 | 1.0003 | 0.6032 | 0.2195 | FALSE | new |
| Q9BQE3 | TBA1C | TUBA1C | Tubulin alpha-1C chain | 17.4936 | 16.0074 | -1.4862 | 0.6032 | 0.2195 | FALSE | new |
| P35443 | TSP4 | THBS4 | Thrombospondin-4 | 15.8895 | 15.9988 | 0.1093 | 0.6032 | 0.2195 | FALSE | new |
| Q69YN4 | VIR | KIAA1429 | Protein virilizer homolog | 20.073 | 19.942 | -0.1311 | 0.6032 | 0.2195 | FALSE | new |
| P19652 | A1AG2 | ORM2 | Alpha-1-acid glycoprotein 2 | 23.9773 | 24.0126 | 0.0353 | 0.6035 | 0.2194 | FALSE | new |
| P02749 | APOH | APOH | Beta-2-glycoprotein 1 | 23.7007 | 23.782 | 0.0813 | 0.6035 | 0.2194 | FALSE | new |
| O75110 | ATP9A | ATP9A | Probable phospholipid-transporting ATPase IIA | 17.2623 | 16.877 | -0.3852 | 0.6035 | 0.2194 | FALSE | new |
| P25705 | ATPA | ATP5A1 | ATP synthase subunit alpha, mitochondrial | 17.1567 | 17.2659 | 0.1092 | 0.6035 | 0.2194 | FALSE | new |
| Q86UW7 | CAPS2 | CADPS2 | Calcium-dependent secretion activator 2 | 19.8617 | 19.9243 | 0.0625 | 0.6035 | 0.2194 | FALSE | new |
| Q9BXL7 | CAR11 | CARD11 | Caspase recruitment domain-containing protein 11 | 16.2324 | 16.1512 | -0.0812 | 0.6035 | 0.2194 | FALSE | new |
| P04040 | CATA | CAT | Catalase | 15.9843 | 15.9713 | -0.013 | 0.6035 | 0.2194 | FALSE | L |
| P61201 | CSN2 | COPS2 | COP9 signalosome complex subunit 2 | 15.6135 | 15.1394 | -0.474 | 0.6035 | 0.2194 | FALSE | L |
| Q9H4M9 | EHD1;EHD3 | EHD1 | EH domain-containing protein 1;EH domain-containing protein 3 | 14.4789 | 16.9577 | 2.4788 | 0.6035 | 0.2194 | FALSE | new |
| P05160 | F13B | F13B | Coagulation factor XIII B chain | 17.2849 | 17.3027 | 0.0178 | 0.6035 | 0.2194 | FALSE | new |
| P84243 | H33 | H3F3A | Histone H3.3 | 16.168 | 16.0633 | -0.1047 | 0.6035 | 0.2194 | FALSE | new |
| P01767 | HV306 |  | Ig heavy chain V-III region BUT | 19.293 | 19.2797 | -0.0133 | 0.6035 | 0.2194 | FALSE | new |
| P05019 | IGF1 | IGF1 | Insulin-like growth factor I | 16.4065 | 17.0176 | 0.6112 | 0.6035 | 0.2194 | FALSE | L |
| P01860 | IGHG3 | IGHG3 | Ig gamma-3 chain C region | 24.2452 | 24.2545 | 0.0094 | 0.6035 | 0.2194 | FALSE | new |
| P01591 | IGJ | IGJ | Immunoglobulin J chain | 21.9108 | 22.0025 | 0.0916 | 0.6035 | 0.2194 | FALSE | new |
| P01597 | KV105 |  | Ig kappa chain V-I region DEE | 18.6419 | 18.569 | -0.0729 | 0.6035 | 0.2194 | FALSE | new |
| P01598 | KV106 |  | Ig kappa chain V-I region EU | 22.5389 | 22.5867 | 0.0478 | 0.6035 | 0.2194 | FALSE | new |
| P01624 | KV306 |  | Ig kappa chain V-III region POM | 22.8214 | 22.8747 | 0.0533 | 0.6035 | 0.2194 | FALSE | new |
| P13473 | LAMP2 | LAMP2 | Lysosome-associated membrane glycoprotein 2 | 15.9419 | 15.7984 | -0.1435 | 0.6035 | 0.2194 | FALSE | new |
| Q14766 | LTBP1 | LTBP1 | Latent-transforming growth factor beta-binding protein 1 | 15.7106 | 15.7317 | 0.0211 | 0.6035 | 0.2194 | FALSE | new |
| P20774 | MIME | OGN | Mimecan | 14.9579 | 15.0947 | 0.1367 | 0.6035 | 0.2194 | FALSE | new |
| P13796 | PLSL | LCP1 | Plastin-2 | 17.0185 | 17.0565 | 0.038 | 0.6035 | 0.2194 | FALSE | new |
| P62937 | PPIA | PPIA | Peptidyl-prolyl cis-trans isomerase A | 15.8132 | 16.1186 | 0.3054 | 0.6035 | 0.2194 | FALSE | new |
| P41222 | PTGDS | PTGDS | Prostaglandin-H2 D-isomerase | 17.8418 | 17.6059 | -0.2358 | 0.6035 | 0.2194 | FALSE | L |
| Q6UX71 | PXDC2 | PLXDC2 | Plexin domain-containing protein 2 | 16.186 | 16.3893 | 0.2032 | 0.6035 | 0.2194 | FALSE | new |
| P20742 | PZP | PZP | Pregnancy zone protein | 19.658 | 16.9129 | -2.7451 | 0.6035 | 0.2194 | FALSE | new |
| Q99969 | RARR2 | RARRES2 | Retinoic acid receptor responder protein 2 | 14.6838 | 14.7578 | 0.074 | 0.6035 | 0.2194 | FALSE | new |
| P62424 | RL7A | RPL7A | 60S ribosomal protein L7a | 12.9318 | 12.6437 | -0.2881 | 0.6035 | 0.2194 | FALSE | new |
| Q9NWS8 | RMND1 | RMND1 | Required for meiotic nuclear division protein 1 homolog | 16.4825 | 16.1486 | -0.334 | 0.6035 | 0.2194 | FALSE | new |
| P49908 | SEPP1 | SEPP1 | Selenoprotein P | 18.3371 | 18.3084 | -0.0287 | 0.6035 | 0.2194 | FALSE | new |
| P12931 | SRC | SRC | Proto-oncogene tyrosine-protein kinase Src | 15.0019 | 15.2991 | 0.2972 | 0.6035 | 0.2194 | FALSE | new |
| P32856 | STX2 | STX2 | Syntaxin-2 | 15.5588 | 15.8857 | 0.3269 | 0.6035 | 0.2194 | FALSE | L |
| P55072 | TERA | VCP | Transitional endoplasmic reticulum ATPase | 16.278 | 16.3275 | 0.0494 | 0.6035 | 0.2194 | FALSE | new |
| P02786 | TFR1 | TFRC | Transferrin receptor protein 1 | 15.217 | 16.0412 | 0.8242 | 0.6035 | 0.2194 | FALSE | new |
| Q6XPS3 | TPTE2 | TPTE2 | Phosphatidylinositol 3,4,5-trisphosphate 3-phosphatase TPTE2 | 15.3379 | 15.4209 | 0.0831 | 0.6035 | 0.2194 | FALSE | new |
| O75083 | WDR1 | WDR1 | WD repeat-containing protein 1 | 16.6991 | 17.0299 | 0.3307 | 0.6035 | 0.2194 | FALSE | new |
| Q13790 | APOF | APOF | Apolipoprotein F | 18.2145 | 18.1852 | -0.0293 | 0.6091 | 0.2153 | FALSE | L |
| P00915 | CAH1 | CA1 | Carbonic anhydrase 1 | 16.9187 | 17.1069 | 0.1882 | 0.6091 | 0.2153 | FALSE | new |
| Q9ULV4 | COR1C | CORO1C | Coronin-1C | 17.0046 | 17.1228 | 0.1182 | 0.6091 | 0.2153 | FALSE | new |
| P01611 | KV119 |  | Ig kappa chain V-I region Wes | 20.981 | 20.9097 | -0.0713 | 0.6091 | 0.2153 | FALSE | new |
| Q99593 | TBX5 | TBX5 | T-box transcription factor TBX5 | 19.6888 | 19.8179 | 0.1291 | 0.6091 | 0.2153 | FALSE | new |
| P53804 | TTC3 | TTC3 | E3 ubiquitin-protein ligase TTC3 | 21.0663 | 20.9932 | -0.0731 | 0.6091 | 0.2153 | FALSE | new |
| O95497 | VNN1 | VNN1 | Pantetheinase | 19.2166 | 19.1634 | -0.0532 | 0.6091 | 0.2153 | FALSE | new |
| P09884 | DPOLA | POLA1 | DNA polymerase alpha catalytic subunit | 17.787 | 17.7358 | -0.0512 | 0.6147 | 0.2114 | FALSE | new |
| P00748 | FA12 | F12 | Coagulation factor XII | 20.1066 | 20.09 | -0.0166 | 0.6147 | 0.2114 | FALSE | new |
| Q92496 | FHR4 | CFHR4 | Complement factor H-related protein 4 | 16.8656 | 16.9362 | 0.0706 | 0.6147 | 0.2114 | FALSE | new |
| P01762 | HV301 |  | Ig heavy chain V-III region TRO | 22.7632 | 22.8048 | 0.0416 | 0.6147 | 0.2114 | FALSE | new |
| P01708 | LV205 |  | Ig lambda chain V-II region BUR | 17.9844 | 18.0218 | 0.0374 | 0.6147 | 0.2114 | FALSE | new |
| P07237 | PDIA1 | P4HB | Protein disulfide-isomerase | 15.6114 | 15.7518 | 0.1404 | 0.6147 | 0.2114 | FALSE | new |
| Q15942 | ZYX | ZYX | Zyxin | 16.2497 | 16.3419 | 0.0923 | 0.6147 | 0.2114 | FALSE | new |
| P59665 | DEF1;DEF3 | DEFA1 | Neutrophil defensin 1;Neutrophil defensin 3 | 17.9352 | 18.0141 | 0.0789 | 0.6169 | 0.2098 | FALSE | new |
| Q14697 | GANAB | GANAB | Neutral alpha-glucosidase AB | 14.2858 | 14.1227 | -0.1631 | 0.6169 | 0.2098 | FALSE | new |
| A6NJS3 | IV1U1 | IGHV1OR21-1 | Putative V-set and immunoglobulin domain-containing-like protein IGHV1OR21-1 | 14.7404 | 14.7563 | 0.016 | 0.6169 | 0.2098 | FALSE | new |
| P04208 | LV106 |  | Ig lambda chain V-I region WAH | 21.3555 | 21.4192 | 0.0637 | 0.6169 | 0.2098 | FALSE | new |
| P04209 | LV211 |  | Ig lambda chain V-II region NIG-84 | 19.4931 | 18.6342 | -0.8589 | 0.6169 | 0.2098 | FALSE | new |
| P01717 | LV403 |  | Ig lambda chain V-IV region Hil | 21.4122 | 21.371 | -0.0413 | 0.6169 | 0.2098 | FALSE | new |
| Q15166 | PON3 | PON3 | Serum paraoxonase/lactonase 3 | 17.1564 | 17.1077 | -0.0487 | 0.6169 | 0.2098 | FALSE | new |
| Q96QB1 | RHG07 | DLC1 | Rho GTPase-activating protein 7 | 20.0997 | 20.0779 | -0.0218 | 0.6169 | 0.2098 | FALSE | new |
| Q8IUB2 | WFDC3 | WFDC3 | WAP four-disulfide core domain protein 3 | 16.2719 | 16.3139 | 0.0421 | 0.6169 | 0.2098 | FALSE | L |
| O15060 | ZBT39 | ZBTB39 | Zinc finger and BTB domain-containing protein 39 | 21.9701 | 22.0838 | 0.1137 | 0.6169 | 0.2098 | FALSE | new |
| Q9H6X2 | ANTR1 | ANTXR1 | Anthrax toxin receptor 1 | 19.1259 | 18.9834 | -0.1425 | 0.6234 | 0.2052 | FALSE | new |
| P01780 | HV319 |  | Ig heavy chain V-III region JON | 19.1247 | 18.7017 | -0.4231 | 0.6234 | 0.2052 | FALSE | new |
| Q3ZCW2 | LEGL | LGALSL | Galectin-related protein | 15.4861 | 17.2149 | 1.7288 | 0.6234 | 0.2052 | FALSE | new |
| Q5VU43 | MYOME | PDE4DIP | Myomegalin | 16.4675 | 16.5839 | 0.1164 | 0.6234 | 0.2052 | FALSE | new |
| Q92736 | RYR2 | RYR2 | Ryanodine receptor 2 | 18.4886 | 18.2747 | -0.2139 | 0.6234 | 0.2052 | FALSE | new |
| Q9UK55 | ZPI | SERPINA10 | Protein Z-dependent protease inhibitor | 16.7446 | 16.739 | -0.0056 | 0.6234 | 0.2052 | FALSE | L |
| P55056 | APOC4 | APOC4 | Apolipoprotein C-IV | 17.8268 | 17.8213 | -0.0055 | 0.6276 | 0.2023 | FALSE | new |
| O75882 | ATRN | ATRN | Attractin | 18.9238 | 18.9425 | 0.0187 | 0.6276 | 0.2023 | FALSE | new |
| P47755 | CAZA2;CAZA1 | CAPZA2 | F-actin-capping protein subunit alpha-2;F-actin-capping protein subunit alpha-1 | 14.4102 | 14.2532 | -0.157 | 0.6276 | 0.2023 | FALSE | new |
| P02741 | CRP | CRP | C-reactive protein | 15.1089 | 15.7749 | 0.666 | 0.6276 | 0.2023 | FALSE | L |
| P24593 | IBP5 | IGFBP5 | Insulin-like growth factor-binding protein 5 | 13.9608 | 13.7849 | -0.176 | 0.6276 | 0.2023 | FALSE | new |
| P83593 | KV405 |  | Ig kappa chain V-IV region STH (Fragment) | 18.24 | 18.2133 | -0.0268 | 0.6276 | 0.2023 | FALSE | new |
| Q96QR1 | SG3A1 | SCGB3A1 | Secretoglobin family 3A member 1 | 14.6429 | 14.4624 | -0.1805 | 0.6276 | 0.2023 | FALSE | new |
| P37837 | TALDO | TALDO1 | Transaldolase | 18.1045 | 17.9485 | -0.156 | 0.6276 | 0.2023 | FALSE | new |
| P15144 | AMPN | ANPEP | Aminopeptidase N | 15.5897 | 15.0982 | -0.4914 | 0.6307 | 0.2002 | FALSE | new |
| P02656 | APOC3 | APOC3 | Apolipoprotein C-III | 23.5581 | 23.6071 | 0.049 | 0.6307 | 0.2002 | FALSE | new |
| O00299 | CLIC1 | CLIC1 | Chloride intracellular channel protein 1 | 16.9875 | 17.0301 | 0.0427 | 0.6307 | 0.2002 | FALSE | new |
| Q9UP83 | COG5 | COG5 | Conserved oligomeric Golgi complex subunit 5 | 19.3824 | 19.4006 | 0.0182 | 0.6307 | 0.2002 | FALSE | new |
| P06314 | KV404 |  | Ig kappa chain V-IV region B17 | 13.5256 | 13.6665 | 0.1408 | 0.6307 | 0.2002 | FALSE | new |
| P04180 | LCAT | LCAT | Phosphatidylcholine-sterol acyltransferase | 16.9831 | 17.0286 | 0.0455 | 0.6307 | 0.2002 | FALSE | new |
| Q15555 | MARE2 | MAPRE2 | Microtubule-associated protein RP/EB family member 2 | 14.0569 | 14.2965 | 0.2396 | 0.6307 | 0.2002 | FALSE | new |
| Q92878 | RAD50 | RAD50 | DNA repair protein RAD50 | 18.6547 | 18.5335 | -0.1212 | 0.6307 | 0.2002 | FALSE | new |
| P60174 | TPIS | TPI1 | Triosephosphate isomerase | 16.7687 | 15.9057 | -0.863 | 0.6307 | 0.2002 | FALSE | new |
| Q18PE1 | DOK7 | DOK7 | Protein Dok-7 | 16.388 | 16.5746 | 0.1866 | 0.6327 | 0.1988 | FALSE | new |
| P62805 | H4 | HIST1H4A | Histone H4 | 15.9723 | 15.6269 | -0.3454 | 0.6327 | 0.1988 | FALSE | new |
| Q14520 | HABP2 | HABP2 | Hyaluronan-binding protein 2 | 17.4076 | 17.4373 | 0.0297 | 0.6327 | 0.1988 | FALSE | new |
| Q9Y4D8 | HECD4 | HECTD4 | Probable E3 ubiquitin-protein ligase HECTD4 | 22.6518 | 22.6149 | -0.0368 | 0.6327 | 0.1988 | FALSE | new |
| P05362 | ICAM1 | ICAM1 | Intercellular adhesion molecule 1 | 15.9783 | 15.8901 | -0.0881 | 0.6327 | 0.1988 | FALSE | new |
| P0CG05 | LAC2 | IGLC2 | Ig lambda-2 chain C regions | 26.5948 | 26.5893 | -0.0055 | 0.6327 | 0.1988 | FALSE | new |
| O75019 | LIRA1;LIRA3;LIRB1 | LILRA1 | Leukocyte immunoglobulin-like receptor subfamily A member 1;Leukocyte immunoglobulin-like receptor subfamily A member 3;Leukocyte immunoglobulin-like receptor subfamily B member 1 | 15.2447 | 18.3506 | 3.1059 | 0.6327 | 0.1988 | FALSE | new |
| P36955 | PEDF | SERPINF1 | Pigment epithelium-derived factor | 20.2815 | 20.2896 | 0.0081 | 0.6327 | 0.1988 | FALSE | L |
| P04278 | SHBG | SHBG | Sex hormone-binding globulin | 17.6659 | 17.2451 | -0.4208 | 0.6327 | 0.1988 | FALSE | new |
| Q66K66 | TM198 | TMEM198 | Transmembrane protein 198 | 24.2589 | 24.2167 | -0.0423 | 0.6327 | 0.1988 | FALSE | new |
| P11597 | CETP | CETP | Cholesteryl ester transfer protein | 17.5135 | 17.8309 | 0.3174 | 0.6419 | 0.1925 | FALSE | new |
| P22792 | CPN2 | CPN2 | Carboxypeptidase N subunit 2 | 18.2575 | 18.2982 | 0.0406 | 0.6419 | 0.1925 | FALSE | new |
| P01601 | KV109 | IGKV1D-16 | Ig kappa chain V-ID region 16 (Fragment) | 18.7138 | 18.4631 | -0.2507 | 0.6419 | 0.1925 | FALSE | new |
| P03950 | ANGI | ANG | Angiogenin | 16.6627 | 16.644 | -0.0186 | 0.6489 | 0.1878 | FALSE | new |
| P10809 | CH60 | HSPD1 | 60 kDa heat shock protein, mitochondrial | 10.7493 | 10.7725 | 0.0231 | 0.6489 | 0.1878 | FALSE | new |
| Q9H4G4 | GAPR1 | GLIPR2 | Golgi-associated plant pathogenesis-related protein 1 | 13.2029 | 12.8131 | -0.3897 | 0.6489 | 0.1878 | FALSE | new |
| P24592 | IBP6 | IGFBP6 | Insulin-like growth factor-binding protein 6 | 14.4725 | 14.4754 | 0.0028 | 0.6489 | 0.1878 | FALSE | L.T |
| Q99832 | TCPH | CCT7 | T-complex protein 1 subunit eta | 12.3143 | 13.2628 | 0.9485 | 0.6489 | 0.1878 | FALSE | new |

# **Supplementary Table 3. Results of the Small-seq.**

| **#** | **ID** | **name** | **log2(mean basal expression)** | **log2(fold change)** | **standard error** | **FDR** | **-log10(FDR)** |
| --- | --- | --- | --- | --- | --- | --- | --- |
| 1 | MIMAT0000727 | miR-374a-5p | 3.59689729 | -0.0334011 | 0.003983298 | 2.83369E-14 | 13.54764829 |
| 2 | MIMAT0000083 | miR-26b-5p | 6.076805901 | -0.031344874 | 0.003870306 | 1.55397E-13 | 12.80855614 |
| 3 | MIMAT0000440 | miR-191-5p | 7.746023825 | -0.03332939 | 0.004200156 | 3.92055E-13 | 12.40665292 |
| 4 | MIMAT0000693 | miR-30e-3p | 3.562678406 | -0.04047093 | 0.005647295 | 9.34293E-11 | 10.02951689 |
| 5 | MIMAT0000082_1 | miR-26a-5p | 8.971925829 | -0.030800728 | 0.004304533 | 9.34293E-11 | 10.02951689 |
| 6 | MIMAT0000444 | miR-126-5p | 7.897076257 | -0.024132894 | 0.003644839 | 3.32737E-09 | 8.477898504 |
| 7 | MIMAT0000231_1 | miR-199a-5p | 7.826693692 | -0.040318012 | 0.006166121 | 4.96663E-09 | 8.303938311 |
| 8 | MIMAT0004955 | miR-374b-5p | 2.469950421 | -0.036151587 | 0.00559425 | 7.2198E-09 | 8.141474567 |
| 9 | MIMAT0002809 | miR-146b-5p | 4.101139508 | -0.035165657 | 0.005537966 | 1.34041E-08 | 7.872762311 |
| 10 | MIMAT0004697 | miR-151a-5p | 6.126626433 | -0.026648572 | 0.004260914 | 2.23766E-08 | 7.650206055 |
| 11 | MIMAT0003297 | miR-628-3p | 3.734760867 | -0.02779072 | 0.004489927 | 3.07126E-08 | 7.51268386 |
| 12 | MIMAT0004945 | miR-744-5p | 5.485079696 | -0.026889928 | 0.00439055 | 4.24518E-08 | 7.372103618 |
| 13 | MIMAT0000093 | miR-93-5p | 5.113673306 | -0.0286918 | 0.004822284 | 1.15623E-07 | 6.936955507 |
| 14 | MIMAT0000688 | miR-301a-3p | 0.855452384 | -0.052643206 | 0.008899597 | 1.32561E-07 | 6.877584913 |
| 15 | MIMAT0000417 | miR-15b-5p | 3.993890558 | -0.026176495 | 0.0045344 | 2.90951E-07 | 6.536179949 |
| 16 | MIMAT0000419 | miR-27b-3p | 7.961646328 | -0.021964268 | 0.00382293 | 3.02082E-07 | 6.519875148 |
| 17 | MIMAT0002174 | miR-484 | 6.363011906 | -0.022947447 | 0.003991025 | 3.02082E-07 | 6.519875148 |
| 18 | MIMAT0000757 | miR-151a-3p | 6.954182347 | -0.029337868 | 0.005152273 | 3.65696E-07 | 6.43687967 |
| 19 | MIMAT0000415 | let-7i-5p | 6.848791655 | -0.026735314 | 0.004695324 | 3.65696E-07 | 6.43687967 |
| 20 | MIMAT0016895 | miR-2355-5p | 1.224670082 | -0.031610999 | 0.005573972 | 3.97088E-07 | 6.401113267 |
| 21 | MIMAT0003322 | miR-652-3p | 6.042743593 | -0.023258935 | 0.004138566 | 5.09081E-07 | 6.293212726 |
| 22 | MIMAT0004502 | miR-28-3p | 4.796737369 | -0.028366239 | 0.005056367 | 5.15003E-07 | 6.28819032 |
| 23 | MIMAT0000414 | let-7g-5p | 4.7932069 | -0.029759742 | 0.005367081 | 7.16263E-07 | 6.144927359 |
| 24 | MIMAT0000617 | miR-200c-3p | 0.58881468 | -0.035056283 | 0.006371598 | 8.76378E-07 | 6.057308523 |
| 25 | MIMAT0000443 | miR-125a-5p | 5.420517911 | -0.019333536 | 0.003537973 | 9.99394E-07 | 6.000263291 |
| 26 | MIMAT0000279 | miR-222-3p | 4.608652288 | -0.02761355 | 0.005050398 | 9.99394E-07 | 6.000263291 |
| 27 | MIMAT0001343 | miR-425-3p | 5.126625666 | -0.026032545 | 0.004772974 | 1.02057E-06 | 5.9911573 |
| 28 | MIMAT0000281 | miR-224-5p | 3.61029723 | -0.033090843 | 0.006093188 | 1.12208E-06 | 5.949974491 |
| 29 | MIMAT0003294 | miR-625-5p | 3.424055959 | -0.03543651 | 0.006585637 | 1.43135E-06 | 5.84425541 |
| 30 | MIMAT0000751 | miR-330-3p | 2.754683976 | -0.029745013 | 0.005560436 | 1.60872E-06 | 5.7935207 |
| 31 | MIMAT0005797 | miR-1301-3p | 3.182622055 | -0.02597258 | 0.00485673 | 1.60872E-06 | 5.7935207 |
| 32 | MIMAT0000646 | miR-155-5p | 1.500384754 | -0.031964762 | 0.006037088 | 2.08585E-06 | 5.680717007 |
| 33 | MIMAT0000433 | miR-142-5p | 7.778954341 | -0.018542715 | 0.003510381 | 2.16544E-06 | 5.664453737 |
| 34 | MIMAT0001340 | miR-423-3p | 5.483395658 | -0.024980389 | 0.004785646 | 2.94925E-06 | 5.530288648 |
| 35 | MIMAT0000072 | miR-18a-5p | 3.390125438 | -0.028132233 | 0.005416701 | 3.30008E-06 | 5.481475165 |
| 36 | MIMAT0002821 | miR-181d-5p | 0.027625255 | -0.040932376 | 0.007890839 | 3.31849E-06 | 5.479059075 |
| 37 | MIMAT0000078 | miR-23a-3p | 7.707909795 | -0.01585824 | 0.003066215 | 3.50669E-06 | 5.455103181 |
| 38 | MIMAT0000445 | miR-126-3p | 7.626108273 | -0.018274552 | 0.003576146 | 4.74448E-06 | 5.32381114 |
| 39 | MIMAT0000245 | miR-30d-5p | 8.327903856 | -0.02248808 | 0.004450424 | 6.24443E-06 | 5.204507252 |
| 40 | MIMAT0002816 | miR-494-3p | 1.43246199 | -0.037660346 | 0.007553051 | 8.62497E-06 | 5.064242634 |
| 41 | MIMAT0004552 | miR-139-3p | 0.381763644 | -0.038756053 | 0.007798296 | 9.15372E-06 | 5.038402455 |
| 42 | MIMAT0000258 | miR-181c-5p | -0.870644431 | -0.04852265 | 0.009788965 | 9.55159E-06 | 5.019924109 |
| 43 | MIMAT0004559 | miR-181c-3p | 1.228410196 | -0.034597418 | 0.007031508 | 1.12512E-05 | 4.9487999 |
| 44 | MIMAT0000420 | miR-30b-5p | 1.305371033 | -0.028688475 | 0.005919672 | 1.60095E-05 | 4.795622669 |
| 45 | MIMAT0000760 | miR-331-3p | 0.806573675 | -0.035049098 | 0.00724094 | 1.61247E-05 | 4.792509256 |
| 46 | MIMAT0004597 | miR-140-3p | 7.043803897 | -0.018276306 | 0.003803055 | 1.87744E-05 | 4.726433529 |
| 47 | MIMAT0027604 | miR-6852-5p | 1.739537711 | -0.028271012 | 0.005890184 | 1.89348E-05 | 4.722738995 |
| 48 | MIMAT0002813 | miR-493-5p | 0.797485888 | -0.04203165 | 0.008831122 | 2.26428E-05 | 4.6450699 |
| 49 | MIMAT0000070 | miR-17-5p | 2.465893886 | -0.031513218 | 0.006629824 | 2.28767E-05 | 4.640605889 |
| 50 | MIMAT0000101_1 | miR-103a-3p | 5.173028757 | -0.023262927 | 0.004900234 | 2.30877E-05 | 4.63661852 |
| 51 | MIMAT0000254 | miR-10b-5p | 5.5724133 | 0.01721227 | 0.003636135 | 2.4211E-05 | 4.615986388 |
| 52 | MIMAT0000256_1 | miR-181a-5p | 5.346577185 | -0.017780141 | 0.003780744 | 2.76336E-05 | 4.558562246 |
| 53 | MIMAT0000689 | miR-99b-5p | 5.344088805 | -0.019323786 | 0.004113096 | 2.77441E-05 | 4.556829355 |
| 54 | MIMAT0004813 | miR-411-3p | 0.244156924 | -0.035736286 | 0.007632497 | 2.94435E-05 | 4.531010346 |
| 55 | MIMAT0000063 | let-7b-5p | 6.107379787 | -0.020057446 | 0.004287529 | 2.94824E-05 | 4.530436973 |
| 56 | MIMAT0000067_1 | let-7f-5p | 5.624232328 | -0.019148642 | 0.004122693 | 3.40583E-05 | 4.467776935 |
| 57 | MIMAT0018965 | miR-4446-3p | 0.485701284 | -0.039045648 | 0.008498119 | 4.18584E-05 | 4.378216892 |
| 58 | MIMAT0000692 | miR-30e-5p | 7.721117319 | -0.022354318 | 0.004865313 | 4.18584E-05 | 4.378216892 |
| 59 | MIMAT0004692 | miR-340-5p | 4.604085597 | -0.026926395 | 0.005873105 | 4.31532E-05 | 4.364986853 |
| 60 | MIMAT0000759 | miR-148b-3p | 5.515724893 | -0.018831418 | 0.004113355 | 4.37902E-05 | 4.358622783 |
| 61 | MIMAT0000084 | miR-27a-3p | 6.829768015 | -0.01891912 | 0.004162133 | 4.94932E-05 | 4.305454143 |
| 62 | tRF-40-2R1HPSR9O9337KB6 | tRF-40-2R1HPSR9O9337KB6 | -0.065784371 | 0.053431343 | 0.011754105 | 4.94932E-05 | 4.305454143 |
| 63 | trna3-GluTTC_1 | trna3-GluTTC | 3.698106362 | 0.037803405 | 0.008463518 | 6.84884E-05 | 4.164382703 |
| 64 | MIMAT0000435 | miR-143-3p | 5.270402503 | -0.027087961 | 0.006064644 | 6.84884E-05 | 4.164382703 |
| 65 | MIMAT0003239 | miR-574-3p | 1.99048579 | -0.028661651 | 0.006410887 | 6.84884E-05 | 4.164382703 |
| 66 | MIMAT0004801 | miR-590-3p | 1.236163714 | -0.032472111 | 0.007338485 | 8.18651E-05 | 4.086901262 |
| 67 | MIMAT0004703 | miR-335-3p | 2.326895323 | -0.030706503 | 0.007039859 | 0.000107815 | 3.967321424 |
| 68 | MIMAT0007881 | miR-1908-5p | 2.966646499 | -0.023483241 | 0.005401237 | 0.000113268 | 3.945892906 |
| 69 | MIMAT0000456 | miR-186-5p | 5.073688843 | -0.025420831 | 0.005896185 | 0.000131658 | 3.880552391 |
| 70 | MIMAT0004500 | miR-26b-3p | 1.499401491 | -0.026555532 | 0.00617129 | 0.000134754 | 3.870458401 |
| 71 | MIMAT0000753 | miR-342-3p | 4.203398328 | -0.02314505 | 0.00539084 | 0.000138781 | 3.857668887 |
| 72 | MIMAT0000763 | miR-338-3p | 0.109540903 | -0.034649622 | 0.00808061 | 0.000140227 | 3.853166933 |
| 73 | MIMAT0000680 | miR-106b-5p | 3.489042951 | -0.027787552 | 0.006499391 | 0.000146138 | 3.835235685 |
| 74 | tRF-40-2VR008R959KUMKF6 | tRF-40-2VR008R959KUMKF6 | 2.245153044 | 0.030674587 | 0.007179187 | 0.000146138 | 3.835235685 |
| 75 | trna6-ValCAC_1 | trna6-ValCAC | 2.884243447 | -0.021212692 | 0.004987978 | 0.000155583 | 3.808038583 |
| 76 | MIMAT0019731 | miR-4662a-5p | -0.076640137 | -0.035204511 | 0.008273139 | 0.000155583 | 3.808038583 |
| 77 | MIMAT0000280 | miR-223-3p | 5.540970656 | -0.031469998 | 0.007425277 | 0.00016385 | 3.785553647 |
| 78 | MIMAT0000096 | miR-98-5p | 2.909666231 | -0.026160074 | 0.00619148 | 0.000171415 | 3.765950287 |
| 79 | MIMAT0004984_4 | miR-941 | 0.979152288 | -0.030351621 | 0.007190908 | 0.000172538 | 3.763115914 |
| 80 | MIMAT0000761 | miR-324-5p | 1.267630481 | -0.027790269 | 0.006608063 | 0.000180095 | 3.74449748 |
| 81 | tRF-18-BS68BFD2 | tRF-18-BS68BFD2 | 1.775310821 | -0.033739687 | 0.008022593 | 0.000180095 | 3.74449748 |
| 82 | MIMAT0000066 | let-7e-5p | 0.913647835 | -0.028126164 | 0.00670723 | 0.000187663 | 3.726621116 |
| 83 | MIMAT0000446 | miR-127-3p | 4.591435429 | -0.03025861 | 0.007248305 | 0.000201425 | 3.695887383 |
| 84 | MIMAT0000438 | miR-152-3p | 2.721784236 | -0.021286996 | 0.005104556 | 0.000202892 | 3.692734051 |
| 85 | MIMAT0017950 | miR-2355-3p | 0.359371357 | -0.033056166 | 0.007956635 | 0.000214738 | 3.668091518 |
| 86 | MIMAT0000071 | miR-17-3p | 0.964586083 | -0.027927861 | 0.006762642 | 0.0002365 | 3.626169701 |
| 87 | MIMAT0004682 | miR-361-3p | 2.006708673 | -0.029423024 | 0.007129414 | 0.000236578 | 3.626024809 |
| 88 | MIMAT0003886 | miR-769-5p | 1.561498569 | -0.0284019 | 0.006897444 | 0.000243474 | 3.613547652 |
| 89 | MIMAT0002178 | miR-487a-3p | -0.543840495 | -0.039396853 | 0.009577388 | 0.000245159 | 3.610552438 |
| 90 | MIMAT0004563 | miR-199b-3p | 5.048864084 | -0.023659387 | 0.005757279 | 0.000245159 | 3.610552438 |
| 91 | MIMAT0002814 | miR-432-5p | 2.966557667 | -0.027721708 | 0.006754215 | 0.000245159 | 3.610552438 |
| 92 | MIMAT0000232_1 | miR-199a-3p | 6.057706114 | -0.023455891 | 0.005716222 | 0.000245159 | 3.610552438 |
| 93 | tRF-34-PV6RRNLNK88KHR | tRF-34-PV6RRNLNK88KHR | 1.465184603 | 0.091830221 | 0.022374848 | 0.000245159 | 3.610552438 |
| 94 | MIMAT0004568 | miR-221-5p | 1.578490299 | -0.026015117 | 0.006388845 | 0.000277732 | 3.556374278 |
| 95 | MIMAT0000449 | miR-146a-5p | 8.530630824 | -0.015247498 | 0.003800271 | 0.000354582 | 3.45028371 |
| 96 | MIMAT0000424_1 | miR-128-3p | 4.675047509 | -0.022067541 | 0.005519031 | 0.000371934 | 3.429534186 |
| 97 | MIMAT0000227 | miR-197-3p | 1.712777092 | -0.022452007 | 0.005619705 | 0.000373132 | 3.428137435 |
| 98 | MIMAT0003393 | miR-425-5p | 5.044326417 | -0.016942119 | 0.004252892 | 0.000387766 | 3.411430473 |
| 99 | MIMAT0000065 | let-7d-5p | 2.983387953 | -0.021709536 | 0.005496982 | 0.000436457 | 3.360058381 |
| 100 | MIMAT0000243 | miR-148a-3p | 6.997398348 | -0.014395204 | 0.003643785 | 0.000436457 | 3.360058381 |
| 101 | MIMAT0004494 | miR-21-3p | 3.906279628 | -0.018660052 | 0.004726134 | 0.000436457 | 3.360058381 |
| 102 | MIMAT0003161 | miR-493-3p | 0.138610645 | -0.035370497 | 0.008987169 | 0.000455501 | 3.341510439 |
| 103 | MIMAT0004680 | miR-130b-5p | 1.589971709 | -0.024228706 | 0.006166162 | 0.00046183 | 3.335517676 |
| 104 | tRF-34-JJ6RRNLIK898HR | tRF-34-JJ6RRNLIK898HR | 1.209212325 | 0.080166947 | 0.020410752 | 0.00046183 | 3.335517676 |
| 105 | MIMAT0000756 | miR-326 | 1.848652087 | -0.028417553 | 0.00726272 | 0.000486576 | 3.312849082 |
| 106 | MIMAT0003339 | miR-421 | 2.09959952 | -0.020669074 | 0.005293256 | 0.000498219 | 3.302579876 |
| 107 | MIMAT0002172 | miR-376b-3p | 0.012942803 | -0.033070631 | 0.008477083 | 0.00050099 | 3.300171151 |
| 108 | MIMAT0000278 | miR-221-3p | 5.996015766 | -0.025791883 | 0.006643996 | 0.000537225 | 3.269843411 |
| 109 | MIMAT0030414 | miR-4433b-3p | 1.534951908 | -0.029936996 | 0.007725116 | 0.000547165 | 3.26188197 |
| 110 | MIMAT0010214 | miR-151b | 2.777719474 | -0.01313307 | 0.003400853 | 0.000573243 | 3.241660897 |
| 111 | MIMAT0004611 | miR-185-3p | 0.801681105 | -0.035898649 | 0.009365994 | 0.000638987 | 3.194508309 |
| 112 | MIMAT0001412 | miR-18b-5p | 0.454597971 | -0.027555675 | 0.007197265 | 0.000644289 | 3.190919099 |
| 113 | MIMAT0003180 | miR-487b-3p | 2.955445197 | -0.028302845 | 0.007402876 | 0.000652765 | 3.185242994 |
| 114 | MIMAT0000080_1 | miR-24-3p | 5.830721034 | -0.0199565 | 0.005255641 | 0.000718983 | 3.143281668 |
| 115 | MIMAT0000431 | miR-140-5p | 0.808617224 | -0.035800262 | 0.009576318 | 0.000901762 | 3.044908088 |
| 116 | MIMAT0000703 | miR-361-5p | 5.356305968 | -0.014601205 | 0.003911147 | 0.000912611 | 3.039714241 |
| 117 | MIMAT0004505 | miR-32-3p | -0.613552878 | -0.032847708 | 0.008808267 | 0.000919433 | 3.036480036 |
| 118 | MIMAT0001618 | miR-191-3p | 0.92360413 | -0.025282625 | 0.006816894 | 0.000988203 | 3.005153908 |
| 119 | MIMAT0022709 | miR-652-5p | 0.443956324 | -0.027120553 | 0.007337897 | 0.001030871 | 2.986795733 |
| 120 | trna55-IleTAT_1 | trna55-IleTAT | 2.767241969 | -0.016831388 | 0.004573946 | 0.00108911 | 2.962928273 |
| 121 | MIMAT0002817 | miR-495-3p | 2.815792103 | -0.02585893 | 0.007061214 | 0.001157711 | 2.936399905 |
| 122 | MIMAT0005796 | miR-1271-5p | 0.618059521 | -0.027191802 | 0.007457944 | 0.001222519 | 2.912744367 |
| 123 | MIMAT0022842 | miR-98-3p | 0.77718681 | -0.026397941 | 0.00726824 | 0.001280621 | 2.892579241 |
| 124 | MIMAT0003249 | miR-584-5p | 6.347710874 | -0.016053164 | 0.004423664 | 0.001285287 | 2.890999895 |
| 125 | MIMAT0000752 | miR-328-3p | 2.73411819 | -0.023058021 | 0.006417159 | 0.001463454 | 2.834620974 |
| 126 | MIMAT0004819 | miR-671-3p | 0.389808287 | -0.028427014 | 0.007968527 | 0.001602333 | 2.795247279 |
| 127 | MIMAT0005951 | miR-1307-3p | 3.581986835 | -0.026199656 | 0.007405918 | 0.001780016 | 2.749576004 |
| 128 | MIMAT0001639 | miR-409-3p | 4.064220011 | -0.023427537 | 0.006660368 | 0.001906225 | 2.719825948 |
| 129 | MIMAT0000455 | miR-185-5p | 7.089601355 | -0.017306419 | 0.004964367 | 0.002127419 | 2.672147066 |
| 130 | MIMAT0005878 | miR-1287-5p | -0.147676748 | -0.028983616 | 0.008324391 | 0.00214571 | 2.668428988 |
| 131 | MIMAT0000736 | miR-381-3p | 2.345058334 | -0.025200788 | 0.00725415 | 0.002192101 | 2.659139402 |
| 132 | MIMAT0005948 | miR-664a-5p | 1.46343456 | -0.018626167 | 0.005416827 | 0.002472966 | 2.606781899 |
| 133 | MIMAT0004614 | miR-193a-5p | 0.879670245 | 0.028961507 | 0.008425413 | 0.002472966 | 2.606781899 |
| 134 | MIMAT0000451 | miR-150-5p | 4.623921684 | -0.027875754 | 0.008114725 | 0.00247446 | 2.606519498 |
| 135 | MIMAT0000425 | miR-130a-3p | 6.269685746 | -0.012928089 | 0.003783489 | 0.002626651 | 2.580597639 |
| 136 | MIMAT0000418 | miR-23b-3p | 4.178184902 | -0.012114721 | 0.003561841 | 0.002760682 | 2.558983556 |
| 137 | MIMAT0004954 | miR-543 | 1.382567568 | -0.026677865 | 0.007847856 | 0.002760682 | 2.558983556 |
| 138 | MIMAT0000099_1 | miR-101-3p | 5.726165844 | -0.017098182 | 0.005118616 | 0.003394909 | 2.469171856 |
| 139 | trna84-GluTTC_1 | trna84-GluTTC | -0.547663165 | 0.055245218 | 0.0165707 | 0.003450021 | 2.462178239 |
| 140 | MIMAT0014982 | miR-3120-3p | 0.210124089 | -0.023605034 | 0.007095634 | 0.003515256 | 2.454043021 |
| 141 | trna3-ProTGG_1 | trna3-ProTGG_1 | 3.544215899 | 0.017706702 | 0.005409679 | 0.004224041 | 2.374271853 |
| 142 | MIMAT0002176 | miR-485-3p | 0.457356617 | -0.033507514 | 0.010307211 | 0.004537109 | 2.343220781 |
| 143 | MIMAT0004481_1 | let-7a-3p | 1.297249844 | -0.027126093 | 0.008372605 | 0.004683132 | 2.329463599 |
| 144 | trna134-GluTTC_1 | trna134-GluTTC | -0.541271548 | 0.053884492 | 0.016643371 | 0.004687738 | 2.329036692 |
| 145 | trna4-AspGTC_1 | trna4-AspGTC | 1.434151498 | 0.019054726 | 0.005921542 | 0.004987798 | 2.302091138 |
| 146 | MIMAT0004985 | miR-942-5p | 0.309876622 | -0.022977087 | 0.007149723 | 0.005026003 | 2.29877724 |
| 147 | tRF-18-ODMJ6BD1 | tRF-18-ODMJ6BD1 | 0.138793219 | 0.046697406 | 0.014557672 | 0.005096254 | 2.292748933 |
| 148 | trna81-AspGTC_1 | trna81-AspGTC | 1.436892008 | 0.018864118 | 0.005898765 | 0.005236575 | 2.280952667 |
| 149 | MIMAT0000062_2 | let-7a-5p | 5.566043473 | -0.012299413 | 0.003854896 | 0.005336168 | 2.272770516 |
| 150 | trna69-AspGTC_1 | trna69-AspGTC | 1.429231102 | 0.018851109 | 0.005927606 | 0.005457654 | 2.26299403 |
| 151 | trna78-AspGTC_1 | trna78-AspGTC | 1.429231102 | 0.018851109 | 0.005927606 | 0.005457654 | 2.26299403 |
| 152 | trna75-AspGTC_1 | trna75-AspGTC | 1.428141783 | 0.018737902 | 0.005935582 | 0.005839189 | 2.233647455 |
| 153 | MIMAT0004551 | miR-30d-3p | -0.159881829 | -0.025137219 | 0.007962992 | 0.005839189 | 2.233647455 |
| 154 | MIMAT0000750 | miR-340-3p | -0.499209539 | -0.029027661 | 0.009240852 | 0.006117767 | 2.213407058 |
| 155 | trna72-AspGTC_1 | trna72-AspGTC | 1.430893723 | 0.018547965 | 0.005912742 | 0.006167822 | 2.209868178 |
| 156 | MIMAT0000434 | miR-142-3p | 1.055484347 | -0.023078338 | 0.007373826 | 0.006280128 | 2.202031515 |
| 157 | MIMAT0004921 | miR-889-3p | 2.718109393 | -0.020024726 | 0.006423137 | 0.006503647 | 2.186843034 |
| 158 | MIMAT0003266 | miR-598-3p | -0.172588439 | -0.02936517 | 0.00954051 | 0.007387495 | 2.131502804 |
| 159 | MIMAT0004484 | let-7d-3p | 5.220037464 | -0.01511736 | 0.004919386 | 0.007463352 | 2.127066067 |
| 160 | MIMAT0002876 | miR-505-3p | -0.085978705 | -0.025425369 | 0.00835679 | 0.008212302 | 2.085535093 |
| 161 | MIMAT0000085 | miR-28-5p | -1.200274276 | -0.036329616 | 0.011963461 | 0.008295748 | 2.081144443 |
| 162 | MIMAT0004509 | miR-93-3p | -0.583058456 | -0.029246057 | 0.009634065 | 0.008295748 | 2.081144443 |
| 163 | MIMAT0000764 | miR-339-5p | 0.589102129 | -0.032125812 | 0.01065288 | 0.008808592 | 2.055093494 |
| 164 | MIMAT0000076 | miR-21-5p | 6.71914357 | -0.015087008 | 0.005048842 | 0.009581984 | 2.018544569 |
| 165 | MIMAT0000075 | miR-20a-5p | 3.207147804 | -0.019173616 | 0.006421285 | 0.009594727 | 2.017967384 |
| 166 | MIMAT0000691 | miR-130b-3p | 4.309478755 | -0.010156896 | 0.003409881 | 0.00976648 | 2.010261932 |
| 167 | MIMAT0000257_1 | miR-181b-5p | 2.010630754 | -0.017923163 | 0.006022939 | 0.009798638 | 2.008834274 |
| 168 | MIMAT0002175 | miR-485-5p | -0.564887473 | -0.028982097 | 0.009853082 | 0.010890215 | 1.962963558 |
| 169 | MIMAT0004761 | miR-483-5p | 0.509476246 | 0.026093713 | 0.008892931 | 0.011080692 | 1.955433114 |
| 170 | trna11-GluTTC_1 | trna11-GluTTC | 1.765100223 | 0.016181745 | 0.005573641 | 0.012149436 | 1.915443871 |
| 171 | tRF-19-B1RHODE2 | tRF-19-B1RHODE2 | 0.274351595 | -0.025910307 | 0.008928959 | 0.012149436 | 1.915443871 |
| 172 | MIMAT0000318 | miR-200b-3p | -0.450201832 | -0.027670511 | 0.009556849 | 0.012330574 | 1.909016712 |
| 173 | MIMAT0022838 | miR-1185-1-3p | -0.416364058 | -0.029847411 | 0.010364077 | 0.012877173 | 1.890179459 |
| 174 | MIMAT0000088 | miR-30a-3p | 0.106051627 | -0.022795491 | 0.007928117 | 0.01299199 | 1.886324321 |
| 175 | trna7-GlnCTG_1 | trna7-GlnCTG | 1.198958264 | -0.016532308 | 0.005889299 | 0.015904311 | 1.798485136 |
| 176 | MIMAT0004700 | miR-331-5p | 2.185067572 | -0.013131123 | 0.004677774 | 0.015904311 | 1.798485136 |
| 177 | MIMAT0004949 | miR-877-5p | 1.897947126 | -0.015530768 | 0.005604292 | 0.017668603 | 1.752797781 |
| 178 | trna83-LeuTAA_1 | trna83-LeuTAA | 2.378051552 | -0.012219613 | 0.004431233 | 0.018318159 | 1.737118176 |
| 179 | MIMAT0022727 | miR-1307-5p | 1.764137177 | -0.018000646 | 0.006540736 | 0.018526165 | 1.732214472 |
| 180 | trna13-LysCTT_2 | trna13-LysCTT_1 | 1.499185701 | 0.020718067 | 0.007541937 | 0.018708429 | 1.727962691 |
| 181 | MIMAT0015054 | miR-3177-3p | -0.303508693 | -0.023609981 | 0.008630805 | 0.019267875 | 1.715166172 |
| 182 | MIMAT0000448 | miR-136-5p | 0.315441917 | -0.025348511 | 0.009276808 | 0.019342523 | 1.713486885 |
| 183 | MIMAT0015041 | miR-1260b | 2.472220334 | -0.014942633 | 0.005482143 | 0.019569121 | 1.708428675 |
| 184 | tRF-23-ZKXU53K80E | tRF-23-ZKXU53K80E | -0.828576162 | -0.029284898 | 0.010746692 | 0.019569121 | 1.708428675 |
| 185 | MIMAT0000754 | miR-337-3p | 2.341983166 | -0.020471459 | 0.007585002 | 0.021056258 | 1.676618803 |
| 186 | MIMAT0000259 | miR-182-5p | 3.325635902 | -0.01521379 | 0.005643686 | 0.0211466 | 1.67475945 |
| 187 | MIMAT0003885 | miR-454-3p | -0.338165 | -0.028357254 | 0.010545726 | 0.021462842 | 1.668312766 |
| 188 | tRF-29-6RRNLIK898HR | tRF-29-6RRNLIK898HR | 1.481462137 | 0.051112546 | 0.019058217 | 0.021804772 | 1.661448455 |
| 189 | MIMAT0000081 | miR-25-3p | 7.913109156 | -0.011346873 | 0.004276301 | 0.023608244 | 1.626936319 |
| 190 | MIMAT0000728 | miR-375 | 3.044006093 | 0.01834447 | 0.006920988 | 0.023684826 | 1.625529801 |
| 191 | MIMAT0000092_1 | miR-92a-3p | 9.805738144 | -0.008849606 | 0.003345518 | 0.023936233 | 1.620944203 |
| 192 | MIMAT0004672 | miR-106b-3p | 3.158908435 | -0.019422644 | 0.007376325 | 0.02467734 | 1.607701648 |
| 193 | MIMAT0004496 | miR-23a-5p | 0.703645086 | -0.020216994 | 0.007684527 | 0.024711531 | 1.607100341 |
| 194 | MIMAT0000250 | miR-139-5p | 1.404954039 | -0.016933482 | 0.006569488 | 0.028718897 | 1.541832241 |
| 195 | MIMAT0000765 | miR-335-5p | 5.655308821 | -0.009476394 | 0.003686437 | 0.029154306 | 1.535297297 |
| 196 | MIMAT0001545_1 | miR-450a-5p | 1.65986537 | -0.017357164 | 0.006816635 | 0.031106612 | 1.507147286 |
| 197 | tRF-19-PS5P4PJ4 | tRF-19-PS5P4PJ4 | 1.053841391 | 0.018635992 | 0.007366783 | 0.03244856 | 1.488804568 |
| 198 | trna2-LysCTT_1 | trna2-LysCTT | 1.372528181 | 0.020123153 | 0.00796211 | 0.032503258 | 1.488073111 |
| 199 | MIMAT0004553 | miR-7-1-3p | -0.285046778 | -0.022686431 | 0.008997906 | 0.032902591 | 1.482769905 |
| 200 | trna131-GlyCCC_1 | trna131-GlyCCC | 0.94631712 | 0.018393822 | 0.007398393 | 0.036153182 | 1.441853472 |
| 201 | trna20-GluTTC_1 | trna20-GluTTC | 0.477766149 | 0.02053317 | 0.008284063 | 0.036562497 | 1.436964153 |
| 202 | MIMAT0004702 | miR-339-3p | 2.426674431 | -0.01380686 | 0.005567247 | 0.036562497 | 1.436964153 |
| 203 | MIMAT0000720 | miR-376c-3p | 5.239727371 | -0.013277499 | 0.005373581 | 0.037180492 | 1.429684862 |
| 204 | MIMAT0001413 | miR-20b-5p | -0.646561077 | -0.025344496 | 0.010304926 | 0.038197584 | 1.417964101 |
| 205 | trna64-GlnTTG_1 | trna64-GlnTTG | 0.491140909 | 0.026937155 | 0.01096723 | 0.038362683 | 1.416091031 |
| 206 | MIMAT0004776 | miR-505-5p | -0.463395076 | -0.020921979 | 0.008558725 | 0.039429651 | 1.404177062 |
| 207 | trna5-AspGTC_1 | trna5-AspGTC | 3.819480516 | 0.015161297 | 0.006314099 | 0.044210848 | 1.354471155 |
| 208 | MIMAT0000427_1 | miR-133a-3p | 1.115590091 | -0.022893571 | 0.009556416 | 0.04467115 | 1.349972864 |
| 209 | trna9-ProAGG_2 | trna9-ProAGG | 0.332221095 | -0.015134852 | 0.006352719 | 0.045864571 | 1.338522666 |
| 210 | MIMAT0003340 | miR-542-5p | 0.305235481 | -0.019256073 | 0.008080807 | 0.045864571 | 1.338522666 |
| 211 | MIMAT0004694 | miR-342-5p | -0.751997883 | -0.023860556 | 0.010028825 | 0.046048392 | 1.336785529 |
| 212 | MIMAT0000729_1 | miR-376a-3p | 3.323614493 | -0.015876183 | 0.006693477 | 0.046567037 | 1.331921395 |
| 213 | MIMAT0000722 | miR-370-3p | 1.784151139 | -0.022636225 | 0.009544778 | 0.046567037 | 1.331921395 |
| 214 | MIMAT0004495 | miR-22-5p | 0.989489091 | -0.020712273 | 0.008765271 | 0.047437822 | 1.323875258 |
| 215 | MIMAT0004601 | miR-145-3p | 1.967904169 | -0.012750615 | 0.005416785 | 0.048387098 | 1.315270427 |
| 216 | MIMAT0000730 | miR-377-3p | 0.766213889 | -0.018426809 | 0.007848159 | 0.048945137 | 1.310290452 |
| 217 | MIMAT0004814 | miR-654-3p | 3.355451205 | -0.015813681 | 0.006767893 | 0.050222856 | 1.299098597 |
| 218 | MIMAT0002888 | miR-532-5p | 3.131880041 | -0.017090653 | 0.007328152 | 0.050581404 | 1.296009123 |
| 219 | trna143-LysTTT_1 | trna143-LysTTT | 2.558167583 | 0.011602932 | 0.004999583 | 0.051669496 | 1.286765778 |
| 220 | MIMAT0000086 | miR-29a-3p | 4.056932937 | -0.015536188 | 0.006692134 | 0.051669496 | 1.286765778 |
| 221 | trna10-LysCTT_1 | trna10-LysCTT | 1.126290137 | 0.017053919 | 0.007363787 | 0.052104845 | 1.283121893 |
| 222 | MIMAT0000461 | miR-195-5p | 1.056503624 | -0.013324127 | 0.005760069 | 0.052247123 | 1.281937622 |
| 223 | trna65-AlaAGC_1 | trna65-AlaAGC | 2.9770629 | -0.014426279 | 0.006308874 | 0.055291708 | 1.257339994 |
| 224 | trna10-AspGTC_1 | trna10-AspGTC | 0.700685642 | 0.016633798 | 0.007273655 | 0.055291708 | 1.257339994 |
| 225 | trna12-AspGTC_1 | trna12-AspGTC | 0.700685642 | 0.016633798 | 0.007273655 | 0.055291708 | 1.257339994 |
| 226 | trna91-GlyCCC_1 | trna91-GlyCCC | 3.248228072 | 0.017989138 | 0.007884355 | 0.055780769 | 1.253515505 |
| 227 | MIMAT0004549 | miR-148a-5p | 0.019570179 | -0.017169123 | 0.007542178 | 0.056299044 | 1.24949898 |
| 228 | MIMAT0010133 | miR-2110 | 2.10350593 | -0.011600836 | 0.005187813 | 0.06224002 | 1.205930275 |
| 229 | MIMAT0003241 | miR-576-5p | -1.409265054 | -0.025798169 | 0.011614157 | 0.064114467 | 1.193043961 |
| 230 | MIMAT0000421 | miR-122-5p | 5.916803447 | 0.014506691 | 0.006529323 | 0.064114467 | 1.193043961 |
| 231 | tRF-35-LSM1M3WE8SSP6D | tRF-35-LSM1M3WE8SSP6D | 0.658746268 | 0.031199091 | 0.01410523 | 0.065393643 | 1.184464471 |
| 232 | trna15-ValAAC_1 | trna15-ValAAC | 1.069433488 | 0.014012144 | 0.006340674 | 0.065445959 | 1.184117167 |
| 233 | MIMAT0000069_1 | miR-16-5p | 8.747724365 | -0.009193672 | 0.004173814 | 0.066372448 | 1.178012162 |
| 234 | MIMAT0001629_1 | miR-329-3p | 1.801066083 | -0.018826892 | 0.008577719 | 0.067424104 | 1.171184816 |
| 235 | MIMAT0000510 | miR-320a | 6.457362325 | -0.00969183 | 0.004430067 | 0.068364351 | 1.165170303 |
| 236 | ENST00000362698 | RNU5A-1 | 7.136113273 | -0.03115847 | 0.014322821 | 0.07023014 | 1.153476465 |
| 237 | ENST00000365651 | RNA5S7 | 1.255520704 | 0.032344471 | 0.014960735 | 0.072354509 | 1.140534398 |
| 238 | tRF-18-YRRHQFD2 | tRF-18-YRRHQFD2 | 1.027228563 | -0.015740819 | 0.007304834 | 0.073349648 | 1.134601966 |
| 239 | ENST00000363040 | RNA5S10 | 1.250808397 | 0.032189843 | 0.015002566 | 0.074194606 | 1.12962767 |
| 240 | ENST00000365656 | RNA5S13 | 1.250808397 | 0.032189843 | 0.015002566 | 0.074194606 | 1.12962767 |
| 241 | MIMAT0000073 | miR-19a-3p | 3.537805321 | -0.010029802 | 0.00467528 | 0.074194606 | 1.12962767 |
| 242 | MIMAT0000432 | miR-141-3p | 0.865476494 | -0.014178283 | 0.006630763 | 0.075196864 | 1.123800268 |
| 243 | ENST00000365387 | RNA5S16 | 1.248322794 | 0.032201791 | 0.01517617 | 0.078006012 | 1.107871925 |
| 244 | ENST00000362526 | RNA5S5 | 1.263656457 | 0.031637228 | 0.014931141 | 0.078211463 | 1.106729592 |
| 245 | ENST00000362464 | RNA5S11 | 1.243434139 | 0.032085518 | 0.01516433 | 0.078211463 | 1.106729592 |
| 246 | ENST00000365055 | RNA5S15 | 1.243434139 | 0.032085518 | 0.01516433 | 0.078211463 | 1.106729592 |
| 247 | ENST00000363473 | RNA5S8 | 1.24545861 | 0.032068961 | 0.015226297 | 0.079212703 | 1.101205168 |
| 248 | ENST00000364485 | RNA5S14 | 1.24358688 | 0.032046536 | 0.015218193 | 0.079212703 | 1.101205168 |
| 249 | ENST00000363500 | RNA5S17 | 1.24358688 | 0.032046536 | 0.015218193 | 0.079212703 | 1.101205168 |
| 250 | ENST00000362482 | RNA5S1 | 1.238682123 | 0.03192975 | 0.01520634 | 0.080078811 | 1.096482384 |
| 251 | ENST00000364718 | RNA5S2 | 1.254118047 | 0.031367866 | 0.014961435 | 0.080387122 | 1.094813519 |
| 252 | trna28-ProTGG_1 | trna28-ProTGG | 1.001139247 | -0.011086506 | 0.00530799 | 0.081643775 | 1.088076923 |
| 253 | ENST00000362467 | RNA5S3 | 1.226596938 | 0.031462964 | 0.015161017 | 0.083763989 | 1.07694265 |
| 254 | tRF-35-87R8WP9N1EWJQ7 | tRF-35-87R8WP9N1EWJQ7 | 1.726421252 | 0.017047622 | 0.008215995 | 0.083763989 | 1.07694265 |
| 255 | ENST00000363511 | RNA5S4 | 1.248780541 | 0.031247768 | 0.015185205 | 0.086728326 | 1.061839038 |
| 256 | ENST00000363754 | RNA5S6 | 1.246913118 | 0.031225407 | 0.01517708 | 0.086728326 | 1.061839038 |
| 257 | trna35-SerAGA_1 | trna35-SerAGA | 0.384575962 | -0.016324084 | 0.007979818 | 0.088878144 | 1.051205023 |
| 258 | trna8-ProTGG_1 | trna8-ProTGG | 1.011918334 | -0.010777694 | 0.005284397 | 0.089853963 | 1.046462762 |
| 259 | trna31-ProTGG_1 | trna31-ProTGG | 0.272897572 | 0.021739195 | 0.010700821 | 0.090891819 | 1.041475204 |
| 260 | ENST00000362400 | RNA5S12 | 1.234896974 | 0.030760978 | 0.015131799 | 0.090891819 | 1.041475204 |
| 261 | MIMAT0004748 | miR-423-5p | 8.234743251 | -0.006228651 | 0.003069848 | 0.091103374 | 1.04046554 |
| 262 | trna99-ValCAC_1 | trna99-ValCAC | 1.544445333 | 0.013429052 | 0.00672447 | 0.09793709 | 1.009052803 |
| 263 | trna18-GlyGCC_1 | trna18-GlyGCC | 4.8198881 | -0.0150317 | 0.007542393 | 0.098512421 | 1.006509008 |
| 264 | MIMAT0004606 | miR-136-3p | 4.291043719 | -0.013022194 | 0.006541316 | 0.098651682 | 1.005895505 |
| 265 | trna25-GlyGCC_1 | trna25-GlyGCC | 4.882471197 | -0.014562268 | 0.00733979 | 0.099857406 | 1.00061972 |
| 266 | ENST00000364662 | RNA5SP194 | -0.128975951 | 0.049769684 | 0.025261577 | 0.102775827 | 0.988109021 |
| 267 | MIMAT0022705 | miR-539-3p | -0.137108421 | -0.020863785 | 0.010648942 | 0.105047656 | 0.978613634 |
| 268 | MIMAT0003218 | miR-92b-3p | 2.585041071 | -0.008183454 | 0.004214389 | 0.108996666 | 0.962586785 |
| 269 | trna19-GlyGCC_1 | trna19-GlyGCC_1 | 4.864614479 | -0.01423 | 0.007348123 | 0.109611323 | 0.960144579 |
| 270 | MIMAT0000681 | miR-29c-3p | 2.953572836 | -0.012307991 | 0.006356914 | 0.109611323 | 0.960144579 |
| 271 | trna19-GlyGCC_2 | trna19-GlyGCC | 4.856801911 | -0.014059941 | 0.007271961 | 0.10983422 | 0.959262332 |
| 272 | trna5-GlyGCC_1 | trna5-GlyGCC | 4.877752888 | -0.013901712 | 0.007195569 | 0.10983422 | 0.959262332 |
| 273 | MIMAT0000077 | miR-22-3p | 8.35182996 | -0.007597186 | 0.003935334 | 0.10983422 | 0.959262332 |
| 274 | trna128-GlyGCC_1 | trna128-GlyGCC | 4.851237233 | -0.014164339 | 0.00734336 | 0.109849521 | 0.959201832 |
| 275 | trna2-SerCGA_1 | trna2-SerCGA | 1.307694596 | -0.010770452 | 0.00559694 | 0.110597302 | 0.956255466 |
| 276 | MIMAT0000074_1 | miR-19b-3p | 5.004750074 | -0.00847136 | 0.004409475 | 0.111005841 | 0.954654169 |
| 277 | trna68-GlyGCC_1 | trna68-GlyGCC | 4.880093503 | -0.013862518 | 0.00723702 | 0.111010829 | 0.954634655 |
| 278 | trna24-GlyGCC_1 | trna24-GlyGCC | 4.867912428 | -0.01393844 | 0.007278944 | 0.111010829 | 0.954634655 |
| 279 | trna12-ProTGG_1 | trna12-ProTGG | -0.053081891 | -0.016176846 | 0.008429079 | 0.111010829 | 0.954634655 |
| 280 | tRF-18-H7PU4HD2 | tRF-18-H7PU4HD2 | 0.05743888 | -0.017907975 | 0.00934293 | 0.111010829 | 0.954634655 |
| 281 | MIMAT0000222 | miR-192-5p | 4.782913368 | -0.008269933 | 0.004398974 | 0.11979683 | 0.921554673 |
| 282 | ENST00000629629 | snoU18 | 0.665573207 | -0.044233894 | 0.023742815 | 0.124026433 | 0.906485746 |
| 283 | MIMAT0001638 | miR-409-5p | -0.459882995 | -0.020981897 | 0.011372958 | 0.128273559 | 0.891862857 |
| 284 | MIMAT0000261 | miR-183-5p | 1.306408976 | -0.015103395 | 0.008181197 | 0.128273559 | 0.891862857 |
| 285 | MIMAT0003329 | miR-411-5p | -0.72012673 | -0.01830465 | 0.009997776 | 0.131884179 | 0.8798073 |
| 286 | MIMAT0000426 | miR-132-3p | 0.697203476 | -0.013192553 | 0.007228275 | 0.133109954 | 0.875789468 |
| 287 | MIMAT0002177_1 | miR-486-5p | 10.41328438 | 0.006999766 | 0.003852087 | 0.135017155 | 0.869611047 |
| 288 | trna38-IleAAT_1 | trna38-IleAAT | -0.549171946 | 0.020595355 | 0.01134454 | 0.135053341 | 0.869494667 |
| 289 | ENST00000363286 | RNU5B-1 | 1.267728288 | -0.036182632 | 0.020061797 | 0.138160136 | 0.859617248 |
| 290 | tRF-22-PS5P4PW3H | tRF-22-PS5P4PW3H | 1.089193333 | -0.011552951 | 0.006432816 | 0.140007868 | 0.853847556 |
| 291 | trna9-LysCTT_1 | trna9-LysCTT | 1.509576843 | 0.010363213 | 0.00578467 | 0.140892387 | 0.851112474 |
| 292 | MIMAT0000416_1 | miR-1-3p | 0.83536753 | -0.017237024 | 0.009649487 | 0.142010347 | 0.847680013 |
| 293 | trna12-TrpCCA_1 | trna12-TrpCCA | 1.221058177 | 0.012950531 | 0.007273411 | 0.143324039 | 0.843680963 |
| 294 | MIMAT0000244_1 | miR-30c-5p | 2.592315113 | -0.009444083 | 0.005366323 | 0.14938704 | 0.825687079 |
| 295 | trna27-GlyCCC_1 | trna27-GlyCCC | 1.880334871 | 0.008716191 | 0.004957198 | 0.149393181 | 0.825669224 |
| 296 | MIMAT0000263 | miR-199b-5p | 0.942394508 | -0.02143162 | 0.012274064 | 0.152856012 | 0.815717476 |
| 297 | trna56-ThrTGT_1 | trna56-ThrTGT | -0.216226299 | 0.025686547 | 0.014767513 | 0.154547173 | 0.810938936 |
| 298 | MIMAT0004570 | miR-223-5p | 0.724333527 | -0.015513789 | 0.008959771 | 0.156656669 | 0.805051111 |
| 299 | trna27-LeuTAG_1 | trna27-LeuTAG | -0.600677097 | 0.018644682 | 0.010822715 | 0.159077975 | 0.798389947 |
| 300 | trna52-ArgTCT_1 | trna52-ArgTCT | 0.851048975 | 0.014602522 | 0.008503154 | 0.159327734 | 0.797708622 |
| 301 | trna4-GlyCCC_1 | trna4-GlyCCC | 4.971406822 | -0.012060035 | 0.007018649 | 0.159327734 | 0.797708622 |
| 302 | trna123-SerGCT_1 | trna123-SerGCT | 0.368210735 | 0.019225829 | 0.011186637 | 0.159327734 | 0.797708622 |
| 303 | trna5-GluTTC_1 | trna5-GluTTC_1 | 0.117405948 | 0.014658414 | 0.008552819 | 0.159965459 | 0.795973784 |
| 304 | trna133-GlyCCC_1 | trna133-GlyCCC | 4.966537224 | -0.011877845 | 0.006995736 | 0.164928198 | 0.782705087 |
| 305 | MIMAT0003884 | miR-454-5p | -1.061188179 | -0.018120508 | 0.010725855 | 0.1667892 | 0.777832074 |
| 306 | MIMAT0022720 | miR-1304-3p | -0.060973697 | -0.01398831 | 0.008274085 | 0.1667892 | 0.777832074 |
| 307 | MIMAT0019856 | miR-4732-3p | 0.160724539 | 0.014757679 | 0.008787946 | 0.169809142 | 0.770038933 |
| 308 | ENST00000362512 | RNU12 | 2.006569692 | -0.008178095 | 0.004946397 | 0.178656211 | 0.747981881 |
| 309 | MIMAT0030413 | miR-4433b-5p | 5.235179875 | -0.010519506 | 0.00637768 | 0.179527216 | 0.745869703 |
| 310 | MIMAT0000735 | miR-380-3p | 0.966439743 | -0.010647616 | 0.006465716 | 0.179928224 | 0.744900707 |
| 311 | trna10-SerGCT_1 | trna10-SerGCT | 0.375074871 | 0.018455755 | 0.011272863 | 0.182930841 | 0.737713069 |
| 312 | trna144-AspGTC_1 | trna144-AspGTC | -0.702951404 | 0.016877168 | 0.010543919 | 0.195827413 | 0.708126513 |
| 313 | tRF-18-PJ583004 | tRF-18-PJ583004 | -0.53056419 | -0.016055406 | 0.010028156 | 0.195827413 | 0.708126513 |
| 314 | trna4-LysCTT_1 | trna4-LysCTT | -0.965815145 | 0.024221712 | 0.015167713 | 0.196681714 | 0.706236016 |
| 315 | MIMAT0003330 | miR-654-5p | -0.193792781 | -0.01409926 | 0.008865018 | 0.198642087 | 0.70192873 |
| 316 | MIMAT0004586 | miR-15b-3p | 2.232396183 | -0.009505997 | 0.006008407 | 0.201357405 | 0.696032393 |
| 317 | trna17-GluTTC_1 | trna17-GluTTC | -0.977650231 | -0.017504685 | 0.011081709 | 0.201738032 | 0.69521222 |
| 318 | MIMAT0000087 | miR-30a-5p | 4.407541766 | -0.00565054 | 0.003608494 | 0.205401141 | 0.687397148 |
| 319 | MIMAT0000270 | miR-181a-3p | -0.668239461 | -0.014734425 | 0.009409231 | 0.205401141 | 0.687397148 |
| 320 | MIMAT0022697 | miR-382-3p | 0.961782242 | -0.014327028 | 0.009136387 | 0.205401141 | 0.687397148 |
| 321 | MIMAT0000737 | miR-382-5p | 1.400022045 | -0.011142131 | 0.007170232 | 0.209691254 | 0.678419682 |
| 322 | trna106-PheGAA_1 | trna106-PheGAA | 2.140658915 | 0.010851888 | 0.007016613 | 0.212104175 | 0.673450782 |
| 323 | MIMAT0000100_1 | miR-29b-3p | 3.244681093 | -0.008952843 | 0.005805865 | 0.213363926 | 0.670879006 |
| 324 | trna23-LysTTT_1 | trna23-LysTTT | 1.010712439 | 0.01043332 | 0.006823703 | 0.218242462 | 0.661060748 |
| 325 | trna34-GlyCCC_1 | trna34-GlyCCC | 5.398551806 | 0.007398688 | 0.004861837 | 0.22066081 | 0.656274793 |
| 326 | trna11-LysTTT_1 | trna11-LysTTT | 1.02325351 | 0.010327289 | 0.006807358 | 0.222019584 | 0.653608715 |
| 327 | MIMAT0017994 | miR-3615 | 2.229276179 | -0.008228319 | 0.005450789 | 0.224607105 | 0.64857651 |
| 328 | ENST00000363214 | SNORD68 | 0.95254887 | -0.03193686 | 0.021230858 | 0.226241477 | 0.645427772 |
| 329 | tRF-32-P4R8YP9LON4V3 | tRF-32-P4R8YP9LON4V3 | 2.630249766 | 0.018020878 | 0.012125866 | 0.233598215 | 0.631530481 |
| 330 | trna105-AlaAGC_1 | trna105-AlaAGC | -1.38147078 | 0.020705703 | 0.013958678 | 0.234147165 | 0.630511095 |
| 331 | MIMAT0004911 | miR-874-3p | 0.069390615 | -0.011522659 | 0.007787077 | 0.235080174 | 0.628783996 |
| 332 | trna45-AspGTC_1 | trna45-AspGTC | 0.252903934 | 0.012234466 | 0.008343189 | 0.240427058 | 0.619016657 |
| 333 | trna16-GlnTTG_2 | trna16-GlnTTG | -0.681226437 | 0.016575242 | 0.011321666 | 0.240793524 | 0.618355197 |
| 334 | trna173-GlnTTG_1 | trna173-GlnTTG | -1.357192153 | 0.020137285 | 0.013789586 | 0.241051764 | 0.617889686 |
| 335 | trna174-GlnTTG_1 | trna174-GlnTTG | -1.357192153 | 0.020137285 | 0.013789586 | 0.241051764 | 0.617889686 |
| 336 | trna148-SerTGA_1 | trna148-SerTGA | -0.455618039 | 0.015519232 | 0.010644206 | 0.241401793 | 0.617259508 |
| 337 | trna74-LeuCAA_1 | trna74-LeuCAA | -1.101326529 | -0.015069242 | 0.010440268 | 0.247242242 | 0.606877327 |
| 338 | ENST00000390930 | SNORD17 | 1.553007303 | -0.030700527 | 0.021286367 | 0.247242242 | 0.606877327 |
| 339 | trna38-AspGTC_1 | trna38-AspGTC | 0.244479416 | 0.011979658 | 0.008370419 | 0.250971906 | 0.600374892 |
| 340 | ENST00000363981 | SNORD27 | 3.062769233 | -0.028007561 | 0.019556274 | 0.250971906 | 0.600374892 |
| 341 | tRF-18-0P583004 | tRF-18-0P583004 | 0.700148653 | -0.009931819 | 0.006949169 | 0.251169035 | 0.600033904 |
| 342 | MIMAT0003332 | miR-656-3p | 0.804710749 | -0.013597558 | 0.00953303 | 0.251776743 | 0.598984388 |
| 343 | ENST00000383884 | SNORD24 | 0.054060198 | -0.022467259 | 0.015769718 | 0.25182261 | 0.59890528 |
| 344 | trna152-ValCAC_1 | trna152-ValCAC | -1.091206264 | 0.017685655 | 0.012475814 | 0.254456458 | 0.594386522 |
| 345 | tRF-32-87R8WP9N1EWJM | tRF-32-87R8WP9N1EWJM | 4.77521434 | -0.013479745 | 0.009577248 | 0.258551741 | 0.587452533 |
| 346 | MIMAT0004482 | let-7b-3p | 0.645955662 | -0.009397905 | 0.006695361 | 0.259647123 | 0.585616485 |
| 347 | trna43-SerGCT_1 | trna43-SerGCT | 0.511080183 | 0.014136725 | 0.010144868 | 0.263545252 | 0.579144803 |
| 348 | trna40-ValTAC_1 | trna40-ValTAC | 1.03161728 | 0.013879652 | 0.009967517 | 0.263545252 | 0.579144803 |
| 349 | MIMAT0000447 | miR-134-5p | 1.149178493 | -0.012528266 | 0.009166646 | 0.275526661 | 0.559836371 |
| 350 | MIMAT0000090 | miR-32-5p | 1.352322131 | -0.009885221 | 0.007253262 | 0.275891592 | 0.559261535 |
| 351 | MIMAT0004800_1 | miR-550a-5p | -0.128029693 | -0.012294847 | 0.009020151 | 0.275891592 | 0.559261535 |
| 352 | MIMAT0000104 | miR-107 | 2.874710503 | -0.007013899 | 0.005171791 | 0.278473802 | 0.555215655 |
| 353 | trna31-SerGCT_1 | trna31-SerGCT | 0.499536087 | 0.013735224 | 0.010179598 | 0.28118032 | 0.551015079 |
| 354 | tRF-32-Q1Q89P9L8422Q | tRF-32-Q1Q89P9L8422Q | -0.211075398 | 0.019773081 | 0.014768816 | 0.28573092 | 0.544042761 |
| 355 | trna9-ValCAC_1 | trna9-ValCAC | 0.996439514 | -0.008249517 | 0.006186354 | 0.286065925 | 0.543533871 |
| 356 | MIMAT0000272 | miR-215-5p | 0.619990035 | -0.009863154 | 0.007393432 | 0.286065925 | 0.543533871 |
| 357 | tRF-28-PS5P4PW3FJDD | tRF-28-PS5P4PW3FJDD | -0.562025063 | -0.013055453 | 0.009783804 | 0.286065925 | 0.543533871 |
| 358 | MIMAT0000755 | miR-323a-3p | 2.62309999 | -0.009615348 | 0.007236245 | 0.286897698 | 0.542272936 |
| 359 | MIMAT0000098 | miR-100-5p | 4.232347403 | -0.008596042 | 0.006467612 | 0.286897698 | 0.542272936 |
| 360 | trna81-LeuTAA_1 | trna81-LeuTAA | -0.159464076 | 0.014294901 | 0.010775191 | 0.28719392 | 0.541824758 |
| 361 | MIMAT0000732 | miR-378a-3p | 2.181985023 | -0.008748229 | 0.006636535 | 0.290764629 | 0.536458426 |
| 362 | trna166-AlaAGC_1 | trna166-AlaAGC | -0.228867751 | 0.012199094 | 0.009333712 | 0.295802493 | 0.528998171 |
| 363 | trna9-ArgTCT_1 | trna9-ArgTCT | -0.182558086 | -0.011735627 | 0.009036249 | 0.299044417 | 0.524264301 |
| 364 | trna2-GlyCCC_2 | trna2-GlyCCC | -1.304289002 | 0.017603241 | 0.013564602 | 0.299044417 | 0.524264301 |
| 365 | trna127-CysGCA_1 | trna127-CysGCA | 0.494235938 | 0.009881321 | 0.007650421 | 0.300646545 | 0.521943782 |
| 366 | trna130-GlnTTG_1 | trna130-GlnTTG | -1.336864186 | 0.017813715 | 0.013777486 | 0.300646545 | 0.521943782 |
| 367 | trna16-HisGTG_1 | trna16-HisGTG | -0.262449456 | 0.013198156 | 0.010262512 | 0.301130997 | 0.521244539 |
| 368 | trna3-ProTGG_2 | trna3-ProTGG | 0.739198715 | -0.00748677 | 0.005819325 | 0.301130997 | 0.521244539 |
| 369 | trna2-ValAAC_1 | trna2-ValAAC | 0.610105998 | -0.009154853 | 0.007114516 | 0.301130997 | 0.521244539 |
| 370 | MIMAT0003244 | miR-579-3p | -0.504212734 | -0.012092692 | 0.009419391 | 0.301505593 | 0.520704627 |
| 371 | tRF-18-1SS2PMX | tRF-18-1SS2PMX | -0.170900293 | -0.013758512 | 0.010732584 | 0.301681839 | 0.520450833 |
| 372 | trna14-ProTGG_1 | trna14-ProTGG | 0.726959291 | -0.007338732 | 0.005825936 | 0.312802173 | 0.504730239 |
| 373 | MIMAT0001627 | miR-433-3p | 1.500395518 | -0.009521039 | 0.007590313 | 0.31484406 | 0.501904496 |
| 374 | trna11-LysCTT_1 | trna11-LysCTT | 1.055601934 | 0.007563595 | 0.006040857 | 0.315251853 | 0.501342352 |
| 375 | tRF-18-S5R83004 | tRF-18-S5R83004 | 0.673851503 | -0.008704435 | 0.007069116 | 0.325842981 | 0.486991629 |
| 376 | MIMAT0004518 | miR-16-2-3p | 4.23855121 | -0.005728067 | 0.004674317 | 0.328272829 | 0.483765062 |
| 377 | MIMAT0000707 | miR-363-3p | 5.776784022 | -0.004562736 | 0.003743391 | 0.331084243 | 0.480061488 |
| 378 | trna48-AspGTC_1 | trna48-AspGTC | 0.110046075 | 0.010602743 | 0.008835676 | 0.340951632 | 0.467307227 |
| 379 | MIMAT0005911 | miR-1260a | 0.481464079 | -0.009130451 | 0.007630229 | 0.341993693 | 0.465981904 |
| 380 | trna13-LysCTT_3 | trna13-LysCTT | 1.16696618 | 0.006869184 | 0.005758835 | 0.34238584 | 0.465484205 |
| 381 | MIMAT0001621 | miR-369-5p | -0.347015902 | -0.011876323 | 0.009944904 | 0.34238584 | 0.465484205 |
| 382 | ENST00000613359 | RNA5-8S5_2 | 3.915704302 | -0.005689386 | 0.004780166 | 0.342985587 | 0.46472413 |
| 383 | trna98-ValCAC_1 | trna98-ValCAC | -1.303732885 | 0.014810937 | 0.012494168 | 0.344843395 | 0.462378087 |
| 384 | ENST00000612463 | RNA5-8S5 | 3.911599565 | -0.005623632 | 0.004779002 | 0.348869485 | 0.457337016 |
| 385 | ENST00000619471 | RNA5-8S5_3 | 3.913349783 | -0.005626227 | 0.004789406 | 0.348869485 | 0.457337016 |
| 386 | MIMAT0000095 | miR-96-5p | 3.189702871 | 0.007398948 | 0.006310576 | 0.348869485 | 0.457337016 |
| 387 | tRF-34-87R8WP9N1EWJI5 | tRF-34-87R8WP9N1EWJI5 | 0.47666621 | 0.010586413 | 0.009030792 | 0.348869485 | 0.457337016 |
| 388 | trna6-ValTAC_1 | trna6-ValTAC | 0.355995929 | 0.015524397 | 0.013302042 | 0.350985219 | 0.454711172 |
| 389 | trna85-ValCAC_1 | trna85-ValCAC | -1.220453501 | 0.01380334 | 0.012003958 | 0.359722689 | 0.444032169 |
| 390 | ENST00000580533 | SNORD3C | -0.201541523 | -0.025619631 | 0.02229569 | 0.359722689 | 0.444032169 |
| 391 | trna7-LysCTT_1 | trna7-LysCTT | 1.050189284 | 0.006856852 | 0.006009963 | 0.363649989 | 0.439316422 |
| 392 | trna6-ProTGG_1 | trna6-ProTGG | 0.729199409 | -0.006625391 | 0.005823166 | 0.363681305 | 0.439279023 |
| 393 | ENST00000584923 | SNORD3A | -0.207170907 | -0.025335378 | 0.022277303 | 0.363681305 | 0.439279023 |
| 394 | ENST00000610460 | RNA5-8S5_1 | 3.917032667 | -0.005421848 | 0.004771943 | 0.363681305 | 0.439279023 |
| 395 | trna17-ValTAC_1 | trna17-ValTAC | 0.527964933 | 0.008904731 | 0.007875802 | 0.366062548 | 0.436444701 |
| 396 | trna18-ValCAC_1 | trna18-ValCAC | -1.28470992 | 0.013754628 | 0.012292167 | 0.371195052 | 0.430397822 |
| 397 | trna2-ValCAC_1 | trna2-ValCAC | -1.28470992 | 0.013754628 | 0.012292167 | 0.371195052 | 0.430397822 |
| 398 | trna10-ValCAC_1 | trna10-ValCAC | 0.938229155 | -0.00704473 | 0.006315225 | 0.37234318 | 0.429056597 |
| 399 | ENST00000362761 | SNORD33 | 0.509031668 | -0.011849435 | 0.01066016 | 0.37379126 | 0.427370857 |
| 400 | ENST00000408876 | SNORD23 | 0.178815313 | -0.025758582 | 0.023244775 | 0.374918848 | 0.426062726 |
| 401 | ENST00000410396 | RNU2-2P | 2.808349012 | -0.018704809 | 0.017105292 | 0.382880675 | 0.416936553 |
| 402 | MIMAT0000068 | miR-15a-5p | 4.239150896 | -0.005705442 | 0.005256693 | 0.38501259 | 0.414525069 |
| 403 | MIMAT0000253 | miR-10a-5p | 5.916205994 | -0.003325801 | 0.003060949 | 0.38501259 | 0.414525069 |
| 404 | tRF-31-PS5P4PW3FJHPB | tRF-31-PS5P4PW3FJHPB | 3.336444934 | -0.008814905 | 0.00810101 | 0.38501259 | 0.414525069 |
| 405 | MIMAT0004774 | miR-501-3p | 3.245694835 | 0.00413765 | 0.003862669 | 0.39280796 | 0.40581972 |
| 406 | ENST00000410361 | RNU2-36P | 2.594835439 | -0.020495392 | 0.019172668 | 0.393205693 | 0.405380203 |
| 407 | ENST00000384756 | SNORD22 | 0.171093291 | -0.022315888 | 0.021048382 | 0.397703369 | 0.40044073 |
| 408 | trna54-LysTTT_1 | trna54-LysTTT | 3.488624966 | 0.003874867 | 0.003685436 | 0.402259833 | 0.395493331 |
| 409 | tRF-32-PS5P4PW3FJHP1 | tRF-32-PS5P4PW3FJHP1 | 2.182929561 | -0.013941666 | 0.013339609 | 0.405228461 | 0.39230006 |
| 410 | trna59-GluCTC_1 | trna59-GluCTC | 2.99553351 | -0.005580885 | 0.005391693 | 0.409524692 | 0.387719908 |
| 411 | trna62-LysTTT_1 | trna62-LysTTT | 3.503769445 | 0.00375199 | 0.003627041 | 0.409524692 | 0.387719908 |
| 412 | ENST00000619178 | SNORD3D | -0.308249712 | -0.025330605 | 0.024505642 | 0.409524692 | 0.387719908 |
| 413 | trna3-CysGCA_2 | trna3-CysGCA | 0.462698018 | 0.007748833 | 0.007517596 | 0.410377833 | 0.386816106 |
| 414 | trna5-LysTTT_1 | trna5-LysTTT | 3.469557501 | 0.003822323 | 0.003728626 | 0.412969363 | 0.384082166 |
| 415 | ENST00000571722 | SNORD3B-2_1 | -0.26538726 | -0.024298473 | 0.023766935 | 0.413736602 | 0.383276057 |
| 416 | trna6-AlaAGC_1 | trna6-AlaAGC | -0.081552051 | 0.00888733 | 0.00878451 | 0.419568238 | 0.377197396 |
| 417 | trna27-CysGCA_1 | trna27-CysGCA | -1.072411134 | -0.010385518 | 0.010323824 | 0.422252262 | 0.374428015 |
| 418 | trna22-ProAGG_1 | trna22-ProAGG | -0.027839491 | -0.007239722 | 0.007292055 | 0.429774609 | 0.366759246 |
| 419 | trna90-ValCAC_1 | trna90-ValCAC | -1.223600292 | 0.01187747 | 0.012011477 | 0.431346662 | 0.365173558 |
| 420 | trna26-CysGCA_1 | trna26-CysGCA | -1.141776591 | -0.010235149 | 0.01052501 | 0.440048039 | 0.35649991 |
| 421 | trna3-CysGCA_1 | trna3-CysGCA_1 | -1.141776591 | -0.010235149 | 0.01052501 | 0.440048039 | 0.35649991 |
| 422 | trna137-SerCGA_1 | trna137-SerCGA | -1.093064101 | 0.012819952 | 0.013345962 | 0.445828946 | 0.350831738 |
| 423 | tRF-29-ORRNLNK8QJI1 | tRF-29-ORRNLNK8QJI1 | -0.439527672 | 0.011027661 | 0.011478919 | 0.445828946 | 0.350831738 |
| 424 | trna76-LysTTT_1 | trna76-LysTTT | 3.486589394 | 0.003511083 | 0.003684965 | 0.449948166 | 0.346837514 |
| 425 | tRF-33-87R8WP9N1EWJDW | tRF-33-87R8WP9N1EWJDW | 3.018122423 | 0.007448743 | 0.007830495 | 0.449948166 | 0.346837514 |
| 426 | trna2-ArgCCT_1 | trna2-ArgCCT | -1.48827986 | 0.013131983 | 0.013851961 | 0.45104653 | 0.345778654 |
| 427 | MIMAT0005825 | miR-1180-3p | 1.400368325 | 0.006584427 | 0.007005917 | 0.455476278 | 0.341534237 |
| 428 | trna14-LysTTT_1 | trna14-LysTTT | 3.48676058 | 0.003421993 | 0.003680973 | 0.461287736 | 0.336028091 |
| 429 | MIMAT0004695 | miR-337-5p | 0.693290376 | -0.00767094 | 0.00826878 | 0.461528312 | 0.335801653 |
| 430 | tRF-19-FMD0SR1Z | tRF-19-FMD0SR1Z | 1.330493182 | -0.006568877 | 0.007143321 | 0.465959964 | 0.331651397 |
| 431 | ENST00000616345 | RNU2-1_1 | -0.707729969 | -0.011703739 | 0.012982896 | 0.470728728 | 0.327229297 |
| 432 | ENST00000613119 | RNU2-1_1_X | -0.707729969 | -0.011703739 | 0.012982896 | 0.470728728 | 0.327229297 |
| 433 | ENST00000613778 | RNU2-1_5 | -0.707729969 | -0.011703739 | 0.012982896 | 0.470728728 | 0.327229297 |
| 434 | ENST00000618602 | RNU2-1_10 | -0.707729969 | -0.011703739 | 0.012982896 | 0.470728728 | 0.327229297 |
| 435 | MIMAT0004558 | miR-181a-2-3p | 0.432665554 | -0.006750808 | 0.007427317 | 0.470728728 | 0.327229297 |
| 436 | MIMAT0001631 | miR-451a | 10.01209231 | -0.00478832 | 0.005308294 | 0.470728728 | 0.327229297 |
| 437 | MIMAT0004799 | miR-589-5p | 2.916662569 | -0.004072597 | 0.004507193 | 0.470728728 | 0.327229297 |
| 438 | trna4-ValTAC_1 | trna4-ValTAC | 0.456626237 | 0.007450818 | 0.008314415 | 0.473292052 | 0.324870789 |
| 439 | trna4-ArgTCG_1 | trna4-ArgTCG_1 | 0.02321558 | 0.00911487 | 0.010345245 | 0.481508032 | 0.317396464 |
| 440 | trna15-CysGCA_1 | trna15-CysGCA | -1.08356768 | -0.009076593 | 0.010302817 | 0.481508032 | 0.317396464 |
| 441 | ENST00000617785 | RNU2-1_8 | -0.719887438 | -0.011375182 | 0.01293549 | 0.481519329 | 0.317386275 |
| 442 | MIMAT0000772 | miR-345-5p | 1.692753804 | -0.005052007 | 0.005795908 | 0.48575433 | 0.313583319 |
| 443 | trna71-LysTTT_1 | trna71-LysTTT | -0.439489055 | 0.008676605 | 0.01006256 | 0.491158018 | 0.308778761 |
| 444 | trna13-AlaTGC_1 | trna13-AlaTGC | -1.208067048 | -0.009579388 | 0.011171135 | 0.492341425 | 0.307733622 |
| 445 | MIMAT0000682 | miR-200a-3p | -0.634426925 | -0.008763303 | 0.01022104 | 0.492341425 | 0.307733622 |
| 446 | trna8-AlaTGC_1 | trna8-AlaTGC_1 | -1.174370919 | -0.009354797 | 0.010974598 | 0.494695491 | 0.305662048 |
| 447 | trna8-SerGCT_1 | trna8-SerGCT | 0.968329756 | 0.007146314 | 0.008414756 | 0.495777628 | 0.304713074 |
| 448 | ENST00000616535 | RNU2-1_2 | -0.711382187 | -0.010872801 | 0.0129885 | 0.503163548 | 0.29829083 |
| 449 | trna32-LysCTT_1 | trna32-LysCTT | 0.598707271 | 0.005551047 | 0.006675067 | 0.505907728 | 0.295928687 |
| 450 | MIMAT0004610 | miR-150-3p | 0.247113865 | -0.010424904 | 0.012591065 | 0.507351117 | 0.29469138 |
| 451 | ENST00000618664 | RNU2-1 | -0.723570603 | -0.010535145 | 0.012939938 | 0.509213807 | 0.293099829 |
| 452 | ENST00000619465 | RNU2-1_3 | -0.723570603 | -0.010535145 | 0.012939938 | 0.509213807 | 0.293099829 |
| 453 | ENST00000611544 | RNU2-1_6 | -0.723570603 | -0.010535145 | 0.012939938 | 0.509213807 | 0.293099829 |
| 454 | ENST00000615427 | RNU2-1_7 | -0.723570603 | -0.010535145 | 0.012939938 | 0.509213807 | 0.293099829 |
| 455 | ENST00000620268 | RNU2-1_9 | -0.723570603 | -0.010535145 | 0.012939938 | 0.509213807 | 0.293099829 |
| 456 | ENST00000362883 | SNORD104 | 2.109593663 | -0.016235179 | 0.019936479 | 0.509213807 | 0.293099829 |
| 457 | MIMAT0000267 | miR-210-3p | 1.966645409 | 0.005752029 | 0.006984426 | 0.509213807 | 0.293099829 |
| 458 | MIMAT0020925 | miR-550a-3-5p | -0.550854271 | 0.008505811 | 0.010497503 | 0.510828894 | 0.291724546 |
| 459 | trna2-LysTTT_2 | trna2-LysTTT | 3.746635343 | -0.002503928 | 0.003135615 | 0.51781003 | 0.285829541 |
| 460 | MIMAT0002871 | miR-500a-3p | 2.769370874 | -0.003401784 | 0.004267248 | 0.51781003 | 0.285829541 |
| 461 | MIMAT0015050 | miR-323b-3p | 3.908566869 | -0.005225035 | 0.006586959 | 0.518349508 | 0.285377309 |
| 462 | tRF-31-PSQP4PW3FJI0B | tRF-31-PSQP4PW3FJI0B | -0.077649528 | -0.007760514 | 0.009781813 | 0.518349508 | 0.285377309 |
| 463 | trna17-SupTTA_1 | trna17-SupTTA | -1.248179194 | 0.010136327 | 0.012826795 | 0.518680201 | 0.285100329 |
| 464 | MIMAT0002874 | miR-503-5p | 0.799766326 | -0.005790307 | 0.00733325 | 0.518680201 | 0.285100329 |
| 465 | trna29-ProAGG_1 | trna29-ProAGG | -0.056790189 | -0.005776109 | 0.00740018 | 0.521845707 | 0.282457885 |
| 466 | trna9-ProAGG_1 | trna9-ProAGG_1 | -0.056790189 | -0.005776109 | 0.00740018 | 0.521845707 | 0.282457885 |
| 467 | trna12-ProAGG_1 | trna12-ProAGG | -0.019819213 | -0.00574025 | 0.007355948 | 0.521845707 | 0.282457885 |
| 468 | trna15-TyrGTA_1 | trna15-TyrGTA | -0.96769798 | -0.008077943 | 0.010791219 | 0.543389812 | 0.264888508 |
| 469 | trna7-SerGCT_1 | trna7-SerGCT | 0.663174027 | 0.006718345 | 0.009093407 | 0.549276874 | 0.260208685 |
| 470 | trna23-ProAGG_1 | trna23-ProAGG | -0.047026769 | -0.005378393 | 0.007375953 | 0.553288677 | 0.257048217 |
| 471 | trna2-ProAGG_1 | trna2-ProAGG | -0.047026769 | -0.005378393 | 0.007375953 | 0.553288677 | 0.257048217 |
| 472 | ENST00000365382 | SNORD14C | -0.281795362 | -0.010766109 | 0.014779646 | 0.553288677 | 0.257048217 |
| 473 | trna10-MetCAT_1 | trna10-MetCAT | 0.414130433 | 0.005489364 | 0.007636813 | 0.559126566 | 0.252489872 |
| 474 | trna65-ProAGG_1 | trna65-ProAGG | -0.042313584 | -0.005280178 | 0.007381217 | 0.560461619 | 0.251454123 |
| 475 | trna146-GlnCTG_1 | trna146-GlnCTG | -0.120897807 | 0.011875194 | 0.016834603 | 0.563170159 | 0.249360365 |
| 476 | trna1-GlnCTG_1 | trna1-GlnCTG | -1.513196859 | 0.010592376 | 0.015021076 | 0.563170159 | 0.249360365 |
| 477 | trna49-GlnCTG_1 | trna49-GlnCTG | -1.513196859 | 0.010592376 | 0.015021076 | 0.563170159 | 0.249360365 |
| 478 | trna99-GlnCTG_1 | trna99-GlnCTG | -1.513196859 | 0.010592376 | 0.015021076 | 0.563170159 | 0.249360365 |
| 479 | trna62-SerGCT_1 | trna62-SerGCT | 0.615497237 | 0.006734872 | 0.009644483 | 0.566992633 | 0.246422584 |
| 480 | ENST00000385059 | MIR451A | -0.192061406 | 0.006553087 | 0.009640823 | 0.579460032 | 0.236976514 |
| 481 | trna32-MetCAT_1 | trna32-MetCAT | 0.266628597 | -0.005166754 | 0.007761523 | 0.588652471 | 0.230141028 |
| 482 | trna129-MetCAT_1 | trna129-MetCAT | 0.013645848 | 0.005796696 | 0.009013788 | 0.604340328 | 0.218718424 |
| 483 | ENST00000619225 | U2_3 | 0.068579078 | -0.008995651 | 0.014342655 | 0.615108383 | 0.211048354 |
| 484 | MIMAT0000721 | miR-369-3p | 4.187792057 | -0.005270061 | 0.008573415 | 0.62335195 | 0.205266678 |
| 485 | MIMAT0000064 | let-7c-5p | 1.076903717 | -0.004069737 | 0.006855096 | 0.638198939 | 0.195043922 |
| 486 | trna20-MetCAT_2 | trna20-MetCAT | 0.004420142 | 0.005148591 | 0.009094947 | 0.654284087 | 0.184233642 |
| 487 | trna142-MetCAT_1 | trna142-MetCAT | 0.004420142 | 0.005148591 | 0.009094947 | 0.654284087 | 0.184233642 |
| 488 | trna169-MetCAT_1 | trna169-MetCAT | 0.004420142 | 0.005148591 | 0.009094947 | 0.654284087 | 0.184233642 |
| 489 | trna171-MetCAT_1 | trna171-MetCAT | 0.004420142 | 0.005148591 | 0.009094947 | 0.654284087 | 0.184233642 |
| 490 | trna41-SerCGA_1 | trna41-SerCGA | -0.97057711 | -0.006451504 | 0.011668291 | 0.663103803 | 0.178418481 |
| 491 | MIMAT0004757 | miR-431-3p | -0.135560086 | -0.005459963 | 0.009903059 | 0.663103803 | 0.178418481 |
| 492 | MIMAT0003338 | miR-660-5p | 1.840809678 | -0.004627234 | 0.008510853 | 0.667740114 | 0.175392533 |
| 493 | trna5-CysGCA_1 | trna5-CysGCA | -1.160314891 | 0.007085838 | 0.013114785 | 0.669040284 | 0.174547732 |
| 494 | MIMAT0031179 | miR-7976 | -0.915984951 | -0.005588961 | 0.010384793 | 0.669333642 | 0.174357346 |
| 495 | trna150-MetCAT_1 | trna150-MetCAT | 0.096327075 | 0.004443628 | 0.008585289 | 0.684158157 | 0.164843491 |
| 496 | trna149-LysTTT_1 | trna149-LysTTT | -0.979102103 | -0.0054247 | 0.010700138 | 0.690208175 | 0.161019901 |
| 497 | MIMAT0004762_1 | miR-486-3p | 2.41169632 | 0.004270808 | 0.008433283 | 0.690208175 | 0.161019901 |
| 498 | trna6-AspGTC_1 | trna6-AspGTC | -0.861274769 | 0.005797774 | 0.011872201 | 0.703151832 | 0.152950887 |
| 499 | trna11-ProAGG_1 | trna11-ProAGG | -0.799475438 | -0.004932066 | 0.010372837 | 0.712003105 | 0.147518112 |
| 500 | trna3-ArgCCT_1 | trna3-ArgCCT | -1.010095334 | 0.005192478 | 0.011150746 | 0.716998387 | 0.144481822 |
| 501 | trna2-SerTGA_1 | trna2-SerTGA | 1.634646941 | -0.003053127 | 0.006533435 | 0.716998387 | 0.144481822 |
| 502 | MIMAT0002171 | miR-410-3p | 2.542906985 | -0.003517738 | 0.007606052 | 0.718103516 | 0.143812947 |
| 503 | trna2-MetCAT_1 | trna2-MetCAT | 0.012364791 | 0.00415888 | 0.009032437 | 0.718315992 | 0.143684465 |
| 504 | MIMAT0004775 | miR-502-3p | 2.238323119 | -0.002105249 | 0.004643071 | 0.722497975 | 0.141163366 |
| 505 | trna120-AlaAGC_1 | trna120-AlaAGC | -0.992017252 | -0.004557219 | 0.01076567 | 0.74177415 | 0.129728305 |
| 506 | ENST00000384147 | SNORD26 | 0.729086181 | -0.00900196 | 0.021220869 | 0.74177415 | 0.129728305 |
| 507 | ENST00000612732 | 5_5 | -1.034373805 | 0.005832759 | 0.013815876 | 0.74177415 | 0.129728305 |
| 508 | MIMAT0000460_1 | miR-194-5p | -0.231183192 | -0.003919162 | 0.009264994 | 0.74177415 | 0.129728305 |
| 509 | MIMAT0004909 | miR-450b-5p | 1.295193169 | -0.003011029 | 0.007179896 | 0.742573352 | 0.12926064 |
| 510 | trna33-ProAGG_1 | trna33-ProAGG | -0.14167884 | -0.003967716 | 0.009548617 | 0.744202174 | 0.128309065 |
| 511 | trna11-PheGAA_1 | trna11-PheGAA | -1.348951726 | 0.005571342 | 0.013506021 | 0.7451694 | 0.127744988 |
| 512 | trna80-GluCTC_1 | trna80-GluCTC | 3.165003767 | -0.002206158 | 0.005445864 | 0.74965491 | 0.12513861 |
| 513 | trna61-MetCAT_1 | trna61-MetCAT | 0.019424907 | 0.004011017 | 0.010109242 | 0.754896424 | 0.122112632 |
| 514 | trna2-GlyTCC_2 | trna2-GlyTCC | 0.347581243 | 0.003211655 | 0.008556734 | 0.770719305 | 0.113103763 |
| 515 | MIMAT0000454 | miR-184 | 0.483125529 | 0.005128712 | 0.014036403 | 0.777283215 | 0.109420711 |
| 516 | trna71-GluCTC_1 | trna71-GluCTC | 3.166329889 | -0.001941709 | 0.005417694 | 0.77993053 | 0.107944079 |
| 517 | MIMAT0004810 | miR-629-5p | 2.312675077 | 0.001832917 | 0.005092493 | 0.77993053 | 0.107944079 |
| 518 | trna77-GluCTC_2 | trna77-GluCTC | 3.161921527 | -0.001914177 | 0.005414494 | 0.782370167 | 0.106587718 |
| 519 | trna35-SerCGA_1 | trna35-SerCGA | -0.784705479 | 0.003980213 | 0.011364143 | 0.783521173 | 0.105949263 |
| 520 | ENST00000364451 | RNA5S9 | -1.189024785 | -0.003915974 | 0.011412251 | 0.787763688 | 0.103604042 |
| 521 | ENST00000410694 | RNU2-48P | -1.233953278 | -0.005185295 | 0.015776991 | 0.79798524 | 0.098005141 |
| 522 | trna2-GlyGCC_1 | trna2-GlyGCC | 1.360736502 | 0.002711127 | 0.008327502 | 0.798970845 | 0.097469068 |
| 523 | tRF-27-PS5P4PW3FJI | tRF-27-PS5P4PW3FJI | 0.358106876 | 0.002744438 | 0.008538908 | 0.800815358 | 0.096467607 |
| 524 | trna3-GlnCTG_1 | trna3-GlnCTG_1 | -1.463841207 | 0.00464144 | 0.015156841 | 0.81160621 | 0.090654639 |
| 525 | trna74-GluCTC_1 | trna74-GluCTC | 3.16110835 | -0.001588708 | 0.005411213 | 0.820223394 | 0.086067848 |
| 526 | MIMAT0004701 | miR-338-5p | 1.38608727 | -0.001569079 | 0.005376868 | 0.820223394 | 0.086067848 |
| 527 | MIMAT0000437 | miR-145-5p | -0.454970974 | 0.002793065 | 0.009721007 | 0.822323636 | 0.084957227 |
| 528 | trna77-GluCTC_1 | trna77-GluCTC_1 | 3.198856681 | -0.001457516 | 0.005325734 | 0.831870414 | 0.079944321 |
| 529 | trna67-AlaAGC_1 | trna67-AlaAGC | -0.168225629 | 0.004938391 | 0.018750869 | 0.838072413 | 0.076718455 |
| 530 | trna87-GluCTC_1 | trna87-GluCTC | 3.147059013 | -0.001421812 | 0.005422848 | 0.838072413 | 0.076718455 |
| 531 | trna39-GlyGCC_1 | trna39-GlyGCC | 1.350639254 | 0.00212836 | 0.008315734 | 0.839993963 | 0.075723835 |
| 532 | trna100-LeuCAA_1 | trna100-LeuCAA | -1.500924429 | -0.002988631 | 0.011672517 | 0.839993963 | 0.075723835 |
| 533 | trna23-ArgCCG_1 | trna23-ArgCCG | 0.272841652 | 0.002021669 | 0.008115156 | 0.843956716 | 0.073679826 |
| 534 | MIMAT0004749 | miR-424-3p | 0.724942587 | -0.001872669 | 0.007625698 | 0.845256464 | 0.073011499 |
| 535 | trna35-GlyGCC_1 | trna35-GlyGCC | 1.366599676 | 0.00196078 | 0.008361926 | 0.852671028 | 0.069218493 |
| 536 | trna37-GlyGCC_1 | trna37-GlyGCC | 1.357324281 | 0.001914865 | 0.00832109 | 0.854623779 | 0.068225028 |
| 537 | MIMAT0001341 | miR-424-5p | 2.041214555 | -0.001545872 | 0.007276813 | 0.8673903 | 0.061785439 |
| 538 | trna78-LeuAAG_1 | trna78-LeuAAG | -0.909021597 | -0.002320673 | 0.011864042 | 0.879469442 | 0.055779246 |
| 539 | MIMAT0000705 | miR-362-5p | 0.77705099 | -0.001079726 | 0.006982597 | 0.90901566 | 0.041428635 |
| 540 | MIMAT0001625 | miR-431-5p | -0.088348472 | 0.001500253 | 0.009787389 | 0.90901566 | 0.041428635 |
| 541 | MIMAT0000436 | miR-144-3p | 7.86907878 | -0.000646999 | 0.00414664 | 0.90901566 | 0.041428635 |
| 542 | MIMAT0005792_1 | miR-320b | 2.364252063 | 0.000616347 | 0.00414164 | 0.909301805 | 0.041291947 |
| 543 | MIMAT0000097 | miR-99a-5p | 5.248601673 | 0.000529604 | 0.003510281 | 0.909301805 | 0.041291947 |
| 544 | trna16-ValTAC_1 | trna16-ValTAC | -0.33417193 | -0.001399134 | 0.010184674 | 0.916931295 | 0.037663204 |
| 545 | trna41-GlyGCC_1 | trna41-GlyGCC | 1.359650009 | 0.001116309 | 0.008319561 | 0.917846344 | 0.037230017 |
| 546 | trna10-GlyTCC_1 | trna10-GlyTCC | -1.216835153 | -0.001580422 | 0.012734164 | 0.924337567 | 0.034169396 |
| 547 | trna8-SeC(e)TCA_1 | trna8-SeC(e)TCA | 1.936632079 | 0.000907426 | 0.007951199 | 0.927355082 | 0.032753944 |
| 548 | MIMAT0000423_1 | miR-125b-5p | 3.576162152 | 0.000444754 | 0.003796749 | 0.927355082 | 0.032753944 |
| 549 | MIMAT0005892 | miR-1304-5p | -1.349704247 | 0.001353977 | 0.011781678 | 0.927355082 | 0.032753944 |
| 550 | MIMAT0005793_1 | miR-320c | 1.13770132 | 0.00062756 | 0.005938583 | 0.932491815 | 0.030354971 |
| 551 | trna3-GlnCTG_2 | trna3-GlnCTG | -0.644733069 | -0.001177171 | 0.011600222 | 0.934184443 | 0.02956737 |
| 552 | tRF-32-PSQP4PW3FJI01 | tRF-32-PSQP4PW3FJI01 | 0.706074442 | -0.000650815 | 0.008746608 | 0.954319109 | 0.02030638 |
| 553 | MIMAT0000457 | miR-188-5p | 0.897152036 | -0.000401664 | 0.0064864 | 0.962656565 | 0.016528623 |
| 554 | trna3-AlaAGC_1 | trna3-AlaAGC | 0.206710204 | 0.000346261 | 0.007877808 | 0.972701959 | 0.01202021 |
| 555 | trna37-ProCGG_1 | trna37-ProCGG | 0.737005057 | 0.000263708 | 0.006142158 | 0.972701959 | 0.01202021 |
| 556 | ENST00000613956 | U2_2 | 0.594397681 | -0.000372764 | 0.008036749 | 0.972701959 | 0.01202021 |
| 557 | trna13-ValCAC_1 | trna13-ValCAC | 0.889013407 | 0.000301966 | 0.008717166 | 0.977603691 | 0.009837167 |
| 558 | trna6-ProCGG_1 | trna6-ProCGG | 0.722723507 | -6.34849E-05 | 0.006159266 | 0.995330927 | 0.002032501 |
| 559 | ENST00000577887 | SNORD53_1 | 0.466137355 | -4.41343E-05 | 0.009362473 | 0.998020997 | 0.000860322 |
| 560 | trna52-ProCGG_1 | trna52-ProCGG | 0.732851592 | 7.64322E-07 | 0.00611214 | 0.999900225 | 4.3334E-05 |

# **Supplementary Table 6. Top coefficients used by the models.**

| **proteins + top20_miRNAs** |  |  |
| --- | --- | --- |
|  |  |  |
| **feature** | **coef** | **zcoef** |
| intercept | 53.903 | 55.3 |
| HEMO | 0.005 | 5.6 |
| FBLN1 | 0.124 | 3.5 |
| CYTC | 0.243 | 2.5 |
| miR-20a-5p | 0.167 | 1.4 |
| FIBB | 0.002 | 1.3 |
| miR-145-5p | 1.388 | 1.2 |
| miR-382-5p | 0.457 | 1.2 |
| miR-29b-3p | 0.088 | 0.8 |
| CO5 | 0.015 | 0.8 |
| miR-122-5p | 0.001 | 0.1 |
| APOC1 | 0 | 0 |
| FA10 | -0.007 | -0.2 |
| miR-125b-5p | -0.097 | -0.5 |
| ANT3 | -0.004 | -0.5 |
| GELS | -0.018 | -0.7 |
| VASN | -0.071 | -0.8 |
| A2AP | -0.095 | -1.3 |
| FETUA | -0.001 | -1.4 |
| PLMN | -0.118 | -1.8 |
| APOL1 | -0.058 | -2 |
| miR-26a-5p | -0.006 | -2.1 |
| LYAM1 | -0.253 | -2.1 |
| miR-93-5p | -0.124 | -3.2 |
| miR-26b-5p | -0.106 | -5.1 |
|  |  |  |
|  |  |  |
| **proteins + miRNAs** |  |  |
|  |  |  |
| **feature** | **coef** | **zcoef** |
| intercept | 52.82 | 55.3 |
| HEMO | 0.005 | 5.3 |
| FBLN1 | 0.121 | 3.4 |
| CYTC | 0.244 | 2.5 |
| miR-210-3p | 0.401 | 1.5 |
| FIBB | 0.002 | 1.2 |
| miR-370-3p | 0.266 | 1.1 |
| miR-320b | 0.427 | 1.1 |
| miR-501-3p | 0.179 | 0.8 |
| miR-660-5p | 0.213 | 0.8 |
| miR-375 | 0.069 | 0.8 |
| miR-409-5p | 0.545 | 0.6 |
| miR-1260a | 0.331 | 0.5 |
| miR-145-5p | 0.526 | 0.5 |
| miR-589-5p | 0.101 | 0.4 |
| miR-503-5p | 0.244 | 0.4 |
| miR-485-3p | 0.101 | 0.2 |
| miR-148a-5p | 0.179 | 0.2 |
| miR-337-5p | 0.045 | 0.1 |
| miR-380-3p | 0.046 | 0.1 |
| miR-10a-5p | 0.002 | 0 |
| miR-125b-5p | -0.019 | -0.1 |
| miR-92b-3p | -0.04 | -0.1 |
| APOC1 | -0.002 | -0.2 |
| miR-32-5p | -0.099 | -0.3 |
| GELS | -0.01 | -0.4 |
| let-7e-5p | -0.268 | -0.5 |
| ANT3 | -0.003 | -0.5 |
| miR-2355-3p | -0.347 | -0.6 |
| FETUA | 0 | -0.6 |
| miR-184 | -0.265 | -0.6 |
| miR-454-3p | -0.531 | -0.7 |
| miR-93-5p | -0.027 | -0.7 |
| miR-485-5p | -0.87 | -0.9 |
| miR-2355-5p | -0.535 | -1.1 |
| VASN | -0.1 | -1.1 |
| miR-182-5p | -0.139 | -1.1 |
| PLMN | -0.096 | -1.5 |
| miR-18b-5p | -1.064 | -1.5 |
| miR-628-3p | -0.169 | -1.6 |
| miR-26b-5p | -0.034 | -1.7 |
| A2AP | -0.122 | -1.7 |
| APOL1 | -0.063 | -2.1 |
| LYAM1 | -0.261 | -2.2 |
| miR-374a-5p | -0.436 | -3.7 |
|  |  |  |
|  |  |  |
| **proteins + all small RNAs** |  |  |
|  |  |  |
| **feature** | **coef** | **zcoef** |
| intercept | 51.88 | 55.3 |
| HEMO | 0.004 | 4.2 |
| FBLN1 | 0.106 | 3 |
| CYTC | 0.259 | 2.6 |
| tRF-34-JJ6RRNLIK898HR | 0.344 | 1.6 |
| miR-210-3p | 0.309 | 1.2 |
| trna27-LeuTAG | 1.191 | 1.1 |
| miR-320c | 0.608 | 1 |
| trna100-LeuCAA | 1.576 | 0.8 |
| FIBB | 0.001 | 0.8 |
| miR-320b | 0.304 | 0.8 |
| miR-409-5p | 0.608 | 0.7 |
| CO5 | 0.008 | 0.4 |
| miR-145-5p | 0.343 | 0.3 |
| miR-501-3p | 0.063 | 0.3 |
| CO9 | 0.004 | 0.3 |
| miR-375 | 0.022 | 0.2 |
| miR-382-5p | 0.073 | 0.2 |
| tRF-40-2R1HPSR9O9337KB6 | 0.119 | 0.2 |
| trna83-LeuTAA | 0.048 | 0.1 |
| trna140-LeuCAA | 0.256 | 0.1 |
| miR-1260a | 0.034 | 0.1 |
| trna11-GluTTC | 0.016 | 0 |
| miR-2355-3p | -0.04 | -0.1 |
| trna168-TrpCCA | -0.223 | -0.1 |
| trna9-ProAGG | -0.172 | -0.1 |
| miR-30a-3p | -0.118 | -0.1 |
| trna30-ProCGG | -0.414 | -0.2 |
| APOC1 | -0.002 | -0.2 |
| miR-454-3p | -0.213 | -0.3 |
| miR-182-5p | -0.043 | -0.3 |
| trna175-SerGCT | -1.161 | -0.5 |
| trna17-GluTTC | -0.781 | -0.6 |
| let-7e-5p | -0.453 | -0.8 |
| ANT3 | -0.006 | -0.9 |
| trna3-AlaAGC | -0.792 | -0.9 |
| miR-18b-5p | -0.693 | -1 |
| miR-93-5p | -0.04 | -1 |
| trna106-PheGAA | -0.259 | -1.1 |
| miR-485-5p | -1.082 | -1.1 |
| A2AP | -0.09 | -1.2 |
| PLMN | -0.081 | -1.2 |
| miR-628-3p | -0.144 | -1.3 |
| FETUA | -0.001 | -1.3 |
| APOL1 | -0.041 | -1.4 |
| miR-2355-5p | -0.93 | -1.8 |
| miR-26b-5p | -0.041 | -2 |
| miR-374a-5p | -0.242 | -2.1 |
| LYAM1 | -0.249 | -2.1 |
|  |  |  |
|  |  |  |
| **proteins + tRFs** |  |  |
|  |  |  |
| **feature** | **coef** | **zcoef** |
| intercept | 38.509 | 55.3 |
| HEMO | 0.006 | 6 |
| FBLN1 | 0.143 | 4 |
| CYTC | 0.358 | 3.6 |
| tRF-34-JJ6RRNLIK898HR | 0.472 | 2.2 |
| tRF-40-2R1HPSR9O9337KB6 | 1.158 | 1.7 |
| CO5 | 0.011 | 0.6 |
| CO9 | 0.008 | 0.5 |
| FIBB | 0.001 | 0.4 |
| tRF-32-P4R8YP9LON4V3 | 0.001 | 0 |
| ANT3 | 0 | 0 |
| BTD | -0.086 | -0.5 |
| VASN | -0.062 | -0.7 |
| tRF-19-B1RHODE2 | -0.476 | -0.7 |
| PLMN | -0.061 | -0.9 |
| APOC1 | -0.01 | -1.1 |
| A2AP | -0.086 | -1.2 |
| FETUA | -0.001 | -1.8 |
| GELS | -0.044 | -1.8 |
| APOL1 | -0.053 | -1.8 |
| LYAM1 | -0.242 | -2 |
| tRF-18-BS68BFD2 | -0.923 | -3.7 |
|  |  |  |
|  |  |  |
| **proteins** |  |  |
|  |  |  |
| **feature** | **coef** | **zcoef** |
| intercept | 37.05 | 55.3 |
| HEMO | 0.006 | 6.9 |
| FBLN1 | 0.16 | 4.5 |
| CYTC | 0.416 | 4.2 |
| CO5 | 0.04 | 2.1 |
| CFAB | 0.035 | 2 |
| FIBB | 0.002 | 0.9 |
| AFAM | 0.024 | 0.8 |
| CO9 | 0.011 | 0.7 |
| C1S | 0.018 | 0.5 |
| CLUS | 0.009 | 0.4 |
| PHLD | 0.009 | 0.2 |
| FIBA | 0 | -0.1 |
| CFAH | -0.011 | -1.4 |
| FETUA | -0.001 | -1.7 |
| VASN | -0.169 | -1.8 |
| GELS | -0.047 | -1.9 |
| APOC1 | -0.018 | -2 |
| PLMN | -0.13 | -2 |
| A2AP | -0.193 | -2.6 |
| APOL1 | -0.083 | -2.8 |
| LYAM1 | -0.376 | -3.2 |
|  |  |  |
|  |  |  |
| **top20_miRNAs** |  |  |
|  |  |  |
| **feature** | **coef** | **zcoef** |
| intercept | 70.351 | 55.3 |
| miR-20a-5p | 0.951 | 8 |
| miR-29b-3p | 0.51 | 4.7 |
| miR-23b-3p | 0.208 | 1.8 |
| miR-145-5p | 1.995 | 1.7 |
| miR-382-5p | 0.482 | 1.3 |
| miR-148a-3p | 0.016 | 1.1 |
| miR-30b-5p | -0.241 | -0.5 |
| miR-125b-5p | -0.164 | -0.9 |
| miR-150-5p | -0.07 | -3.4 |
| miR-106b-5p | -0.336 | -3.7 |
| miR-26a-5p | -0.017 | -6.3 |
| miR-26b-5p | -0.137 | -6.6 |
| miR-93-5p | -0.324 | -8.3 |
|  |  |  |
|  |  |  |
| **miRNAs** |  |  |
|  |  |  |
| **feature** | **coef** | **zcoef** |
| intercept | 69.571 | 55.3 |
| miR-93-5p | -0.007 | -0.2 |
| miR-2355-5p | -0.459 | -0.9 |
| miR-200c-3p | -0.702 | -1.1 |
| miR-26b-5p | -0.037 | -1.8 |
| miR-30e-3p | -0.181 | -2 |
| miR-374a-5p | -0.595 | -5.1 |
|  |  |  |
|  |  |  |
| **all small RNAs** |  |  |
|  |  |  |
| **feature** | **coef** | **zcoef** |
| intercept | 71.707 | 55.3 |
| tRF-27-PS5P4PW3FJI | 1.236 | 1.9 |
| miR-210-3p | 0.457 | 1.7 |
| miR-410-3p | 0.182 | 1.1 |
| miR-375 | 0.07 | 0.8 |
| trna27-LeuTAG | 0.686 | 0.7 |
| trna105-AlaAGC | 0.821 | 0.6 |
| miR-503-5p | 0.205 | 0.3 |
| tRF-40-2R1HPSR9O9337KB6 | 0.235 | 0.3 |
| tRF-34-JJ6RRNLIK898HR | 0.06 | 0.3 |
| miR-362-5p | 0.129 | 0.2 |
| trna33-HisGTG | 0.426 | 0.2 |
| miR-320c | 0.12 | 0.2 |
| miR-450b-5p | 0.069 | 0.2 |
| miR-501-3p | 0.028 | 0.1 |
| trna13-AlaTGC | 0.195 | 0.1 |
| trna100-LeuCAA | 0.213 | 0.1 |
| miR-431-5p | 0.059 | 0.1 |
| trna7-HisGTG | 0.006 | 0 |
| trna3-AlaAGC | -0.013 | 0 |
| miR-224-5p | -0.002 | 0 |
| U2_2 | -0.022 | 0 |
| trna78-LeuAAG | -0.08 | -0.1 |
| miR-485-5p | -0.096 | -0.1 |
| miR-100-5p | -0.004 | -0.1 |
| trna113-AlaTGC | -0.512 | -0.2 |
| miR-155-5p | -0.112 | -0.3 |
| trna106-PheGAA | -0.096 | -0.4 |
| miR-411-3p | -0.65 | -0.9 |
| trna30-ProCGG | -1.906 | -0.9 |
| trna175-SerGCT | -2.264 | -1.1 |
| miR-26b-5p | -0.022 | -1.1 |
| trna17-GluTTC | -1.859 | -1.5 |
| miR-200c-3p | -1.009 | -1.5 |
| miR-30e-3p | -0.151 | -1.6 |
| miR-2355-5p | -0.893 | -1.8 |
| trna99-ValCAC | -0.739 | -1.8 |
| miR-93-5p | -0.116 | -3 |
| miR-374a-5p | -0.607 | -5.2 |
|  |  |  |
|  |  |  |
| **tRNAs** |  |  |
|  |  |  |
| **feature** | **coef** | **zcoef** |
| intercept | 60.945 | 55.3 |
| trna11-GluTTC | 1.223 | 3 |
| trna84-GluTTC | 2.164 | 2.8 |
| trna27-LeuTAG | 1.431 | 1.4 |
| trna38-IleAAT | 0.507 | 0.5 |
| trna33-HisGTG | 0.735 | 0.4 |
| trna2-ArgCCT | 0.365 | 0.2 |
| trna15-ValAAC | 0.035 | 0.1 |
| trna134-GluTTC | 0.024 | 0 |
| trna18-GlyGCC | 0 | 0 |
| trna133-GlyCCC | 0 | 0 |
| trna4-GlyCCC | -0.003 | -0.1 |
| trna4-LeuTAA | -0.824 | -0.4 |
| trna13-PheGAA | -0.981 | -0.6 |
| trna10-AlaCGC | -1.163 | -0.8 |
| trna30-ProCGG | -1.971 | -1 |
| trna73-GlyTCC | -3.348 | -1.6 |
| trna9-ProAGG | -2.229 | -1.7 |
| trna17-GluTTC | -3.111 | -2.5 |
| trna6-ValCAC | -0.599 | -3.2 |
| trna175-SerGCT | -8.631 | -4 |
|  |  |  |
|  |  |  |
| **tRFs** |  |  |
|  |  |  |
| **feature** | **coef** | **zcoef** |
| intercept | 57.866 | 55.3 |
| tRF-40-2R1HPSR9O9337KB6 | 2.353 | 3.4 |
| tRF-34-JJ6RRNLIK898HR | 0.493 | 2.3 |
| tRF-27-PS5P4PW3FJI | 1.281 | 1.9 |
| tRF-40-2VR008R959KUMKF6 | 0.191 | 0.8 |
| tRF-19-FMD0SR1Z | 0.297 | 0.7 |
| tRF-18-0P583004 | -0.318 | -0.5 |
| tRF-18-YRRHQFD2 | -0.328 | -0.6 |
| tRF-32-87R8WP9N1EWJM | -0.02 | -0.8 |
| tRF-19-B1RHODE2 | -0.902 | -1.4 |
| tRF-23-ZKXU53K80E | -2.113 | -2 |
| tRF-18-BS68BFD2 | -1.52 | -6.2 |
|  |  |  |
|  |  |  |
| **snoRNAs** |  |  |
|  |  |  |
| **feature** | **coef** | **zcoef** |
| intercept | 54.898 | 55.3 |
| SNORD26 | 0.854 | 3.8 |
| SNORD53_1 | 0.851 | 1.5 |
| snoU18 | -1.359 | -7.5 |
|  |  |  |
|  |  |  |
| **rRNAs** |  |  |
|  |  |  |
| **feature** | **coef** | **zcoef** |
| intercept | 58.628 | 55.3 |
| RNA5SP194 | 0.629 | 2.4 |
| RNA5-8S5_2 | -0.256 | -2.4 |
