# Supplementary material for: Age prediction from human blood plasma using proteomic and small RNA data: a comparative analysis
Source: Aging (Albany NY). 2023 Jun 20;15(12):5240–65. doi: 10.18632/aging.204787 (PMC10333066; doi:10.18632/aging.204787)
Supplement: Supplementary Tables 2 and 4-5 [file aging-15-204787-s003.pdf]

SUPPLEMENTARY TABLES

Supplementary Table 2. Results of the MRM-MS.

#	Uniprot_id	Protein_name	Gene_name	Protein_description	Beta	FDR	minusLog10_FDR
1	P02748	CO9	C9	Complement component C9	0.01620584	5.9925E-08	7.22239447
2	P23142	FBLN1	FBLN1	Fibulin-1	0.01141984	3.8453E-07	6.41506685
3	P01034	CYTC	CST3	Cystatin-C	0.01093426	1.0384E-06	5.98363021
4	P01031	CO5	C5	Complement C5	0.00976814	3.9602E-06	5.4022833
5	P02790	HEMO	HPX	Hemopexin	0.00740283	5.5291E-06	5.2573473
6	P05156	CFAI	CFI	Complement factor I	0.01027467	1.1228E-05	4.94969539
7	P02671	FIBA	FGA	Fibrinogen alpha chain	0.01127861	2.2016E-05	4.65726498
8	P02750	A2GL	LRG1	Leucine-rich alpha-2-glycoprotein	0.01081106	3.4839E-05	4.45793726
9	P09871	C1S	C1S	Complement C1s subcomponent	0.00589129	0.00069008	3.16109998
10	P02654	APOC1	APOC1	Apolipoprotein C-I	−0.01537613	0.00079773	3.09814587
11	P02679	FIBG	FGG	Fibrinogen gamma chain	0.00772761	0.00079773	3.09814587
12	P02675	FIBB	FGB	Fibrinogen beta chain	0.00740994	0.00129809	2.88669453
13	P02652	APOA2	APOA2	Apolipoprotein A-II	−0.00633054	0.00427695	2.36886632
14	P01008	ANT3	SERPINC1	Antithrombin-III	−0.00498125	0.00527033	2.27816214
15	P08697	A2AP	SERPINF2	Alpha-2-antiplasmin	−0.00404854	0.02922722	1.53421248
16	P14151	LYAM1	SELL	L-selectin	−0.00406082	0.03029594	1.51861562
17	P10909	CLUS	CLU	Clusterin	0.00304876	0.07427541	1.12915495
18	P80108	PHLD	GPLD1	Phosphatidylinositol-glycan-specific phospholipase D	0.00453143	0.07427541	1.12915495
19	O14791	APOL1	APOL1	Apolipoprotein L1	−0.00341748	0.14470684	0.83951093
20	P08603	CFAH	CFH	Complement factor H	0.00443368	0.15273262	0.81606818
21	P01024	CO3	C3	Complement C3	0.0028188	0.16203001	0.79040455
22	P43652	AFAM	AFM	Afamin	0.00325797	0.24156889	0.616959
23	P43251	BTD	BTD	Biotinidase	−0.00244123	0.26084979	0.58360951
24	P00747	PLMN	PLG	Plasminogen	−0.00168636	0.40122892	0.39660777
25	P27169	PON1	PON1	Serum paraoxonase/arylesterase 1	−0.00220693	0.46017138	0.3370804
26	P02765	FETUA	AHSG	Alpha-2-HS-glycoprotein	−0.00153741	0.52045478	0.283617
27	Q6EMK4	VASN	VASN	Vasorin	0.00089481	0.70632444	0.15099577
28	P00734	THRB	F2	Prothrombin	−0.00040461	0.89749857	0.04696624
29	P06396	GELS	GSN	Gelsolin	−0.00023148	0.94002158	0.02686218
30	P00742	FA10	F10	Coagulation factor X	0.00022134	0.94002158	0.02686218
31	P00751	CFAB	CFB	Complement factor B	−2.7028E-05	0.98989909	0.00440907

Detailed results of age-association for each protein measured in the MRM-MS analysis.

Supplementary Table 4. Datasets and their performances.

Dataset	Number of features	Number of non-zero coefficients	Lambda	Rsquared	RsquaredSD	MAE	MAESD	RMSE	RMSESD
Proteins + top20_miRNAs	51	24	0.441	0.73	0.01	7.40	0.18	9.11	0.22
Proteins + miRNAs	319	44	0.542	0.70	0.02	7.38	0.28	9.50	0.32
Proteins + all small RNAs	639	48	0.667	0.67	0.04	7.76	0.37	10.05	0.53
Proteins	31	21	0.358	0.59	0.02	9.00	0.25	11.20	0.28
Top20_miRNAs	20	13	0.237	0.54	0.02	9.44	0.19	11.92	0.30
miRNAs	288	6	2.841	0.45	0.02	10.93	0.13	13.12	0.15
All small RNAs	608	38	1.241	0.42	0.03	10.32	0.29	13.26	0.33
tRNAs	229	20	1.526	0.28	0.03	12.20	0.24	14.84	0.28
tRFs	32	11	1.009	0.22	0.03	12.32	0.25	15.43	0.35
snRNAs	20	0	6.504	0.09	0.03	14.57	0.06	17.63	0.07
snoRNAs	16	3	0.667	0.007	0.008	14.433	0.263	18.125	0.533
rRNAs	23	2	0.667	0.006	0.005	14.362	0.139	17.546	0.155

Performance for age-predictions as estimated from the cross-validation analyses. The mean and standard deviation for R^2, MAE and RMSE (Root Mean Square Error) are indicated. The column ‘number of non-zero coefficients’ indicates how many features were retained in the respective model.

Supplementary Table 5. Correlation between models’ prediction errors.

	Proteins	All small RNAs	tRFs	tRNAs	miRNAs	Top20_miRNAs
**proteins**	1	0.373919054	0.435471522	0.440753051	0.409886692	0.250330152
**all small RNAs**	0.373919054	1	0.840188201	0.828969882	0.953634873	0.846409428
**tRFs**	0.435471522	0.840188201	1	0.869324569	0.892236098	0.712732738
**tRNAs**	0.440753051	0.828969882	0.869324569	1	0.867817341	0.695747075
**miRNAs**	0.409886692	0.953634873	0.892236098	0.867817341	1	0.838047513
**top20_miRNAs**	0.250330152	0.846409428	0.712732738	0.695747075	0.838047513	1

Correlation of delta ages (prediction errors) of models built from proteomics or small RNA measurements.
